# A collection of non-human primate computed tomography scans housed in MorphoSource, a repository for 3D data

**DOI:** 10.1038/sdata.2016.1

**Published:** 2016-02-02

**Authors:** Lynn E. Copes, Lynn M. Lucas, James O. Thostenson, Hopi E. Hoekstra, Doug M. Boyer

**Affiliations:** 1 Institute of Human Origins and School of Human Evolution and Social Change, Arizona State University, Tempe, Arizona, USA; 2 Department of Electrical Engineering, Duke University, Durham, North Carolina, USA; 3 Department of Organismic and Evolutionary Biology, Museum of Comparative Zoology, Harvard University, Cambridge, Massachusetts, USA; 4 Department of Evolutionary Anthropology, Duke University, Durham, North Carolina, USA

**Keywords:** Biological anthropology, Databases, Bone

## Abstract

A dataset of high-resolution microCT scans of primate skulls (crania and mandibles) and certain postcranial elements was collected to address questions about primate skull morphology. The sample consists of 489 scans taken from 431 specimens, representing 59 species of most Primate families. These data have transformative reuse potential as such datasets are necessary for conducting high power research into primate evolution, but require significant time and funding to collect. Similar datasets were previously only available to select research groups across the world. The physical specimens are vouchered at Harvard’s Museum of Comparative Zoology. The data collection took place at the Center for Nanoscale Systems at Harvard. The dataset is archived on MorphoSource.org. Though this is the largest high fidelity comparative dataset yet available, its provisioning on a web archive that allows unlimited researcher contributions promises a future with vastly increased digital collections available at researchers’ finger tips.

## Background & Summary

### Digital data in comparative morphology

High fidelity, microCT and surface scan renderings of osteological materials and wet specimens have become essential starting points for basic research in many subfields of evolutionary biology over the last two decades. There are at least three reasons for this: strict limitations on what can be measured and how precisely those measurements can be obtained working from physical specimens, the fact that not all morphology can be measured externally, and the fact that museum specimens are often fragile, and frequent handling can damage specimens. CT scans represent a digitization modality that can be used to visualize and quantify internal morphology, and allow for the creation of 3D surface scans from which quantifications of morphological structures can be derived without being constrained or limited by complexity of the desired morphological structure, or absolute size of the specimen. The need for such data is so critical that (1) meeting demands from researchers for borrowing specimens for scanning has presented a significant challenge to museum staff (pers. comm. R. Voss, American Museum of Natural History, Dept. of Mammalogy); (2) the majority of budgets for many dissertation level projects is now targeted for scanning equipment, facility fees, or software; (3) researchers often spend the majority of their time traveling, scanning and processing datasets, which is traded for time that could have been put into research design and/or analysis.

Despite this rush to digitize, comparative morphology is experiencing a crisis as a mode of addressing large-scale evolutionary questions due to the difficulty involved in accruing datasets large enough to have high explanatory power, and the small community of researchers that can participate effectively. This presents a paradox: If so many researchers are putting large efforts into scanning, where are the massive samples? Though a few research groups have managed to generate large samples of scans comprehensively representing diversity in one clade or another^1^, this work has been time consuming, and expensive: as a result these scans are not made widely accessible to non-collaborating researchers. This inequality in access to what is now essential, basic data clearly falls short of scientific ideals for meritocracy. Furthermore, a significant component of the unmanageable demand for 3D scan data experienced by museums may represent wasteful recollection of data already held by other research groups.

Comparative morphology can be revitalized by democratizing access to microCT scans of specimens in vouchered in museum collections, broadening the community of researchers who can participate in morphological research, and ultimately allowing it join the ranks of other big data science initiatives. In order for this to happen, an infrastructure of efficiently accessing and distributing large numbers of scans is necessary, and researchers and museums must yield their scan collections to it, either voluntarily or through stricter policies from museums and/or funding agencies. The voluntary option is more preferable, but indicates a different incentive structure. Researchers and museums must be able to directly benefit academically and institutionally from third party use of their scans. MorphoSource, the data archive used for this work, addresses this problem by allowing DOI assignment to individual scans and providing usage statistics on each scan.

### Nonhuman primate microCT scans

In this paper we announce an openly accessible microCT data sample that—by itself—can catalyse a small transformation in research on primates because it is the first of its kind. Non-human primate skulls are some of the most frequently examined specimens in natural history museums. Anthropologists and mammalogists alike study aspects of morphology in the skulls of members of the human order Primates to answer comparative, taxonomic, phylogenetic, behavioural, biomechanical, and physiological questions. Two of us (LC and LL) collaborated to collect microCT scans of 431 adult and juvenile non-human primate skulls from the Museum of Comparative Zoology at Harvard University for use in our respective dissertations. We have uploaded the scans to MorphoSource.org (see Data Record), an online repository for 3D data, so others can use the scans for their research projects. This being the first openly accessible primate dataset of its kind, we hope it will encourage other researchers to make their own datasets available in equivalent ways, ideally through MorphoSource as well.

## Methods

A total of 431 skulls of adult and juvenile non-human primates housed at the Museum of Comparative Zoology at Harvard University were microCT (μCT) scanned at Harvard’s Center for Nanoscale Systems. A femur and humerus from some individuals was also scanned. Adulthood was determined by full eruption of the permanent third molars and canines. Any specimen with signs of bony pathology that might have impacted vault or facial growth was excluded. Specimens listed as captive were also not included. Specimens included in the final analyses came from 59 species representing all major families in the order Primates. The only major groups not included are *Phaner*, *Mirza*, A*llocebus*, and *Cheirogaleus* of the Cheirogaleiidae, *Lepilemur* of the Lepilemuridae, or any genus of Daubentoniidae or Tarsiidae. A list of all available specimens, with scanning parameters, is provided in Table 1 (available online only).

The scanner at CNS is a X-Tek HMXST225 μCT scanner. The X-ray detector panel is a Perkin Elmer 1621, which provides a 2,000×2,000 pixel and 16 inch×16 inch field of view with a 7.5 frames per second readout and a physical pixel size of 200 microns. The X-ray source is an X-Tek Nikon microfocus open tube with both reflection and transmission targets. The energy settings for each scan ranged between 70–90 kV and 90–125 μA, depending on the size of the specimen. The strepsirrhine, platyrrhine, and smaller catarrhine specimens were scanned without filters, while *Pan, Pongo,* and *Gorilla* specimens were scanned with a tungsten target to minimize beam hardening.

Each skull was placed in a foam holder that was then positioned inside the scanner on a rotating platform. The foam held the skull in place while still allowing the X-rays to fully penetrate the specimen without leaving visual artefacts. All crania in this study were scanned at parameters optimum for the highest possible resolution within the time available to capture all samples. All crania were scanned using 1,000–1,500 projections, scan time per specimen ranged from 18–60 min, and cubic voxel dimensions ranged from 18 microns for smaller specimens (e.g., *Microcebus*) to 125 microns for the largest (e.g., *Pongo*) (See Table 1 (available online only) for all sample scanning parameters).

Each scan was saved as DICOM files, zipped using either the built-in zip function in OSX (scans <4 GB) or 7zip (scans >4 GB) and uploaded to MorphoSource. Other contributors should note that standard archiving software, such as that available in OSX, will appear to successfully archive files larger than 4 GB of material, but the archive will be unusable once uploaded to MorphoSource. Thus, any file collection greater than 4 GB should be zipped with WinZip or 7zip, which will use the appropriate ZIP64 format to do the zipping. Scans can be downloaded directly by any registered user of the site. Because not all zip file extractors are compatible with the ZIP64 format, we recommend any PC users unzip files greater than 4 GB using WinZip, winRAR or 7zip.

**Figure 1:**
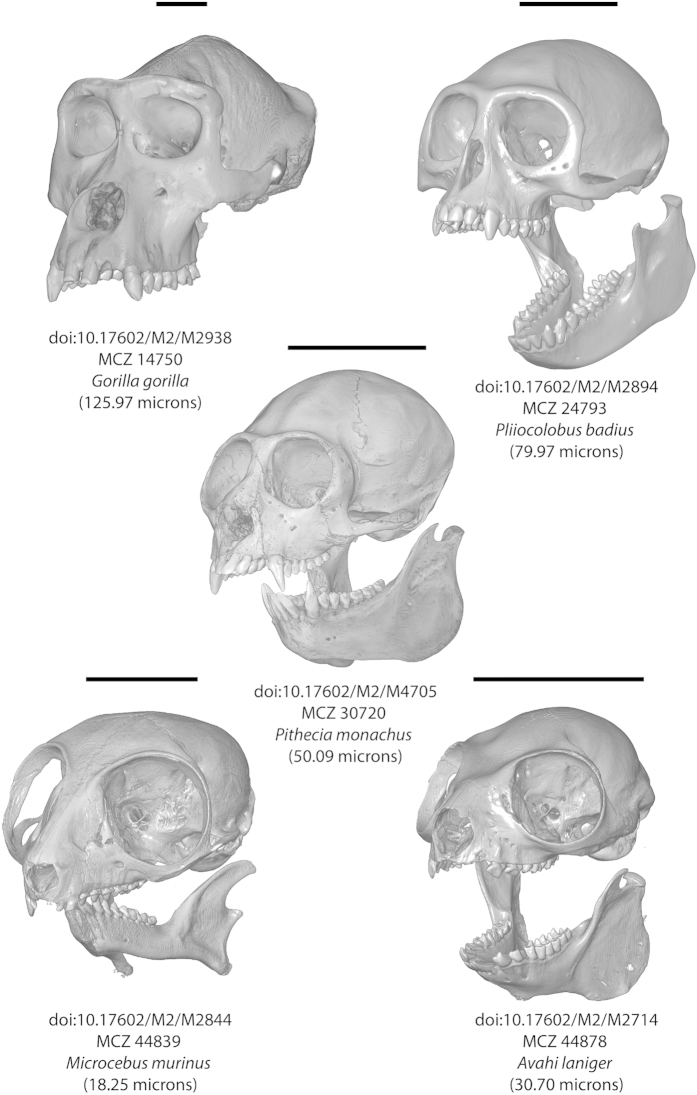
Renderings of voxel data (done with Avizo 8.1) on five of the 489 μCT scan datasets announced in this publication. Note that scans of some specimens include the cranium and the mandible in a single dataset, while for others specimens these elements were scanned separately. The full web links to the DOIs listed in the figure can be found in Data Citations 1–5.

Once downloaded, users are free to collect their own data from the scans. Several examples of surface renderings created from the scans are shown in Figure 1.

## Data Records

### MorphoSource

The microCT data from this project are available through MorphoSource (http://www.MorphoSource.org/). We chose to use MorphoSource because it provides a dynamic archive where microCT datasets continually gain relevance by their incorporation into an ever-expanding digital sample representing collections of multiple researchers and institutions. The site was created to meet the new demand for digital datasets discussed above. Its primary aim is to improve researcher access to relevant comparative samples. MorphoSource is the first project-based data repository for storing, collaborative sharing, and distribution of microCT scans, 3D surface renderings, and 2D digital imagery of specimens. The site has been active since April 2013 (refs 2–4). It currently includes 1,432 registered participants from across the globe and hosts ~8,100 files representing ‘raw’ microCT volumetric data; mesh files (stl, ply) from laser scans, structured light, photogrammetry, or microCT; and 2D digital photographs. These files represent 2,400 repository-vouchered specimens from 73 institutions. The holdings are growing rapidly. Data on the site are protected by creative commons restrictions as customized by each contributing researcher (data author) according to his/her needs, concerns, or third party agreements (e.g., with museums). Most data published on the site can be immediately downloaded by registered users. Other datasets can be released for download upon request, by data authors who retain rights to grant third party access.

The files associated with the current project can be downloaded with open access and are tagged with creative commons copyright license of CC BY-NC as dictated by the copyright holder, the MCZ. This means the data can be downloaded and re-used for non-commercial academic purposes. These limitations are maintained as a component of the non-negotiable terms of the MCZ, the home repository. This framework serves the interests of both physical repositories (museums) and data authors by tracking use statistics on datasets. Such statistics provide evidence of collection value and magnify impact of researcher-collected data. The current MorphoSource dataset is tagged by 489 digital object identifiers (one for each scan, with some specimens represented by multiple scans). As of 1/22/16, the dataset has been viewed more than 31,800 times, and more than 1800 scans have been downloaded. MorphoSource provides search tools to allow users to find, and batch download the samples most relevant to their research design. MorphoSource is free to users and contributors, and the amount of storage space is not explicitly limited. The network storage is distributed between multiple physical locations as part of Duke University’s IT data infrastructure.

Each scan dataset is a ‘media record’ on MorphoSource. The media record includes the metadata on the scan (Table 1 (available online only)) and data files themselves. Searching MorphoSource by specimen will return media records associated those specimens from our data project, as well as media records from other data projects that included digital imagery for those specimens (because other researchers may have scanned and uploaded other bones of the skeleton for the same specimens). Each media record is assigned a digital object identifier DOI, which represents a permanent, direct link to the data and should be cited in any study that uses the scan (in addition to other details—see below).

### Museum of comparative zoology

A copy of the complete microCT dataset is also archived at the MCZ and may be accessible by contacting curators there. As well, 3D pdf files depicting a surface rendering of each skull can be downloaded from the MCZ specimen record pages in the museum’s online database, MCZbase. On MorphoSource, users will find a link to each specimen’s MCZbase page. Researchers should note that these surface renderings are not necessarily to scale currently (whereas all morphosource records are).

### Digitized craniometric data available through Dryad

**Figure 2:**
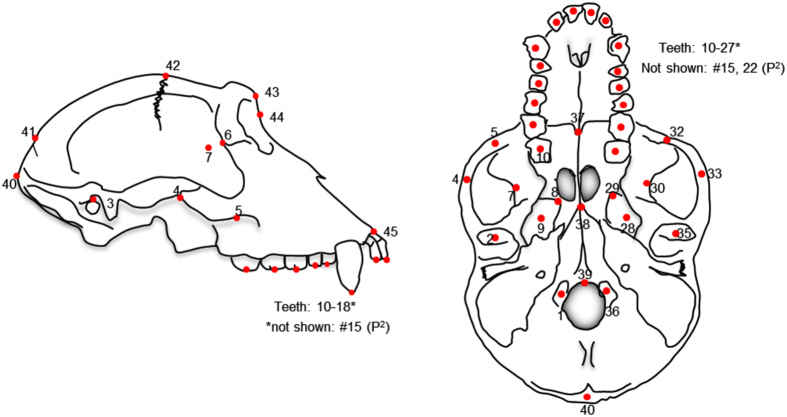
Landmarks digitized on each skull using a MicroScribe. Data are available for download from Dryad (Data Citation 6).

During the process of CT scanning, we also used a 3D digitizer (MicroScribe G2X) to capture 60+ standard craniometric landmarks from each skull, which are illustrated in Figure 2. The points are available to download freely from Dryad, a non-profit repository for data underlying the international scientific and medical literature (Data Citation 6).

In order to gauge inter- and intra-observer error in the craniometric dataset, some specimens were digitized repeatedly. A single researcher (LL) initially digitized all specimens once. Thirty-one specimens were chosen to be re-digitzed three times each by a second researcher (LC). Seven distances were calculated and compared to measure error. Intraobserver error was nearly always <5%, but in some cases, interobserver error exceeded 5%, especially in the smaller specimens. In such cases, it is likely that the two independent researchers disagreed on the exact location of certain landmarks. However, we are confident that the 3D landmark data provided for each specimen is reliable and can be used confidently for future work.

## Technical Validation

Calibrations of non-metrology specific industrial x-ray CT scanners that guarantee a certain minimum error for a particular machine at all settings do not exist, and almost all existing microCT datasets have been collected with such non-metrology specific machines (i.e., all of the major academic scanning facilities in the US have non-metrology specific units: Penn State’s Center for Quantitative Imaging, The University of Texas’ High-Resolution X-ray Computed Tomography Facility, American Museum of Natural History’s Microcopy and Imaging Facility). Industrial x-ray CT scanners that are used for high accuracy (<0.01% error) metrology only calibrate to a single setting at a time (i.e., fixed source, detector and stage settings) with limited ability to scan at different configurations and retain the same accuracy. However, non-metrology scanning facilities (including Harvard’s—the facility where the scans were made) do not typically attempt to record error levels in their voxel sizes. It is assumed that error levels are typically around or below 1% for dimensional based measurements. The accuracy of which is largely determined during the instrument’s initial installation and any further check of calibration done by the facility or other service personnel. However, assuming adequate preventative maintenance has been administered, different machines with the same components and configurations should produce similar errors of around 1% (Greg Lin, personal communication).

We decided to empirically evaluate the expected error under the range of settings used in this study by analyzing results of scanning calibration balls at different resolutions on Duke University’s scanner in the Shared Materials Instrumentation Facility. This scanner (XTH 225ST) is a similar model to the one at Harvard (HMX 225ST). Most importantly they incorporate the same X-ray source (Nikon 225 Reflection tungsten target, with focal spot of 3 um up to 15 W) and detector plate (Perkin Elmer AN1620) with similar range for source-detector distances available (~1.3 m). Therefore our results should correlate with the machine at Harvard as well.

To determine potential error in the scans using a similar scanner, four standard spheres of 3.175 mm (+/−1.0 μm tolerance) were scanned at voxel resolutions of 5, 6, 7, 8, 9, 10, 15 and 20 μm per voxel, and four standard spheres of 12.7 mm (+/−1.0 μm tolerance) at voxel resolutions of 30, 40, 50, 60 and 70 μm per voxel on a Nikon XTH 225 ST. Each scan was collected at 150 keV, 80 μA (12 W), 354 ms, 800 projections and a projection average of 1. No filters were used and the projection data was reconstructed into volumetric data via use of Nikon’s proprietary *CT Pro 3D* and *CT Agent* software. Digital measurements of the standard spheres were performed in Volume Graphics *VG Studio Max 2.2* along with Volume Graphics’s *Coordinate Measurement Module*.

**Figure 3:**
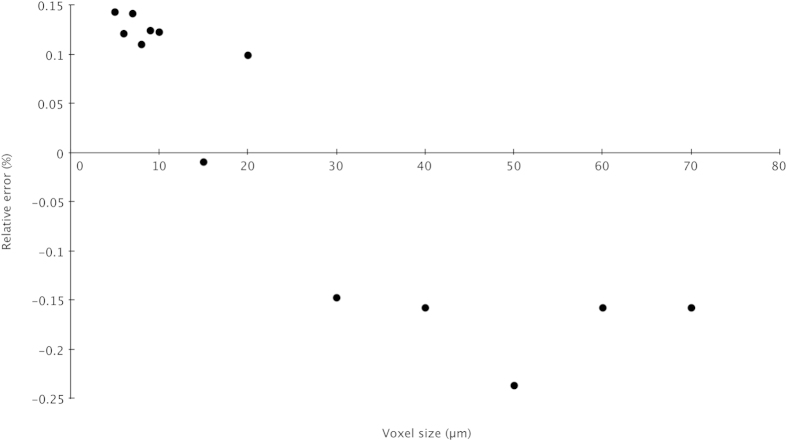
Voxel size versus relative error for Nikon, X-Tek XHT 225 ST scanner at Duke University (results comparable to Harvard’s X-Tek HMX 225 ST μCT scanner).

For measurement of the spheres, surfaces of all four (in both sets) were first generated, fit points (>10) were then placed on the surface and an idealized sphere was then fit to these points. Via use of the *Coordinate Measurement Module’s* sphere fitting function, diameters of each sphere were recorded, averaged and then compared to the physically measured diameter. The relative percentage error was calculated with the following equation: Relative error %=((average measurement of sphere diameter from VG—reported value of sphere from manufacturer)/(average measurement of sphere diameter from VG))*100. Table 2 gives data on relative errors. Figure 3 plots these error values against resolution.

These calibrations at different resolutions using spheres of known diameter suggest negligible error in absolute size or shape at the scanning resolutions between 5–70 microns for X-Tek microCT scanners (<0.2%). A comparison of digital and physical measurements of a skull from Duke’s collection, which was measured using calipers, scanned at 109 microns (cubic voxel dimension), and then measured digitally, indicate an error of <0.9%. The coarsest resolution used for the MCZ specimen scans is 125 microns, and while we do not have data on comparison between physical vouchers and digital avatars at this scale, we are confident that the potential for significant scale error is low.

## Usage Notes

### Working with the data

The data appear as series of 2D image files, with each image representing a cross section through the specimen. The file format is DICOM, which can be read by freeware ImageJ^5^, Avizo or Amira. ImageJ is predominantly useful for viewing the image cross sections. The whole series of files can be loaded into ImageJ by going to ‘File -> Import -> Image Sequence’. Often the contrast will look poor on initial opening, but this is merely a default issue. Go to ‘Image -> Adjust -> Brightness/Contrast’ and click ‘Auto’ to reset contrast or adjust the sliders to the desired image brightness and contrast. From ImageJ, the files can be re-saved as tiff format (16-bit) for modification or annotation in Adobe Photoshop, Illustrator or an equivalent program.

Three-dimensional visualization of these data is most easily done in Avizo or Amira. Two examples of freeware are Slicer3D or Fiji^6^. Other volume display and manipulation software include VG Studio MAX, Osirix, and Mimix.

### Computer requirements

In order to open a particular file in ImageJ your computer should have RAM exceeding the complete sample file size to some degree (the greater the better). As a rule of thumb, one should have twice the amount of RAM installed in his/her computer than the largest file size(s) one wishes to open. Otherwise not all of the images of the scan will open, and any processing will be extremely limited by the computers lack of space to manipulate the data. The minimum requirements for 3D visualization is similar, except more stringent. In this case, it is imperative that a computer is equipped with RAM equalling at least two times the file size for satisfactory processing and results with a 64-bit operating system (OS) installed—in many cases, a computer with at least 12 GB of RAM will suffice. Once this condition is met, the most important components are having a processor with high clock speed (>3 GHz), sufficient number of cores (≥2), and a higher-end graphics card (DDR5 with ≥2 GB).

### Citation of scans

Any publications using scans from this dataset should do the following: (1) list DOIs of all scans used; and (2) include the following statement in the acknowledgments: ‘Lynn Copes, Lynn Lucas, and the MCZ provided access to this [or these] scan[s], originally appearing in Copes and Kimbel^7^ and Copes *et al.* (2016), funding for the collection of which was provided by NSF DDIG #0925793, and a Wenner-Gren Foundation Dissertation Grant #8102 (both to Lynn Copes). These scans were downloaded from MorphoSource.org, a web-accessible archive for 3D digital data housed by Duke University.’

## Additional Information

**How to cite this article:** Copes, L. E. *et al.* A collection of non-human primate computed tomography scans housed in MorphoSource, a repository for 3D data. *Sci. Data* 3:160001 doi: 10.1038/sdata.2016.1 (2016).

## Supplementary Material



## Figures and Tables

**Table 1:** List of which elements of which specimens were scanned.

**Specimen**	**DOI**	**File size**	**Title**	**Taxonomy**	**X res**	**Y res**	**Z res**	**Voltage**	**Amperage**	**Watts**	**Projections**	**Copyright holder**	**Citation instruction statement (to be copy-pasted into acknolwedgements)**
MCZ-10131	doi:10.17602/M2/M4490	1.27 GB	MCZ 10131 skull	Saimiri oerstedii	0.047018 mm	0.047018 mm	0.047018 mm	80 kv	120 A	9.6 W	1050	MCZ - CC BY-NC	Citation: Lynn Lucas and Lynn Copes provided access to these data, the collection of which was funded by NSF DDIG #0925793 and Wenner Gren Foundation #8102. The files were downloaded from www.MorphoSource.org, Duke University.
MCZ-10132	doi:10.17602/M2/M4489	1.81 GB	MCZ 10132 skull	Saimiri oerstedii	0.047018 mm	0.047018 mm	0.047018 mm	80 kv	120 A	9.6 W	1050	MCZ - CC BY-NC	Citation: Lynn Lucas and Lynn Copes provided access to these data, the collection of which was funded by NSF DDIG #0925793 and Wenner Gren Foundation #8102. The files were downloaded from www.MorphoSource.org, Duke University.
MCZ-10133	doi:10.17602/M2/M4488	1.64 GB	MCZ 10133 skull	Saimiri oerstedii	0.047018 mm	0.047018 mm	0.047018 mm	80 kv	120 A	9.6 W	1050	MCZ - CC BY-NC	Citation: Lynn Lucas and Lynn Copes provided access to these data, the collection of which was funded by NSF DDIG #0925793 and Wenner Gren Foundation #8102. The files were downloaded from www.MorphoSource.org, Duke University.
MCZ-10134	doi:10.17602/M2/M4487	1.73 GB	MCZ 10134 skull	Saimiri oerstedii	0.047018 mm	0.047018 mm	0.047018 mm	80 kv	120 A	9.6 W	1050	MCZ - CC BY-NC	Citation: Lynn Lucas and Lynn Copes provided access to these data, the collection of which was funded by NSF DDIG #0925793 and Wenner Gren Foundation #8102. The files were downloaded from www.MorphoSource.org, Duke University.
MCZ-10135	doi:10.17602/M2/M5151	1.37 GB	MCZ 10135 skull	Cebus capucinus	0.079988 mm	0.079988 mm	0.079988 mm	80 kv	115 A	9.2 W	1100	MCZ - CC BY-NC	Citation: Lynn Lucas and Lynn Copes provided access to these data, the collection of which was funded by NSF DDIG #0925793 and Wenner Gren Foundation #8102. The files were downloaded from www.MorphoSource.org, Duke University.
MCZ-10136	doi:10.17602/M2/M5150	1.04 GB	MCZ 10136 skull	Cebus capucinus	0.079988 mm	0.079988 mm	0.079988 mm	80 kv	115 A	9.2 W	1100	MCZ - CC BY-NC	Citation: Lynn Lucas and Lynn Copes provided access to these data, the collection of which was funded by NSF DDIG #0925793 and Wenner Gren Foundation #8102. The files were downloaded from www.MorphoSource.org, Duke University.
MCZ-10138	doi:10.17602/M2/M2917	4.17 GB	MCZ 10138 skull	Ateles geoffroyi	0.063468 mm	0.063468 mm	0.063468 mm	90 kv	120 A	10.8 W	1100	MCZ - CC BY-NC	Citation: Lynn Lucas and Lynn Copes provided access to these data, the collection of which was funded by NSF DDIG #0925793 and Wenner Gren Foundation #8102. The files were downloaded from www.MorphoSource.org, Duke University.
MCZ-12729	doi:10.17602/M2/M4438	885.7 MB	MCZ 12729 humerus and femur	Trachypithecus cristata	0.096931 mm	0.096931 mm	0.096931 mm	90 kv	125 A	11.3 W	800	MCZ - CC BY-NC	Citation: Lynn Lucas and Lynn Copes provided access to these data, the collection of which was funded by NSF DDIG #0925793 and Wenner Gren Foundation #8102. The files were downloaded from www.MorphoSource.org, Duke University.
MCZ-12758	doi:10.17602/M2/M3028	4.61 GB	MCZ 12758 skull	Macaca fascicularis	0.061559 mm	0.061559 mm	0.061559 mm	80 kv	125 A	10 W	1050	MCZ - CC BY-NC	Citation: Lynn Lucas and Lynn Copes provided access to these data, the collection of which was funded by NSF DDIG #0925793 and Wenner Gren Foundation #8102. The files were downloaded from www.MorphoSource.org, Duke University.
MCZ-14657	doi:10.17602/M2/M2823	4.26 GB	MCZ 14657 skull	Euoticus elegantulus	0.026906 mm	0.026906 mm	0.026906 mm	80 kv	125 A	10 W	1000	MCZ - CC BY-NC	Citation: Lynn Lucas and Lynn Copes provided access to these data, the collection of which was funded by NSF DDIG #0925793 and Wenner Gren Foundation #8102. The files were downloaded from www.MorphoSource.org, Duke University.
MCZ-14658	doi:10.17602/M2/M2824	5.12 GB	MCZ 14658 skull	Euoticus elegantulus	0.026906 mm	0.026906 mm	0.026906 mm	80 kv	125 A	10 W	1000	MCZ - CC BY-NC	Citation: Lynn Lucas and Lynn Copes provided access to these data, the collection of which was funded by NSF DDIG #0925793 and Wenner Gren Foundation #8102. The files were downloaded from www.MorphoSource.org, Duke University.
MCZ-14659	doi:10.17602/M2/M2831	4.58 GB	MCZ 14659 skull	Galago alleni	0.027497 mm	0.027497 mm	0.027497 mm	80 kv	125 A	10 W	1000	MCZ - CC BY-NC	Citation: Lynn Lucas and Lynn Copes provided access to these data, the collection of which was funded by NSF DDIG #0925793 and Wenner Gren Foundation #8102. The files were downloaded from www.MorphoSource.org, Duke University.
MCZ-14725	doi:10.17602/M2/M2626	1.81 GB	MCZ 14725 skull	Cercocebus albigena	0.08971 mm	0.08971 mm	0.08971 mm	80 kv	125 A	10 W	1100	MCZ - CC BY-NC	Citation: Lynn Lucas and Lynn Copes provided access to these data, the collection of which was funded by NSF DDIG #0925793 and Wenner Gren Foundation #8102. The files were downloaded from www.MorphoSource.org, Duke University.
MCZ-14750	doi:10.17602/M2/M2938	2.94 GB	MCZ 14750 cranium	Gorilla gorilla gorilla	0.125974 mm	0.125974 mm	0.125974 mm	85 kv	90 A	7.7 W	1500	MCZ - CC BY-NC	Citation: Lynn Lucas and Lynn Copes provided access to these data, the collection of which was funded by NSF DDIG #0925793 and Wenner Gren Foundation #8102. The files were downloaded from www.MorphoSource.org, Duke University.
MCZ-14750	doi:10.17602/M2/M2940	4.01 GB	MCZ 14750 mandible	Gorilla gorilla gorilla	0.088816 mm	0.088816 mm	0.088816 mm	85 kv	90 A	7.7 W	1000	MCZ - CC BY-NC	Citation: Lynn Lucas and Lynn Copes provided access to these data, the collection of which was funded by NSF DDIG #0925793 and Wenner Gren Foundation #8102. The files were downloaded from www.MorphoSource.org, Duke University.
MCZ-15312	doi:10.17602/M2/M4391	3 GB	MCZ 15312 skull	Pan troglodytes troglodytes	0.105028 mm	0.105028 mm	0.105028 mm	80 kv	120 A	9.6 W	1500	MCZ - CC BY-NC	Citation: Lynn Lucas and Lynn Copes provided access to these data, the collection of which was funded by NSF DDIG #0925793 and Wenner Gren Foundation #8102. The files were downloaded from www.MorphoSource.org, Duke University.
MCZ-15324	doi:10.17602/M2/M4501	1.43 GB	MCZ 15324 skull	Saguinus sp.	0.040724 mm	0.040724 mm	0.040724 mm	80 kv	125 A	10 W	1000	MCZ - CC BY-NC	Citation: Lynn Lucas and Lynn Copes provided access to these data, the collection of which was funded by NSF DDIG #0925793 and Wenner Gren Foundation #8102. The files were downloaded from www.MorphoSource.org, Duke University.
MCZ-16075	doi:10.17602/M2/M2685	2.92 GB	MCZ 16075 skull	Galago senegalensis	0.025465 mm	0.025465 mm	0.025465 mm	80 kv	125 A	10 W	1000	MCZ - CC BY-NC	Citation: Lynn Lucas and Lynn Copes provided access to these data, the collection of which was funded by NSF DDIG #0925793 and Wenner Gren Foundation #8102. The files were downloaded from www.MorphoSource.org, Duke University.
MCZ-16354	doi:10.17602/M2/M2648	3.38 GB	MCZ 16354 skull	Eulemur fulvus fulvus	0.047648 mm	0.047648 mm	0.047648 mm	80 kv	125 A	10 W	1000	MCZ - CC BY-NC	Citation: Lynn Lucas and Lynn Copes provided access to these data, the collection of which was funded by NSF DDIG #0925793 and Wenner Gren Foundation #8102. The files were downloaded from www.MorphoSource.org, Duke University.
MCZ-16356	doi:10.17602/M2/M2652	2.84 GB	MCZ 16356 skull	Eulemur fulvus rufus	0.044297 mm	0.044297 mm	0.044297 mm	80 kv	125 A	10 W	1000	MCZ - CC BY-NC	Citation: Lynn Lucas and Lynn Copes provided access to these data, the collection of which was funded by NSF DDIG #0925793 and Wenner Gren Foundation #8102. The files were downloaded from www.MorphoSource.org, Duke University.
MCZ-16370	doi:10.17602/M2/M2655	3.8 GB	MCZ 16370 skull	Eulemur fulvus rufus	0.047648 mm	0.047648 mm	0.047648 mm	80 kv	125 A	10 W	1000	MCZ - CC BY-NC	Citation: Lynn Lucas and Lynn Copes provided access to these data, the collection of which was funded by NSF DDIG #0925793 and Wenner Gren Foundation #8102. The files were downloaded from www.MorphoSource.org, Duke University.
MCZ-16375	doi:10.17602/M2/M2724	3.51 GB	MCZ 16375 skull	Propithecus verreauxi verreauxi	0.048998 mm	0.048998 mm	0.048998 mm	80 kv	125 A	10 W	1000	MCZ - CC BY-NC	Citation: Lynn Lucas and Lynn Copes provided access to these data, the collection of which was funded by NSF DDIG #0925793 and Wenner Gren Foundation #8102. The files were downloaded from www.MorphoSource.org, Duke University.
MCZ-16382	doi:10.17602/M2/M2728	2.86 GB	MCZ 16382 skull	Varecia variegata variegata	0.054763 mm	0.054763 mm	0.054763 mm	80 kv	125 A	10 W	1000	MCZ - CC BY-NC	Citation: Lynn Lucas and Lynn Copes provided access to these data, the collection of which was funded by NSF DDIG #0925793 and Wenner Gren Foundation #8102. The files were downloaded from www.MorphoSource.org, Duke University.
MCZ-16390	doi:10.17602/M2/M4503	3.41 GB	MCZ 16390 skull	Propithecus diadema	0.048998 mm	0.048998 mm	0.048998 mm	80 kv	125 A	10 W	1000	MCZ - CC BY-NC	Citation: Lynn Lucas and Lynn Copes provided access to these data, the collection of which was funded by NSF DDIG #0925793 and Wenner Gren Foundation #8102. The files were downloaded from www.MorphoSource.org, Duke University.
MCZ-16391	doi:10.17602/M2/M2839	2.62 GB	MCZ 16391 skull	Lemur catta	0.044601 mm	0.044601 mm	0.044601 mm	80 kv	125 A	10 W	1000	MCZ - CC BY-NC	Citation: Lynn Lucas and Lynn Copes provided access to these data, the collection of which was funded by NSF DDIG #0925793 and Wenner Gren Foundation #8102. The files were downloaded from www.MorphoSource.org, Duke University.
MCZ-16392	doi:10.17602/M2/M2840	3.05 GB	MCZ 16392 skull	Lemur catta	0.044601 mm	0.044601 mm	0.044601 mm	80 kv	125 A	10 W	1000	MCZ - CC BY-NC	Citation: Lynn Lucas and Lynn Copes provided access to these data, the collection of which was funded by NSF DDIG #0925793 and Wenner Gren Foundation #8102. The files were downloaded from www.MorphoSource.org, Duke University.
MCZ-16393	doi:10.17602/M2/M2658	4.34 GB	MCZ 16393 skull	Eulemur fulvus rufus	0.045992 mm	0.045992 mm	0.045992 mm	80 kv	125 A	10 W	1000	MCZ - CC BY-NC	Citation: Lynn Lucas and Lynn Copes provided access to these data, the collection of which was funded by NSF DDIG #0925793 and Wenner Gren Foundation #8102. The files were downloaded from www.MorphoSource.org, Duke University.
MCZ-17342	doi:10.17602/M2/M4885	2.73 GB	MCZ 17342 cranium	Papio doguera	0.107695 mm	0.107695 mm	0.107695 mm	85 kv	125 A	10 W	1100	MCZ - CC BY-NC	Citation: Lynn Lucas and Lynn Copes provided access to these data, the collection of which was funded by NSF DDIG #0925793 and Wenner Gren Foundation #8102. The files were downloaded from www.MorphoSource.org, Duke University.
MCZ-17342	doi:10.17602/M2/M4886	2.6 GB	MCZ 17342 mandible	Papio doguera	0.082975 mm	0.082975 mm	0.082975 mm	85 kv	125 A	10 W	1100	MCZ - CC BY-NC	Citation: Lynn Lucas and Lynn Copes provided access to these data, the collection of which was funded by NSF DDIG #0925793 and Wenner Gren Foundation #8102. The files were downloaded from www.MorphoSource.org, Duke University.
MCZ-17343	doi:10.17602/M2/M4883	1.8 GB	MCZ 17343 cranium	Papio doguera	0.125607 mm	0.125607 mm	0.125607 mm	90 kv	125 A	11.3 W	1100	MCZ - CC BY-NC	Citation: Lynn Lucas and Lynn Copes provided access to these data, the collection of which was funded by NSF DDIG #0925793 and Wenner Gren Foundation #8102. The files were downloaded from www.MorphoSource.org, Duke University.
MCZ-17343	doi:10.17602/M2/M4884	2.72 GB	MCZ 17343 mandible	Papio doguera	0.090033 mm	0.090033 mm	0.090033 mm	90 kv	125 A	11.3 W	1100	MCZ - CC BY-NC	Citation: Lynn Lucas and Lynn Copes provided access to these data, the collection of which was funded by NSF DDIG #0925793 and Wenner Gren Foundation #8102. The files were downloaded from www.MorphoSource.org, Duke University.
MCZ-17548	doi:10.17602/M2/M4720	4.46 GB	MCZ 17548 skull	Perodicticus potto	0.03837 mm	0.03837 mm	0.03837 mm	80 kv	125 A	10 W	1000	MCZ - CC BY-NC	Citation: Lynn Lucas and Lynn Copes provided access to these data, the collection of which was funded by NSF DDIG #0925793 and Wenner Gren Foundation #8102. The files were downloaded from www.MorphoSource.org, Duke University.
MCZ-17550	doi:10.17602/M2/M4719	4.75 GB	MCZ 17550 skull	Perodicticus potto	0.039518 mm	0.039518 mm	0.039518 mm	80 kv	125 A	10 W	1000	MCZ - CC BY-NC	Citation: Lynn Lucas and Lynn Copes provided access to these data, the collection of which was funded by NSF DDIG #0925793 and Wenner Gren Foundation #8102. The files were downloaded from www.MorphoSource.org, Duke University.
MCZ-17589	doi:10.17602/M2/M2832	4.64 GB	MCZ 17589 skull	Galago alleni	0.027497 mm	0.027497 mm	0.027497 mm	80 kv	125 A	10 W	1000	MCZ - CC BY-NC	Citation: Lynn Lucas and Lynn Copes provided access to these data, the collection of which was funded by NSF DDIG #0925793 and Wenner Gren Foundation #8102. The files were downloaded from www.MorphoSource.org, Duke University.
MCZ-17590	doi:10.17602/M2/M2825	5.54 GB	MCZ 17590 skull	Euoticus elegantulus	0.026906 mm	0.026906 mm	0.026906 mm	80 kv	125 A	10 W	1000	MCZ - CC BY-NC	Citation: Lynn Lucas and Lynn Copes provided access to these data, the collection of which was funded by NSF DDIG #0925793 and Wenner Gren Foundation #8102. The files were downloaded from www.MorphoSource.org, Duke University.
MCZ-17591	doi:10.17602/M2/M2826	4.76 GB	MCZ 17591 skull	Euoticus elegantulus	0.026906 mm	0.026906 mm	0.026906 mm	80 kv	125 A	10 W	1000	MCZ - CC BY-NC	Citation: Lynn Lucas and Lynn Copes provided access to these data, the collection of which was funded by NSF DDIG #0925793 and Wenner Gren Foundation #8102. The files were downloaded from www.MorphoSource.org, Duke University.
MCZ-17592	doi:10.17602/M2/M2827	2.02 GB	MCZ 17592 skull	Euoticus elegantulus	0.026906 mm	0.026906 mm	0.026906 mm	80 kv	125 A	10 W	1000	MCZ - CC BY-NC	Citation: Lynn Lucas and Lynn Copes provided access to these data, the collection of which was funded by NSF DDIG #0925793 and Wenner Gren Foundation #8102. The files were downloaded from www.MorphoSource.org, Duke University.
MCZ-17593	doi:10.17602/M2/M2828	4.83 GB	MCZ 17593 skull	Euoticus elegantulus	0.026906 mm	0.026906 mm	0.026906 mm	80 kv	125 A	10 W	1000	MCZ - CC BY-NC	Citation: Lynn Lucas and Lynn Copes provided access to these data, the collection of which was funded by NSF DDIG #0925793 and Wenner Gren Foundation #8102. The files were downloaded from www.MorphoSource.org, Duke University.
MCZ-17684	doi:10.17602/M2/M2943	2.93 GB	MCZ 17684 cranium	Gorilla gorilla gorilla	0.125974 mm	0.125974 mm	0.125974 mm	85 kv	90 A	7.7 W	1500	MCZ - CC BY-NC	Citation: Lynn Lucas and Lynn Copes provided access to these data, the collection of which was funded by NSF DDIG #0925793 and Wenner Gren Foundation #8102. The files were downloaded from www.MorphoSource.org, Duke University.
MCZ-17684	doi:10.17602/M2/M2944	6.23 GB	MCZ 17684 mandible	Gorilla gorilla gorilla	0.08136 mm	0.08136 mm	0.08136 mm	85 kv	90 A	7.7 W	1000	MCZ - CC BY-NC	Citation: Lynn Lucas and Lynn Copes provided access to these data, the collection of which was funded by NSF DDIG #0925793 and Wenner Gren Foundation #8102. The files were downloaded from www.MorphoSource.org, Duke University.
MCZ-17702	doi:10.17602/M2/M4395	3.02 GB	MCZ 17702 cranium	Pan troglodytes	0.102401 mm	0.102401 mm	0.102401 mm	80 kv	120 A	9.6 W	1500	MCZ - CC BY-NC	Citation: Lynn Lucas and Lynn Copes provided access to these data, the collection of which was funded by NSF DDIG #0925793 and Wenner Gren Foundation #8102. The files were downloaded from www.MorphoSource.org, Duke University.
MCZ-17702	doi:10.17602/M2/M4396	4.16 GB	MCZ 17702 mandible	Pan troglodytes	0.07725 mm	0.07725 mm	0.07725 mm	80 kv	120 A	9.6 W	1000	MCZ - CC BY-NC	Citation: Lynn Lucas and Lynn Copes provided access to these data, the collection of which was funded by NSF DDIG #0925793 and Wenner Gren Foundation #8102. The files were downloaded from www.MorphoSource.org, Duke University.
MCZ-18607	doi:10.17602/M2/M4718	4.13 GB	MCZ 18607 skull	Perodicticus potto	0.028886 mm	0.028886 mm	0.028886 mm	80 kv	125 A	10 W	1000	MCZ - CC BY-NC	Citation: Lynn Lucas and Lynn Copes provided access to these data, the collection of which was funded by NSF DDIG #0925793 and Wenner Gren Foundation #8102. The files were downloaded from www.MorphoSource.org, Duke University.
MCZ-18608	doi:10.17602/M2/M2829	5.45 GB	MCZ 18608 skull	Euoticus elegantulus	0.026906 mm	0.026906 mm	0.026906 mm	80 kv	125 A	10 W	1000	MCZ - CC BY-NC	Citation: Lynn Lucas and Lynn Copes provided access to these data, the collection of which was funded by NSF DDIG #0925793 and Wenner Gren Foundation #8102. The files were downloaded from www.MorphoSource.org, Duke University.
MCZ-18609	doi:10.17602/M2/M2835	4.41 GB	MCZ 18609 skull	Euoticus elegantulus	0.026906 mm	0.026906 mm	0.026906 mm	80 kv	125 A	10 W	1000	MCZ - CC BY-NC	Citation: Lynn Lucas and Lynn Copes provided access to these data, the collection of which was funded by NSF DDIG #0925793 and Wenner Gren Foundation #8102. The files were downloaded from www.MorphoSource.org, Duke University.
MCZ-18612	doi:10.17602/M2/M2599	2.18 GB	MCZ 18612 skull	Cercocebus torquatus	0.090751 mm	0.090751 mm	0.090751 mm	85 kv	126 A	10.6 W	1100	MCZ - CC BY-NC	Citation: Lynn Lucas and Lynn Copes provided access to these data, the collection of which was funded by NSF DDIG #0925793 and Wenner Gren Foundation #8102. The files were downloaded from www.MorphoSource.org, Duke University.
MCZ-18614	doi:10.17602/M2/M2622	1.88 GB	MCZ 18614 skull	Cercocebus albigena	0.08971 mm	0.08971 mm	0.08971 mm	80 kv	125 A	10 W	1100	MCZ - CC BY-NC	Citation: Lynn Lucas and Lynn Copes provided access to these data, the collection of which was funded by NSF DDIG #0925793 and Wenner Gren Foundation #8102. The files were downloaded from www.MorphoSource.org, Duke University.
MCZ-18631	doi:10.17602/M2/M2725	3.48 GB	MCZ 18631 skull	Propithecus verreauxi deckenii	0.048998 mm	0.048998 mm	0.048998 mm	80 kv	125 A	10 W	1000	MCZ - CC BY-NC	Citation: Lynn Lucas and Lynn Copes provided access to these data, the collection of which was funded by NSF DDIG #0925793 and Wenner Gren Foundation #8102. The files were downloaded from www.MorphoSource.org, Duke University.
MCZ-18740	doi:10.17602/M2/M4402	3.77 MB	MCZ 18740 skull	Varecia variegata	0.054763 mm	0.054763 mm	0.054763 mm	80 kv	125 A	10 W	1000	MCZ - CC BY-NC	Citation: Lynn Lucas and Lynn Copes provided access to these data, the collection of which was funded by NSF DDIG #0925793 and Wenner Gren Foundation #8102. The files were downloaded from www.MorphoSource.org, Duke University.
MCZ-19184	doi:10.17602/M2/M2598	3.05 GB	MCZ 19184 skull	Cercocebus torquatus	0.090751 mm	0.090751 mm	0.090751 mm	85 kv	125 A	10.6 W	1100	MCZ - CC BY-NC	Citation: Lynn Lucas and Lynn Copes provided access to these data, the collection of which was funded by NSF DDIG #0925793 and Wenner Gren Foundation #8102. The files were downloaded from www.MorphoSource.org, Duke University.
MCZ-1957	doi:10.17602/M2/M5156	1.59 GB	MCZ 1957 skull	Cacajao calvus rubicundus	0.079988 mm	0.079988 mm	0.079988 mm	80 kv	115 A	9.2 W	1100	MCZ - CC BY-NC	Citation: Lynn Lucas and Lynn Copes provided access to these data, the collection of which was funded by NSF DDIG #0925793 and Wenner Gren Foundation #8102. The files were downloaded from www.MorphoSource.org, Duke University.
MCZ-19801	doi:10.17602/M2/M2870	3.11 GB	MCZ 19801 skull	Aotus trivirgatus	0.040724 mm	0.040724 mm	0.040724 mm	80 kv	125 A	10 W	1000	MCZ - CC BY-NC	Citation: Lynn Lucas and Lynn Copes provided access to these data, the collection of which was funded by NSF DDIG #0925793 and Wenner Gren Foundation #8102. The files were downloaded from www.MorphoSource.org, Duke University.
MCZ-19802	doi:10.17602/M2/M2871	3.24 GB	MCZ 19802 skull	Aotus trivirgatus	0.040724 mm	0.040724 mm	0.040724 mm	80 kv	125 A	10 W	1000	MCZ - CC BY-NC	Citation: Lynn Lucas and Lynn Copes provided access to these data, the collection of which was funded by NSF DDIG #0925793 and Wenner Gren Foundation #8102. The files were downloaded from www.MorphoSource.org, Duke University.
MCZ-19805	doi:10.17602/M2/M2872	3 GB	MCZ 19805 skull	Aotus trivirgatus	0.040724 mm	0.040724 mm	0.040724 mm	80 kv	125 A	10 W	1000	MCZ - CC BY-NC	Citation: Lynn Lucas and Lynn Copes provided access to these data, the collection of which was funded by NSF DDIG #0925793 and Wenner Gren Foundation #8102. The files were downloaded from www.MorphoSource.org, Duke University.
MCZ-19969	doi:10.17602/M2/M2833	3.37 GB	MCZ 19969 skull	Galago alleni	0.04766 mm	0.04766 mm	0.04766 mm	80 kv	125 A	10 W	1000	MCZ - CC BY-NC	Citation: Lynn Lucas and Lynn Copes provided access to these data, the collection of which was funded by NSF DDIG #0925793 and Wenner Gren Foundation #8102. The files were downloaded from www.MorphoSource.org, Duke University.
MCZ-19976	doi:10.17602/M2/M5095	2.39 GB	MCZ 19976 skull	Miopithecus talapoin	0.050092 mm	0.050092 mm	0.050092 mm	80 kv	125 A	10 W	1000	MCZ - CC BY-NC	Citation: Lynn Lucas and Lynn Copes provided access to these data, the collection of which was funded by NSF DDIG #0925793 and Wenner Gren Foundation #8102. The files were downloaded from www.MorphoSource.org, Duke University.
MCZ-19982	doi:10.17602/M2/M2597	3.32 GB	MCZ 19982 skull	Cercocebus torquatus	0.092963 mm	0.092963 mm	0.092963 mm	85 kv	125 A	10.6 W	1100	MCZ - CC BY-NC	Citation: Lynn Lucas and Lynn Copes provided access to these data, the collection of which was funded by NSF DDIG #0925793 and Wenner Gren Foundation #8102. The files were downloaded from www.MorphoSource.org, Duke University.
MCZ-19986	doi:10.17602/M2/M3059	2.63 GB	MCZ 19986 cranium	Mandrillus leucophaeus	0.111129 mm	0.111129 mm	0.111129 mm	85 kv	125 A	10 W	1100	MCZ - CC BY-NC	Citation: Lynn Lucas and Lynn Copes provided access to these data, the collection of which was funded by NSF DDIG #0925793 and Wenner Gren Foundation #8102. The files were downloaded from www.MorphoSource.org, Duke University.
MCZ-19986	doi:10.17602/M2/M3060	2.46 GB	MCZ 19986 mandible	Mandrillus leucophaeus	0.090718 mm	0.090718 mm	0.090718 mm	85 kv	125 A	10 W	1100	MCZ - CC BY-NC	Citation: Lynn Lucas and Lynn Copes provided access to these data, the collection of which was funded by NSF DDIG #0925793 and Wenner Gren Foundation #8102. The files were downloaded from www.MorphoSource.org, Duke University.
MCZ-20085	doi:10.17602/M2/M3062	2.54 GB	MCZ 20085 cranium	Mandrillus leucophaeus	0.111129 mm	0.111129 mm	0.111129 mm	85 kv	125 A	10 W	1100	MCZ - CC BY-NC	Citation: Lynn Lucas and Lynn Copes provided access to these data, the collection of which was funded by NSF DDIG #0925793 and Wenner Gren Foundation #8102. The files were downloaded from www.MorphoSource.org, Duke University.
MCZ-20085	doi:10.17602/M2/M3063	3.74 GB	MCZ 20085 mandible	Mandrillus leucophaeus	0.07842 mm	0.07842 mm	0.07842 mm	85 kv	125 A	10 W	1100	MCZ - CC BY-NC	Citation: Lynn Lucas and Lynn Copes provided access to these data, the collection of which was funded by NSF DDIG #0925793 and Wenner Gren Foundation #8102. The files were downloaded from www.MorphoSource.org, Duke University.
MCZ-20089	doi:10.17602/M2/M2945	2.23 GB	MCZ 20089 cranium	Gorilla gorilla gorilla	0.118952 mm	0.118952 mm	0.118952 mm	85 kv	90 A	7.7 W	1500	MCZ - CC BY-NC	Citation: Lynn Lucas and Lynn Copes provided access to these data, the collection of which was funded by NSF DDIG #0925793 and Wenner Gren Foundation #8102. The files were downloaded from www.MorphoSource.org, Duke University.
MCZ-20089	doi:10.17602/M2/M2946	2.7 GB	MCZ 20089 mandible	Gorilla gorilla gorilla	0.083214 mm	0.083214 mm	0.083214 mm	85 kv	90 A	7.7 W	1000	MCZ - CC BY-NC	Citation: Lynn Lucas and Lynn Copes provided access to these data, the collection of which was funded by NSF DDIG #0925793 and Wenner Gren Foundation #8102. The files were downloaded from www.MorphoSource.org, Duke University.
MCZ-20099	doi:10.17602/M2/M2873	3.23 GB	MCZ 20099 skull	Aotus trivirgatus	0.040724 mm	0.040724 mm	0.040724 mm	80 kv	125 A	10 W	1000	MCZ - CC BY-NC	Citation: Lynn Lucas and Lynn Copes provided access to these data, the collection of which was funded by NSF DDIG #0925793 and Wenner Gren Foundation #8102. The files were downloaded from www.MorphoSource.org, Duke University.
MCZ-20186	doi:10.17602/M2/M5158	1.99 GB	MCZ 20186 skull	Callicebus moloch	0.047097 mm	0.047097 mm	0.047097 mm	80 kv	120 A	9.6 W	1050	MCZ - CC BY-NC	Citation: Lynn Lucas and Lynn Copes provided access to these data, the collection of which was funded by NSF DDIG #0925793 and Wenner Gren Foundation #8102. The files were downloaded from www.MorphoSource.org, Duke University.
MCZ-20187	doi:10.17602/M2/M4486	1.93 GB	MCZ 20187 skull	Saimiri sciureus	0.047018 mm	0.047018 mm	0.047018 mm	80 kv	120 A	9.6 W	1050	MCZ - CC BY-NC	Citation: Lynn Lucas and Lynn Copes provided access to these data, the collection of which was funded by NSF DDIG #0925793 and Wenner Gren Foundation #8102. The files were downloaded from www.MorphoSource.org, Duke University.
MCZ-20188	doi:10.17602/M2/M5159	1.66 GB	MCZ 20188 skull	Callicebus moloch	0.047097 mm	0.047097 mm	0.047097 mm	80 kv	120 A	9.6 W	1050	MCZ - CC BY-NC	Citation: Lynn Lucas and Lynn Copes provided access to these data, the collection of which was funded by NSF DDIG #0925793 and Wenner Gren Foundation #8102. The files were downloaded from www.MorphoSource.org, Duke University.
MCZ-20265	doi:10.17602/M2/M4710	3.33 GB	MCZ 20265 skull	Pithecia monachus	0.050092 mm	0.050092 mm	0.050092 mm	80 kv	115 A	9.2 W	1050	MCZ - CC BY-NC	Citation: Lynn Lucas and Lynn Copes provided access to these data, the collection of which was funded by NSF DDIG #0925793 and Wenner Gren Foundation #8102. The files were downloaded from www.MorphoSource.org, Duke University.
MCZ-20266	doi:10.17602/M2/M4709	2.64 GB	MCZ 20266 skull	Pithecia monachus	0.050142 mm	0.050142 mm	0.050142 mm	80 kv	115 A	9.2 W	1050	MCZ - CC BY-NC	Citation: Lynn Lucas and Lynn Copes provided access to these data, the collection of which was funded by NSF DDIG #0925793 and Wenner Gren Foundation #8102. The files were downloaded from www.MorphoSource.org, Duke University.
MCZ-21147	doi:10.17602/M2/M2903	2.71 GB	MCZ 21147 skull	Colobus polykomos	0.08006 mm	0.08006 mm	0.08006 mm	70 kv	110 A	7.7 W	1050	MCZ - CC BY-NC	Citation: Lynn Lucas and Lynn Copes provided access to these data, the collection of which was funded by NSF DDIG #0925793 and Wenner Gren Foundation #8102. The files were downloaded from www.MorphoSource.org, Duke University.
MCZ-21151	doi:10.17602/M2/M2904	2.81 GB	MCZ 21151 skull	Colobus polykomos	0.07602 mm	0.07602 mm	0.07602 mm	70 kv	110 A	7.7 W	1050	MCZ - CC BY-NC	Citation: Lynn Lucas and Lynn Copes provided access to these data, the collection of which was funded by NSF DDIG #0925793 and Wenner Gren Foundation #8102. The files were downloaded from www.MorphoSource.org, Duke University.
MCZ-21153	doi:10.17602/M2/M2905	3.21 GB	MCZ 21153 skull	Colobus polykomos	0.07602 mm	0.07602 mm	0.07602 mm	70 kv	110 A	7.7 W	1050	MCZ - CC BY-NC	Citation: Lynn Lucas and Lynn Copes provided access to these data, the collection of which was funded by NSF DDIG #0925793 and Wenner Gren Foundation #8102. The files were downloaded from www.MorphoSource.org, Duke University.
MCZ-21155	doi:10.17602/M2/M2601	2.29 GB	MCZ 21155 skull	Cercocebus sp.	0.092963 mm	0.092963 mm	0.092963 mm	85 kv	125 A	10.6 W	1100	MCZ - CC BY-NC	Citation: Lynn Lucas and Lynn Copes provided access to these data, the collection of which was funded by NSF DDIG #0925793 and Wenner Gren Foundation #8102. The files were downloaded from www.MorphoSource.org, Duke University.
MCZ-21160	doi:10.17602/M2/M4881	2.98 GB	MCZ 21160 cranium	Papio doguera	0.117627 mm	0.117627 mm	0.117627 mm	85 kv	125 A	10 W	1100	MCZ - CC BY-NC	Citation: Lynn Lucas and Lynn Copes provided access to these data, the collection of which was funded by NSF DDIG #0925793 and Wenner Gren Foundation #8102. The files were downloaded from www.MorphoSource.org, Duke University.
MCZ-21160	doi:10.17602/M2/M4882	4.38 GB	MCZ 21160 mandible	Papio doguera	0.082975 mm	0.082975 mm	0.082975 mm	85 kv	125 A	10 W	1100	MCZ - CC BY-NC	Citation: Lynn Lucas and Lynn Copes provided access to these data, the collection of which was funded by NSF DDIG #0925793 and Wenner Gren Foundation #8102. The files were downloaded from www.MorphoSource.org, Duke University.
MCZ-21161	doi:10.17602/M2/M4879	2.2 GB	MCZ 21161 cranium	Papio doguera	0.117658 mm	0.117658 mm	0.117658 mm	90 kv	125 A	11.3 W	1100	MCZ - CC BY-NC	Citation: Lynn Lucas and Lynn Copes provided access to these data, the collection of which was funded by NSF DDIG #0925793 and Wenner Gren Foundation #8102. The files were downloaded from www.MorphoSource.org, Duke University.
MCZ-21161	doi:10.17602/M2/M4880	3.88 GB	MCZ 21161 mandible	Papio doguera	0.082975 mm	0.082975 mm	0.082975 mm	90 kv	125 A	11.3 W	1100	MCZ - CC BY-NC	Citation: Lynn Lucas and Lynn Copes provided access to these data, the collection of which was funded by NSF DDIG #0925793 and Wenner Gren Foundation #8102. The files were downloaded from www.MorphoSource.org, Duke University.
MCZ-22276	doi:10.17602/M2/M4557	2 GB	MCZ 22276 skull	Presbytis rubicunda	0.079988 mm	0.079988 mm	0.079988 mm	80 kv	120 A	9.6 W	1100	MCZ - CC BY-NC	Citation: Lynn Lucas and Lynn Copes provided access to these data, the collection of which was funded by NSF DDIG #0925793 and Wenner Gren Foundation #8102. The files were downloaded from www.MorphoSource.org, Duke University.
MCZ-22277	doi:10.17602/M2/M3029	3.88 GB	MCZ 22277 skull	Macaca fascicularis	0.061559 mm	0.061559 mm	0.061559 mm	80 kv	125 A	10 W	1050	MCZ - CC BY-NC	Citation: Lynn Lucas and Lynn Copes provided access to these data, the collection of which was funded by NSF DDIG #0925793 and Wenner Gren Foundation #8102. The files were downloaded from www.MorphoSource.org, Duke University.
MCZ-22356	doi:10.17602/M2/M2906	2.27 GB	MCZ 22356 skull	Colobus polykomos	0.07602 mm	0.07602 mm	0.07602 mm	70 kv	110 A	7.7 W	1050	MCZ - CC BY-NC	Citation: Lynn Lucas and Lynn Copes provided access to these data, the collection of which was funded by NSF DDIG #0925793 and Wenner Gren Foundation #8102. The files were downloaded from www.MorphoSource.org, Duke University.
MCZ-22358	doi:10.17602/M2/M2907	2.93 GB	MCZ 22358 skull	Colobus polykomos	0.07602 mm	0.07602 mm	0.07602 mm	85 kv	125 A	10.6 W	1050	MCZ - CC BY-NC	Citation: Lynn Lucas and Lynn Copes provided access to these data, the collection of which was funded by NSF DDIG #0925793 and Wenner Gren Foundation #8102. The files were downloaded from www.MorphoSource.org, Duke University.
MCZ-22624	doi:10.17602/M2/M2908	3.03 GB	MCZ 22624 skull	Colobus polykomos	0.07602 mm	0.07602 mm	0.07602 mm	85 kv	125 A	10.6 W	1050	MCZ - CC BY-NC	Citation: Lynn Lucas and Lynn Copes provided access to these data, the collection of which was funded by NSF DDIG #0925793 and Wenner Gren Foundation #8102. The files were downloaded from www.MorphoSource.org, Duke University.
MCZ-22626	doi:10.17602/M2/M2909	2.29 GB	MCZ 22626 skull	Colobus polykomos	0.07602 mm	0.07602 mm	0.07602 mm	85 kv	125 A	10.6 W	1050	MCZ - CC BY-NC	Citation: Lynn Lucas and Lynn Copes provided access to these data, the collection of which was funded by NSF DDIG #0925793 and Wenner Gren Foundation #8102. The files were downloaded from www.MorphoSource.org, Duke University.
MCZ-22629	doi:10.17602/M2/M2910	2.86 GB	MCZ 22629 skull	Colobus polykomos	0.07602 mm	0.07602 mm	0.07602 mm	85 kv	125 A	10.6 W	1050	MCZ - CC BY-NC	Citation: Lynn Lucas and Lynn Copes provided access to these data, the collection of which was funded by NSF DDIG #0925793 and Wenner Gren Foundation #8102. The files were downloaded from www.MorphoSource.org, Duke University.
MCZ-22734	doi:10.17602/M2/M2928	1.12 GB	MCZ 22734 skull	Cercopithecus mitis	0.079988 mm	0.079988 mm	0.079988 mm	80 kv	125 A	10 W	1100	MCZ - CC BY-NC	Citation: Lynn Lucas and Lynn Copes provided access to these data, the collection of which was funded by NSF DDIG #0925793 and Wenner Gren Foundation #8102. The files were downloaded from www.MorphoSource.org, Duke University.
MCZ-22736	doi:10.17602/M2/M2732	1.6 GB	MCZ 22736 skull	Cercocebus albigena	0.08971 mm	0.08971 mm	0.08971 mm	80 kv	125 A	10 W	1000	MCZ - CC BY-NC	Citation: Lynn Lucas and Lynn Copes provided access to these data, the collection of which was funded by NSF DDIG #0925793 and Wenner Gren Foundation #8102. The files were downloaded from www.MorphoSource.org, Duke University.
MCZ-22737	doi:10.17602/M2/M2621	1.94 GB	MCZ 22737 skull	Cercocebus albigena	0.08971 mm	0.08971 mm	0.08971 mm	80 kv	125 A	10.6 W	1100	MCZ - CC BY-NC	Citation: Lynn Lucas and Lynn Copes provided access to these data, the collection of which was funded by NSF DDIG #0925793 and Wenner Gren Foundation #8102. The files were downloaded from www.MorphoSource.org, Duke University.
MCZ-22850	doi:10.17602/M2/M2911	2.99 GB	MCZ 22850 skull	Colobus polykomos	0.07602 mm	0.07602 mm	0.07602 mm	80 kv	125 A	8.4 W	1050	MCZ - CC BY-NC	Citation: Lynn Lucas and Lynn Copes provided access to these data, the collection of which was funded by NSF DDIG #0925793 and Wenner Gren Foundation #8102. The files were downloaded from www.MorphoSource.org, Duke University.
MCZ-23167	doi:10.17602/M2/M4390	3.29 GB	MCZ 23167 skull	Pan troglodytes	0.106004 mm	0.106004 mm	0.106004 mm	80 kv	120 A	9.6 W	1500	MCZ - CC BY-NC	Citation: Lynn Lucas and Lynn Copes provided access to these data, the collection of which was funded by NSF DDIG #0925793 and Wenner Gren Foundation #8102. The files were downloaded from www.MorphoSource.org, Duke University.
MCZ-23168	doi:10.17602/M2/M3066	3.37 GB	MCZ 23168 cranium	Mandrillus leucophaeus	0.111129 mm	0.111129 mm	0.111129 mm	85 kv	125 A	10 W	1100	MCZ - CC BY-NC	Citation: Lynn Lucas and Lynn Copes provided access to these data, the collection of which was funded by NSF DDIG #0925793 and Wenner Gren Foundation #8102. The files were downloaded from www.MorphoSource.org, Duke University.
MCZ-23168	doi:10.17602/M2/M3074	3.38 GB	MCZ 23168 mandible	Mandrillus leucophaeus	0.090717 mm	0.090717 mm	0.090717 mm	85 kv	125 A	10 W	1100	MCZ - CC BY-NC	Citation: Lynn Lucas and Lynn Copes provided access to these data, the collection of which was funded by NSF DDIG #0925793 and Wenner Gren Foundation #8102. The files were downloaded from www.MorphoSource.org, Duke University.
MCZ-23169	doi:10.17602/M2/M3081	2.33 GB	MCZ 23169 cranium	Mandrillus leucophaeus	0.111129 mm	0.111129 mm	0.111129 mm	85 kv	125 A	10 W	1100	MCZ - CC BY-NC	Citation: Lynn Lucas and Lynn Copes provided access to these data, the collection of which was funded by NSF DDIG #0925793 and Wenner Gren Foundation #8102. The files were downloaded from www.MorphoSource.org, Duke University.
MCZ-23169	doi:10.17602/M2/M3100	4.09 GB	MCZ 23169 mandible	Mandrillus leucophaeus	0.077196 mm	0.077196 mm	0.077196 mm	85 kv	125 A	10 W	1100	MCZ - CC BY-NC	Citation: Lynn Lucas and Lynn Copes provided access to these data, the collection of which was funded by NSF DDIG #0925793 and Wenner Gren Foundation #8102. The files were downloaded from www.MorphoSource.org, Duke University.
MCZ-23194	doi:10.17602/M2/M2617	1.79 GB	MCZ 23194 skull	Cercocebus albigena	0.08971 mm	0.08971 mm	0.08971 mm	80 kv	125 A	10 W	1100	MCZ - CC BY-NC	Citation: Lynn Lucas and Lynn Copes provided access to these data, the collection of which was funded by NSF DDIG #0925793 and Wenner Gren Foundation #8102. The files were downloaded from www.MorphoSource.org, Duke University.
MCZ-23195	doi:10.17602/M2/M2596	1.83 GB	MCZ 23195 skull	Cercocebus sp.	0.092963 mm	0.092963 mm	0.092963 mm	85 kv	125 A	10.6 W	1100	MCZ - CC BY-NC	Citation: Lynn Lucas and Lynn Copes provided access to these data, the collection of which was funded by NSF DDIG #0925793 and Wenner Gren Foundation #8102. The files were downloaded from www.MorphoSource.org, Duke University.
MCZ-23196	doi:10.17602/M2/M5094	3.4 GB	MCZ 23196 skull	Miopithecus talapoin	0.050092 mm	0.050092 mm	0.050092 mm	80 kv	125 A	10 W	1000	MCZ - CC BY-NC	Citation: Lynn Lucas and Lynn Copes provided access to these data, the collection of which was funded by NSF DDIG #0925793 and Wenner Gren Foundation #8102. The files were downloaded from www.MorphoSource.org, Duke University.
MCZ-23197	doi:10.17602/M2/M5093	2.56 GB	MCZ 23197 skull	Miopithecus talapoin	0.050092 mm	0.050092 mm	0.050092 mm	80 kv	125 A	10 W	1000	MCZ - CC BY-NC	Citation: Lynn Lucas and Lynn Copes provided access to these data, the collection of which was funded by NSF DDIG #0925793 and Wenner Gren Foundation #8102. The files were downloaded from www.MorphoSource.org, Duke University.
MCZ-23812	doi:10.17602/M2/M3030	3.43 GB	MCZ 23812 skull	Macaca fascicularis	0.061559 mm	0.061559 mm	0.061559 mm	80 kv	125 A	10 W	1050	MCZ - CC BY-NC	Citation: Lynn Lucas and Lynn Copes provided access to these data, the collection of which was funded by NSF DDIG #0925793 and Wenner Gren Foundation #8102. The files were downloaded from www.MorphoSource.org, Duke University.
MCZ-23813	doi:10.17602/M2/M3031	2.25 GB	MCZ 23813 skull	Macaca fascicularis	0.061559 mm	0.061559 mm	0.061559 mm	80 kv	125 A	10 W	1050	MCZ - CC BY-NC	Citation: Lynn Lucas and Lynn Copes provided access to these data, the collection of which was funded by NSF DDIG #0925793 and Wenner Gren Foundation #8102. The files were downloaded from www.MorphoSource.org, Duke University.
MCZ-23986	doi:10.17602/M2/M4441	4.63 GB	MCZ 23986 cranium	Therapithecus gelada	0.094522 mm	0.094522 mm	0.094522 mm	85 kv	125 A	10 W	1100	MCZ - CC BY-NC	Citation: Lynn Lucas and Lynn Copes provided access to these data, the collection of which was funded by NSF DDIG #0925793 and Wenner Gren Foundation #8102. The files were downloaded from www.MorphoSource.org, Duke University.
MCZ-23986	doi:10.17602/M2/M4440	3.84 GB	MCZ 23986 mandible	Therapithecus gelada	0.07805 mm	0.07805 mm	0.07805 mm	85 kv	125 A	10 W	1100	MCZ - CC BY-NC	Citation: Lynn Lucas and Lynn Copes provided access to these data, the collection of which was funded by NSF DDIG #0925793 and Wenner Gren Foundation #8102. The files were downloaded from www.MorphoSource.org, Duke University.
MCZ-24080	doi:10.17602/M2/M2892	1.87 GB	MCZ 24080 skull	Colobus badius	0.079977 mm	0.079977 mm	0.079977 mm	80 kv	125 A	10 W	1100	MCZ - CC BY-NC	Citation: Lynn Lucas and Lynn Copes provided access to these data, the collection of which was funded by NSF DDIG #0925793 and Wenner Gren Foundation #8102. The files were downloaded from www.MorphoSource.org, Duke University.
MCZ-24775	doi:10.17602/M2/M2893	2.13 GB	MCZ 24775 skull	Colobus badius	0.08971 mm	0.08971 mm	0.08971 mm	80 kv	125 A	10 W	1100	MCZ - CC BY-NC	Citation: Lynn Lucas and Lynn Copes provided access to these data, the collection of which was funded by NSF DDIG #0925793 and Wenner Gren Foundation #8102. The files were downloaded from www.MorphoSource.org, Duke University.
MCZ-24793	doi:10.17602/M2/M2894	2.49 GB	MCZ 24793 skull	Colobus badius	0.079977 mm	0.079977 mm	0.079977 mm	80 kv	125 A	10 W	1100	MCZ - CC BY-NC	Citation: Lynn Lucas and Lynn Copes provided access to these data, the collection of which was funded by NSF DDIG #0925793 and Wenner Gren Foundation #8102. The files were downloaded from www.MorphoSource.org, Duke University.
MCZ-25022	doi:10.17602/M2/M2929	1.23 GB	MCZ 25022 skull	Cercopithecus mitis	0.079988 mm	0.079988 mm	0.079988 mm	80 kv	125 A	10 W	1100	MCZ - CC BY-NC	Citation: Lynn Lucas and Lynn Copes provided access to these data, the collection of which was funded by NSF DDIG #0925793 and Wenner Gren Foundation #8102. The files were downloaded from www.MorphoSource.org, Duke University.
MCZ-25626	doi:10.17602/M2/M2353	2.77 GB	MCZ 25626 skull	Cercocebus torquatus	0.092963 mm	0.092963 mm	0.092963 mm	85 kv	125 A	10.6 W	1100	MCZ - CC BY-NC	Citation: Lynn Lucas and Lynn Copes provided access to these data, the collection of which was funded by NSF DDIG #0925793 and Wenner Gren Foundation #8102. The files were downloaded from www.MorphoSource.org, Duke University.
MCZ-25627	doi:10.17602/M2/M2895	2.11 GB	MCZ 25627 skull	Colobus badius	0.079977 mm	0.079977 mm	0.079977 mm	80 kv	125 A	10 W	1100	MCZ - CC BY-NC	Citation: Lynn Lucas and Lynn Copes provided access to these data, the collection of which was funded by NSF DDIG #0925793 and Wenner Gren Foundation #8102. The files were downloaded from www.MorphoSource.org, Duke University.
MCZ-25630	doi:10.17602/M2/M2354	2.45 GB	MCZ 25630 skull	Cercocebus torquatus	0.092963 mm	0.092963 mm	0.092963 mm	85 kv	125 A	10.6 W	1100	MCZ - CC BY-NC	Citation: Lynn Lucas and Lynn Copes provided access to these data, the collection of which was funded by NSF DDIG #0925793 and Wenner Gren Foundation #8102. The files were downloaded from www.MorphoSource.org, Duke University.
MCZ-25631	doi:10.17602/M2/M2896	2.45 GB	MCZ 25631 skull	Colobus badius	0.08971 mm	0.08971 mm	0.08971 mm	80 kv	125 A	10 W	1100	MCZ - CC BY-NC	Citation: Lynn Lucas and Lynn Copes provided access to these data, the collection of which was funded by NSF DDIG #0925793 and Wenner Gren Foundation #8102. The files were downloaded from www.MorphoSource.org, Duke University.
MCZ-25810	doi:10.17602/M2/M2897	1.94 GB	MCZ 25810 skull	Colobus badius	0.079977 mm	0.079977 mm	0.079977 mm	80 kv	125 A	10 W	1100	MCZ - CC BY-NC	Citation: Lynn Lucas and Lynn Copes provided access to these data, the collection of which was funded by NSF DDIG #0925793 and Wenner Gren Foundation #8102. The files were downloaded from www.MorphoSource.org, Duke University.
MCZ-25811	doi:10.17602/M2/M5205	3.66 GB	MCZ 25811 skull	Cebus apella	0.059654 mm	0.059654 mm	0.059654 mm	90 kv	120 A	10.8 W	1100	MCZ - CC BY-NC	Citation: Lynn Lucas and Lynn Copes provided access to these data, the collection of which was funded by NSF DDIG #0925793 and Wenner Gren Foundation #8102. The files were downloaded from www.MorphoSource.org, Duke University.
MCZ-25812	doi:10.17602/M2/M2850	1.36 GB	MCZ 25812 skull	Alouatta caraya	0.109004 mm	0.109004 mm	0.109004 mm	80 kv	125 A	10 W	1500	MCZ - CC BY-NC	Citation: Lynn Lucas and Lynn Copes provided access to these data, the collection of which was funded by NSF DDIG #0925793 and Wenner Gren Foundation #8102. The files were downloaded from www.MorphoSource.org, Duke University.
MCZ-25831	doi:10.17602/M2/M4717	3.64 GB	MCZ 25831 skull	Perodicticus potto	0.039518 mm	0.039518 mm	0.039518 mm	80 kv	125 A	10 W	1000	MCZ - CC BY-NC	Citation: Lynn Lucas and Lynn Copes provided access to these data, the collection of which was funded by NSF DDIG #0925793 and Wenner Gren Foundation #8102. The files were downloaded from www.MorphoSource.org, Duke University.
MCZ-26473	doi:10.17602/M2/M4890	2.73 GB	MCZ 26473 skull	Papio doguera	0.099987 mm	0.099987 mm	0.099987 mm	85 kv	125 A	10 W	1100	MCZ - CC BY-NC	Citation: Lynn Lucas and Lynn Copes provided access to these data, the collection of which was funded by NSF DDIG #0925793 and Wenner Gren Foundation #8102. The files were downloaded from www.MorphoSource.org, Duke University.
MCZ-26475	doi:10.17602/M2/M3052	2.27 GB	MCZ 26475 skull	Macaca mulatta	0.090751 mm	0.090751 mm	0.090751 mm	80 kv	110 A	8.8 W	1050	MCZ - CC BY-NC	Citation: Lynn Lucas and Lynn Copes provided access to these data, the collection of which was funded by NSF DDIG #0925793 and Wenner Gren Foundation #8102. The files were downloaded from www.MorphoSource.org, Duke University.
MCZ-26552	doi:10.17602/M2/M2898	2.26 GB	MCZ 26552 skull	Colobus badius	0.079977 mm	0.079977 mm	0.079977 mm	80 kv	125 A	10 W	1100	MCZ - CC BY-NC	Citation: Lynn Lucas and Lynn Copes provided access to these data, the collection of which was funded by NSF DDIG #0925793 and Wenner Gren Foundation #8102. The files were downloaded from www.MorphoSource.org, Duke University.
MCZ-26553	doi:10.17602/M2/M2899	2.01 GB	MCZ 26553 skull	Colobus badius	0.08971 mm	0.08971 mm	0.08971 mm	80 kv	125 A	10 W	1100	MCZ - CC BY-NC	Citation: Lynn Lucas and Lynn Copes provided access to these data, the collection of which was funded by NSF DDIG #0925793 and Wenner Gren Foundation #8102. The files were downloaded from www.MorphoSource.org, Duke University.
MCZ-26832	doi:10.17602/M2/M2930	1.89 GB	MCZ 26832 skull	Cercopithecus mitis	0.079988 mm	0.079988 mm	0.079988 mm	80 kv	125 A	10 W	1100	MCZ - CC BY-NC	Citation: Lynn Lucas and Lynn Copes provided access to these data, the collection of which was funded by NSF DDIG #0925793 and Wenner Gren Foundation #8102. The files were downloaded from www.MorphoSource.org, Duke University.
MCZ-26847	doi:10.17602/M2/M4389	3.78 GB	MCZ 26847 skull	Pan troglodytes	0.099918 mm	0.099918 mm	0.099918 mm	80 kv	120 A	9.6 W	1500	MCZ - CC BY-NC	Citation: Lynn Lucas and Lynn Copes provided access to these data, the collection of which was funded by NSF DDIG #0925793 and Wenner Gren Foundation #8102. The files were downloaded from www.MorphoSource.org, Duke University.
MCZ-26849	doi:10.17602/M2/M4388	3.95 GB	MCZ 26849 cranium	Pan troglodytes	0.105636 mm	0.105636 mm	0.105636 mm	80 kv	120 A	9.6 W	1500	MCZ - CC BY-NC	Citation: Lynn Lucas and Lynn Copes provided access to these data, the collection of which was funded by NSF DDIG #0925793 and Wenner Gren Foundation #8102. The files were downloaded from www.MorphoSource.org, Duke University.
MCZ-26850	doi:10.17602/M2/M2947	3.06 GB	MCZ 26850 cranium	Gorilla gorilla gorilla	0.123431 mm	0.123431 mm	0.123431 mm	85 kv	90 A	7.7 W	1500	MCZ - CC BY-NC	Citation: Lynn Lucas and Lynn Copes provided access to these data, the collection of which was funded by NSF DDIG #0925793 and Wenner Gren Foundation #8102. The files were downloaded from www.MorphoSource.org, Duke University.
MCZ-26850	doi:10.17602/M2/M2948	4.74 GB	MCZ 26850 mandible	Gorilla gorilla gorilla	0.093574 mm	0.093574 mm	0.093574 mm	85 kv	90 A	7.7 W	1000	MCZ - CC BY-NC	Citation: Lynn Lucas and Lynn Copes provided access to these data, the collection of which was funded by NSF DDIG #0925793 and Wenner Gren Foundation #8102. The files were downloaded from www.MorphoSource.org, Duke University.
MCZ-26922	doi:10.17602/M2/M5160	2.55 GB	MCZ 26922 skull	Callicebus moloch	0.047097 mm	0.047097 mm	0.047097 mm	80 kv	120 A	9.6 W	1050	MCZ - CC BY-NC	Citation: Lynn Lucas and Lynn Copes provided access to these data, the collection of which was funded by NSF DDIG #0925793 and Wenner Gren Foundation #8102. The files were downloaded from www.MorphoSource.org, Duke University.
MCZ-27097	doi:10.17602/M2/M5206	4.03 GB	MCZ 27097 skull	Cebus apella	0.058896 mm	0.058896 mm	0.058896 mm	80 kv	115 A	9.2 W	1100	MCZ - CC BY-NC	Citation: Lynn Lucas and Lynn Copes provided access to these data, the collection of which was funded by NSF DDIG #0925793 and Wenner Gren Foundation #8102. The files were downloaded from www.MorphoSource.org, Duke University.
MCZ-27098	doi:10.17602/M2/M5207	1.23 GB	MCZ 27098 skull	Cebus apella	0.072065 mm	0.072065 mm	0.072065 mm	80 kv	115 A	9.2 W	1100	MCZ - CC BY-NC	Citation: Lynn Lucas and Lynn Copes provided access to these data, the collection of which was funded by NSF DDIG #0925793 and Wenner Gren Foundation #8102. The files were downloaded from www.MorphoSource.org, Duke University.
MCZ-27108	doi:10.17602/M2/M2900	2.73 GB	MCZ 27108 skull	Colobus badius	0.079977 mm	0.079977 mm	0.079977 mm	80 kv	125 A	10 W	1100	MCZ - CC BY-NC	Citation: Lynn Lucas and Lynn Copes provided access to these data, the collection of which was funded by NSF DDIG #0925793 and Wenner Gren Foundation #8102. The files were downloaded from www.MorphoSource.org, Duke University.
MCZ-27124	doi:10.17602/M2/M4708	3.41 GB	MCZ 27124 skull	Pithecia monachus	0.050092 mm	0.050092 mm	0.050092 mm	80 kv	115 A	9.2 W	1050	MCZ - CC BY-NC	Citation: Lynn Lucas and Lynn Copes provided access to these data, the collection of which was funded by NSF DDIG #0925793 and Wenner Gren Foundation #8102. The files were downloaded from www.MorphoSource.org, Duke University.
MCZ-27197	doi:10.17602/M2/M4485	2 GB	MCZ 27197 skull	Saimiri sciureus	0.047018 mm	0.047018 mm	0.047018 mm	80 kv	120 A	9.6 W	1050	MCZ - CC BY-NC	Citation: Lynn Lucas and Lynn Copes provided access to these data, the collection of which was funded by NSF DDIG #0925793 and Wenner Gren Foundation #8102. The files were downloaded from www.MorphoSource.org, Duke University.
MCZ-27214	doi:10.17602/M2/M2874	2.66 GB	MCZ 27214 skull	Aotus trivirgatus	0.040724 mm	0.040724 mm	0.040724 mm	80 kv	125 A	10 W	1000	MCZ - CC BY-NC	Citation: Lynn Lucas and Lynn Copes provided access to these data, the collection of which was funded by NSF DDIG #0925793 and Wenner Gren Foundation #8102. The files were downloaded from www.MorphoSource.org, Duke University.
MCZ-27331	doi:10.17602/M2/M4500	1.05 GB	MCZ 27331 skull	Saguinus sp.	0.040724 mm	0.040724 mm	0.040724 mm	80 kv	125 A	10 W	1000	MCZ - CC BY-NC	Citation: Lynn Lucas and Lynn Copes provided access to these data, the collection of which was funded by NSF DDIG #0925793 and Wenner Gren Foundation #8102. The files were downloaded from www.MorphoSource.org, Duke University.
MCZ-27332	doi:10.17602/M2/M4499	1.07 GB	MCZ 27332 skull	Saguinus sp.	0.040724 mm	0.040724 mm	0.040724 mm	80 kv	125 A	10 W	1000	MCZ - CC BY-NC	Citation: Lynn Lucas and Lynn Copes provided access to these data, the collection of which was funded by NSF DDIG #0925793 and Wenner Gren Foundation #8102. The files were downloaded from www.MorphoSource.org, Duke University.
MCZ-27785	doi:10.17602/M2/M2865	1.85 GB	MCZ 27785 skull	Alouatta palliata	0.079988 mm	0.079988 mm	0.079988 mm	85 kv	125 A	10.6 W	1100	MCZ - CC BY-NC	Citation: Lynn Lucas and Lynn Copes provided access to these data, the collection of which was funded by NSF DDIG #0925793 and Wenner Gren Foundation #8102. The files were downloaded from www.MorphoSource.org, Duke University.
MCZ-27869	doi:10.17602/M2/M2718	4.86 GB	MCZ 27869 skull	Nycticebus coucang	0.024597 mm	0.024597 mm	0.024597 mm	80 kv	125 A	10 W	1000	MCZ - CC BY-NC	Citation: Lynn Lucas and Lynn Copes provided access to these data, the collection of which was funded by NSF DDIG #0925793 and Wenner Gren Foundation #8102. The files were downloaded from www.MorphoSource.org, Duke University.
MCZ-27870	doi:10.17602/M2/M5157	1.55 GB	MCZ 27870 skull	Cacajao calvus rubicundus	0.079988 mm	0.079988 mm	0.079988 mm	80 kv	115 A	9.2 W	1100	MCZ - CC BY-NC	Citation: Lynn Lucas and Lynn Copes provided access to these data, the collection of which was funded by NSF DDIG #0925793 and Wenner Gren Foundation #8102. The files were downloaded from www.MorphoSource.org, Duke University.
MCZ-27891	doi:10.17602/M2/M5208	3.02 GB	MCZ 27891 skull	Cebus apella	0.059654 mm	0.059654 mm	0.059654 mm	90 kv	120 A	10.8 W	1100	MCZ - CC BY-NC	Citation: Lynn Lucas and Lynn Copes provided access to these data, the collection of which was funded by NSF DDIG #0925793 and Wenner Gren Foundation #8102. The files were downloaded from www.MorphoSource.org, Duke University.
MCZ-28095	doi:10.17602/M2/M2851	1.55 GB	MCZ 28095 skull	Alouatta caraya	0.109004 mm	0.109004 mm	0.109004 mm	80 kv	125 A	10 W	1500	MCZ - CC BY-NC	Citation: Lynn Lucas and Lynn Copes provided access to these data, the collection of which was funded by NSF DDIG #0925793 and Wenner Gren Foundation #8102. The files were downloaded from www.MorphoSource.org, Duke University.
MCZ-28096	doi:10.17602/M2/M2852	1.28 GB	MCZ 28096 skull	Alouatta caraya	0.079988 mm	0.079988 mm	0.079988 mm	80 kv	125 A	10 W	1100	MCZ - CC BY-NC	Citation: Lynn Lucas and Lynn Copes provided access to these data, the collection of which was funded by NSF DDIG #0925793 and Wenner Gren Foundation #8102. The files were downloaded from www.MorphoSource.org, Duke University.
MCZ-28654	doi:10.17602/M2/M2853	1.44 GB	MCZ 28654 skull	Alouatta caraya	0.109441 mm	0.109441 mm	0.109441 mm	80 kv	125 A	10 W	1500	MCZ - CC BY-NC	Citation: Lynn Lucas and Lynn Copes provided access to these data, the collection of which was funded by NSF DDIG #0925793 and Wenner Gren Foundation #8102. The files were downloaded from www.MorphoSource.org, Duke University.
MCZ-28655	doi:10.17602/M2/M2854	2.04 GB	MCZ 28655 skull	Alouatta caraya	0.079988 mm	0.079988 mm	0.079988 mm	85 kv	125 A	10.6 W	1100	MCZ - CC BY-NC	Citation: Lynn Lucas and Lynn Copes provided access to these data, the collection of which was funded by NSF DDIG #0925793 and Wenner Gren Foundation #8102. The files were downloaded from www.MorphoSource.org, Duke University.
MCZ-28679	doi:10.17602/M2/M5209	1.84 GB	MCZ 28679 skull	Cebus apella	0.07105 mm	0.07105 mm	0.07105 mm	80 kv	115 A	9.2 W	1100	MCZ - CC BY-NC	Citation: Lynn Lucas and Lynn Copes provided access to these data, the collection of which was funded by NSF DDIG #0925793 and Wenner Gren Foundation #8102. The files were downloaded from www.MorphoSource.org, Duke University.
MCZ-28713	doi:10.17602/M2/M2855	1.11 GB	MCZ 28713 skull	Alouatta caraya	0.079988 mm	0.079988 mm	0.079988 mm	80 kv	125 A	10 W	1100	MCZ - CC BY-NC	Citation: Lynn Lucas and Lynn Copes provided access to these data, the collection of which was funded by NSF DDIG #0925793 and Wenner Gren Foundation #8102. The files were downloaded from www.MorphoSource.org, Duke University.
MCZ-29488	doi:10.17602/M2/M4484	2.03 GB	MCZ 29488 skull	Saimiri oerstedii	0.047018 mm	0.047018 mm	0.047018 mm	80 kv	120 A	9.6 W	1050	MCZ - CC BY-NC	Citation: Lynn Lucas and Lynn Copes provided access to these data, the collection of which was funded by NSF DDIG #0925793 and Wenner Gren Foundation #8102. The files were downloaded from www.MorphoSource.org, Duke University.
MCZ-29609	doi:10.17602/M2/M2866	1.41 GB	MCZ 29609 skull	Alouatta palliata	0.079988 mm	0.079988 mm	0.079988 mm	85 kv	125 A	10.6 W	1100	MCZ - CC BY-NC	Citation: Lynn Lucas and Lynn Copes provided access to these data, the collection of which was funded by NSF DDIG #0925793 and Wenner Gren Foundation #8102. The files were downloaded from www.MorphoSource.org, Duke University.
MCZ-29611	doi:10.17602/M2/M2867	2.08 GB	MCZ 29611 skull	Alouatta palliata	0.079988 mm	0.079988 mm	0.079988 mm	85 kv	125 A	10.6 W	1100	MCZ - CC BY-NC	Citation: Lynn Lucas and Lynn Copes provided access to these data, the collection of which was funded by NSF DDIG #0925793 and Wenner Gren Foundation #8102. The files were downloaded from www.MorphoSource.org, Duke University.
MCZ-29626	doi:10.17602/M2/M2918	4.7 GB	MCZ 29626 skull	Ateles geoffroyi	0.06093 mm	0.06093 mm	0.06093 mm	90 kv	120 A	10.8 W	1100	MCZ - CC BY-NC	Citation: Lynn Lucas and Lynn Copes provided access to these data, the collection of which was funded by NSF DDIG #0925793 and Wenner Gren Foundation #8102. The files were downloaded from www.MorphoSource.org, Duke University.
MCZ-29628	doi:10.17602/M2/M2919	3.15 GB	MCZ 29628 skull	Ateles geoffroyi	0.063468 mm	0.063468 mm	0.063468 mm	90 kv	120 A	10.8 W	1100	MCZ - CC BY-NC	Citation: Lynn Lucas and Lynn Copes provided access to these data, the collection of which was funded by NSF DDIG #0925793 and Wenner Gren Foundation #8102. The files were downloaded from www.MorphoSource.org, Duke University.
MCZ-29658	doi:10.17602/M2/M2920	4.45 GB	MCZ 29658 skull	Ateles geoffroyi	0.063468 mm	0.063468 mm	0.063468 mm	90 kv	120 A	10.8 W	1100	MCZ - CC BY-NC	Citation: Lynn Lucas and Lynn Copes provided access to these data, the collection of which was funded by NSF DDIG #0925793 and Wenner Gren Foundation #8102. The files were downloaded from www.MorphoSource.org, Duke University.
MCZ-29786	doi:10.17602/M2/M4877	2.49 GB	MCZ 29786 cranium	Papio doguera ibeanus	0.117658 mm	0.117658 mm	0.117658 mm	80 kv	125 A	10 W	1100	MCZ - CC BY-NC	Citation: Lynn Lucas and Lynn Copes provided access to these data, the collection of which was funded by NSF DDIG #0925793 and Wenner Gren Foundation #8102. The files were downloaded from www.MorphoSource.org, Duke University.
MCZ-29786	doi:10.17602/M2/M4878	3.2 GB	MCZ 29786 mandible	Papio doguera ibeanus	0.082975 mm	0.082975 mm	0.082975 mm	90 kv	125 A	11.3 W	1100	MCZ - CC BY-NC	Citation: Lynn Lucas and Lynn Copes provided access to these data, the collection of which was funded by NSF DDIG #0925793 and Wenner Gren Foundation #8102. The files were downloaded from www.MorphoSource.org, Duke University.
MCZ-29787	doi:10.17602/M2/M4891	2.37 GB	MCZ 29787 skull	Papio doguera	0.099987 mm	0.099987 mm	0.099987 mm	85 kv	125 A	10 W	1100	MCZ - CC BY-NC	Citation: Lynn Lucas and Lynn Copes provided access to these data, the collection of which was funded by NSF DDIG #0925793 and Wenner Gren Foundation #8102. The files were downloaded from www.MorphoSource.org, Duke University.
MCZ-29794	doi:10.17602/M2/M2912	2.34 GB	MCZ 29794 skull	Colobus polykomos	0.054046 mm	0.054046 mm	0.054046 mm	85 kv	125 A	10.6 W	1000	MCZ - CC BY-NC	Citation: Lynn Lucas and Lynn Copes provided access to these data, the collection of which was funded by NSF DDIG #0925793 and Wenner Gren Foundation #8102. The files were downloaded from www.MorphoSource.org, Duke University.
MCZ-30384	doi:10.17602/M2/M3049	2.42 GB	MCZ 30384 skull	Macaca mulatta	0.090751 mm	0.090751 mm	0.090751 mm	80 kv	110 A	8.8 W	1050	MCZ - CC BY-NC	Citation: Lynn Lucas and Lynn Copes provided access to these data, the collection of which was funded by NSF DDIG #0925793 and Wenner Gren Foundation #8102. The files were downloaded from www.MorphoSource.org, Duke University.
MCZ-30559	doi:10.17602/M2/M5161	1.62 GB	MCZ 30559 skull	Callicebus moloch	0.047097 mm	0.047097 mm	0.047097 mm	80 kv	120 A	9.6 W	1050	MCZ - CC BY-NC	Citation: Lynn Lucas and Lynn Copes provided access to these data, the collection of which was funded by NSF DDIG #0925793 and Wenner Gren Foundation #8102. The files were downloaded from www.MorphoSource.org, Duke University.
MCZ-30562	doi:10.17602/M2/M2875	2.71 GB	MCZ 30562 skull	Aotus trivirgatus	0.040724 mm	0.040724 mm	0.040724 mm	80 kv	125 A	10 W	1000	MCZ - CC BY-NC	Citation: Lynn Lucas and Lynn Copes provided access to these data, the collection of which was funded by NSF DDIG #0925793 and Wenner Gren Foundation #8102. The files were downloaded from www.MorphoSource.org, Duke University.
MCZ-30564	doi:10.17602/M2/M5167	1.46 GB	MCZ 30564 skull	Callicebus moloch	0.047097 mm	0.047097 mm	0.047097 mm	80 kv	120 A	9.6 W	1050	MCZ - CC BY-NC	Citation: Lynn Lucas and Lynn Copes provided access to these data, the collection of which was funded by NSF DDIG #0925793 and Wenner Gren Foundation #8102. The files were downloaded from www.MorphoSource.org, Duke University.
MCZ-30566	doi:10.17602/M2/M5168	1.51 GB	MCZ 30566 skull	Callicebus moloch	0.047097 mm	0.047097 mm	0.047097 mm	80 kv	120 A	9.6 W	1050	MCZ - CC BY-NC	Citation: Lynn Lucas and Lynn Copes provided access to these data, the collection of which was funded by NSF DDIG #0925793 and Wenner Gren Foundation #8102. The files were downloaded from www.MorphoSource.org, Duke University.
MCZ-30568	doi:10.17602/M2/M4483	980.67 MB	MCZ 30568 skull	Saimiri sciureus	0.047018 mm	0.047018 mm	0.047018 mm	80 kv	120 A	9.6 W	1050	MCZ - CC BY-NC	Citation: Lynn Lucas and Lynn Copes provided access to these data, the collection of which was funded by NSF DDIG #0925793 and Wenner Gren Foundation #8102. The files were downloaded from www.MorphoSource.org, Duke University.
MCZ-30569	doi:10.17602/M2/M4447	1.45 GB	MCZ 30569 skull	Saimiri sciureus	0.047018 mm	0.047018 mm	0.047018 mm	80 kv	120 A	9.6 W	1050	MCZ - CC BY-NC	Citation: Lynn Lucas and Lynn Copes provided access to these data, the collection of which was funded by NSF DDIG #0925793 and Wenner Gren Foundation #8102. The files were downloaded from www.MorphoSource.org, Duke University.
MCZ-30572	doi:10.17602/M2/M4446	1.5 GB	MCZ 30572 skull	Saimiri sciureus	0.047018 mm	0.047018 mm	0.047018 mm	80 kv	120 A	9.6 W	1050	MCZ - CC BY-NC	Citation: Lynn Lucas and Lynn Copes provided access to these data, the collection of which was funded by NSF DDIG #0925793 and Wenner Gren Foundation #8102. The files were downloaded from www.MorphoSource.org, Duke University.
MCZ-30577	doi:10.17602/M2/M5199	1.21 GB	MCZ 30577 skull	Callithrix humeralifera	0.040724 mm	0.040724 mm	0.040724 mm	80 kv	125 A	10 W	1000	MCZ - CC BY-NC	Citation: Lynn Lucas and Lynn Copes provided access to these data, the collection of which was funded by NSF DDIG #0925793 and Wenner Gren Foundation #8102. The files were downloaded from www.MorphoSource.org, Duke University.
MCZ-30579	doi:10.17602/M2/M5192	1.27 GB	MCZ 30579 skull	Callithrix argentata	0.040724 mm	0.040724 mm	0.040724 mm	80 kv	125 A	10 W	1000	MCZ - CC BY-NC	Citation: Lynn Lucas and Lynn Copes provided access to these data, the collection of which was funded by NSF DDIG #0925793 and Wenner Gren Foundation #8102. The files were downloaded from www.MorphoSource.org, Duke University.
MCZ-30580	doi:10.17602/M2/M5193	980.6 MB	MCZ 30580 skull	Callithrix argentata	0.040724 mm	0.040724 mm	0.040724 mm	80 kv	125 A	10 W	1000	MCZ - CC BY-NC	Citation: Lynn Lucas and Lynn Copes provided access to these data, the collection of which was funded by NSF DDIG #0925793 and Wenner Gren Foundation #8102. The files were downloaded from www.MorphoSource.org, Duke University.
MCZ-30582	doi:10.17602/M2/M5194	1.06 GB	MCZ 30582 skull	Callithrix argentata	0.040724 mm	0.040724 mm	0.040724 mm	80 kv	125 A	10 W	1000	MCZ - CC BY-NC	Citation: Lynn Lucas and Lynn Copes provided access to these data, the collection of which was funded by NSF DDIG #0925793 and Wenner Gren Foundation #8102. The files were downloaded from www.MorphoSource.org, Duke University.
MCZ-30583	doi:10.17602/M2/M5195	944.49 MB	MCZ 30583 skull	Callithrix argentata	0.040724 mm	0.040724 mm	0.040724 mm	80 kv	125 A	10 W	1000	MCZ - CC BY-NC	Citation: Lynn Lucas and Lynn Copes provided access to these data, the collection of which was funded by NSF DDIG #0925793 and Wenner Gren Foundation #8102. The files were downloaded from www.MorphoSource.org, Duke University.
MCZ-30586	doi:10.17602/M2/M5200	903.76 MB	MCZ 30586 skull	Callithrix humeralifera	0.040724 mm	0.040724 mm	0.040724 mm	80 kv	125 A	10 W	1000	MCZ - CC BY-NC	Citation: Lynn Lucas and Lynn Copes provided access to these data, the collection of which was funded by NSF DDIG #0925793 and Wenner Gren Foundation #8102. The files were downloaded from www.MorphoSource.org, Duke University.
MCZ-30597	doi:10.17602/M2/M4498	1 GB	MCZ 30597 skull	Saguinus sp.	0.040724 mm	0.040724 mm	0.040724 mm	80 kv	125 A	10 W	1000	MCZ - CC BY-NC	Citation: Lynn Lucas and Lynn Copes provided access to these data, the collection of which was funded by NSF DDIG #0925793 and Wenner Gren Foundation #8102. The files were downloaded from www.MorphoSource.org, Duke University.
MCZ-30601	doi:10.17602/M2/M4497	1.03 GB	MCZ 30601 skull	Saguinus sp.	0.040724 mm	0.040724 mm	0.040724 mm	80 kv	125 A	10 W	1000	MCZ - CC BY-NC	Citation: Lynn Lucas and Lynn Copes provided access to these data, the collection of which was funded by NSF DDIG #0925793 and Wenner Gren Foundation #8102. The files were downloaded from www.MorphoSource.org, Duke University.
MCZ-30603	doi:10.17602/M2/M5201	1.15 GB	MCZ 30603 skull	Callithrix humeralifera	0.040724 mm	0.040724 mm	0.040724 mm	80 kv	125 A	10 W	1000	MCZ - CC BY-NC	Citation: Lynn Lucas and Lynn Copes provided access to these data, the collection of which was funded by NSF DDIG #0925793 and Wenner Gren Foundation #8102. The files were downloaded from www.MorphoSource.org, Duke University.
MCZ-30718	doi:10.17602/M2/M4707	2.67 GB	MCZ 30718 cranium	Pithecia pithecia	0.050092 mm	0.050092 mm	0.050092 mm	80 kv	115 A	9.2 W	1050	MCZ - CC BY-NC	Citation: Lynn Lucas and Lynn Copes provided access to these data, the collection of which was funded by NSF DDIG #0925793 and Wenner Gren Foundation #8102. The files were downloaded from www.MorphoSource.org, Duke University.
MCZ-30719	doi:10.17602/M2/M4706	3.19 GB	MCZ 30719 skull	Pithecia pithecia	0.050092 mm	0.050092 mm	0.050092 mm	80 kv	115 A	9.2 W	1050	MCZ - CC BY-NC	Citation: Lynn Lucas and Lynn Copes provided access to these data, the collection of which was funded by NSF DDIG #0925793 and Wenner Gren Foundation #8102. The files were downloaded from www.MorphoSource.org, Duke University.
MCZ-30720	doi:10.17602/M2/M4705	2.55 GB	MCZ 30720 skull	Pithecia monachus	0.050092 mm	0.050092 mm	0.050092 mm	80 kv	115 A	9.2 W	1050	MCZ - CC BY-NC	Citation: Lynn Lucas and Lynn Copes provided access to these data, the collection of which was funded by NSF DDIG #0925793 and Wenner Gren Foundation #8102. The files were downloaded from www.MorphoSource.org, Duke University.
MCZ-30724	doi:10.17602/M2/M5210	1.72 GB	MCZ 30724 skull	Cebus apella	0.067041 mm	0.067041 mm	0.067041 mm	80 kv	115 A	9.2 W	1100	MCZ - CC BY-NC	Citation: Lynn Lucas and Lynn Copes provided access to these data, the collection of which was funded by NSF DDIG #0925793 and Wenner Gren Foundation #8102. The files were downloaded from www.MorphoSource.org, Duke University.
MCZ-30726	doi:10.17602/M2/M5211	1.83 GB	MCZ 30726 skull	Cebus apella	0.072065 mm	0.072065 mm	0.072065 mm	80 kv	115 A	9.2 W	1100	MCZ - CC BY-NC	Citation: Lynn Lucas and Lynn Copes provided access to these data, the collection of which was funded by NSF DDIG #0925793 and Wenner Gren Foundation #8102. The files were downloaded from www.MorphoSource.org, Duke University.
MCZ-31061	doi:10.17602/M2/M4704	3.24 GB	MCZ 31061 skull	Pithecia pithecia	0.050092 mm	0.050092 mm	0.050092 mm	80 kv	115 A	9.2 W	1050	MCZ - CC BY-NC	Citation: Lynn Lucas and Lynn Copes provided access to these data, the collection of which was funded by NSF DDIG #0925793 and Wenner Gren Foundation #8102. The files were downloaded from www.MorphoSource.org, Duke University.
MCZ-31062	doi:10.17602/M2/M5212	1.14 GB	MCZ 31062 skull	Cebus apella	0.079989 mm	0.079989 mm	0.079989 mm	80 kv	115 A	9.2 W	1100	MCZ - CC BY-NC	Citation: Lynn Lucas and Lynn Copes provided access to these data, the collection of which was funded by NSF DDIG #0925793 and Wenner Gren Foundation #8102. The files were downloaded from www.MorphoSource.org, Duke University.
MCZ-31063	doi:10.17602/M2/M5213	1.78 GB	MCZ 31063 skull	Cebus apella	0.067041 mm	0.067041 mm	0.067041 mm	80 kv	115 A	9.2 W	1100	MCZ - CC BY-NC	Citation: Lynn Lucas and Lynn Copes provided access to these data, the collection of which was funded by NSF DDIG #0925793 and Wenner Gren Foundation #8102. The files were downloaded from www.MorphoSource.org, Duke University.
MCZ-31064	doi:10.17602/M2/M5214	2.16 GB	MCZ 31064 skull	Cebus apella	0.072065 mm	0.072065 mm	0.072065 mm	80 kv	115 A	9.2 W	1100	MCZ - CC BY-NC	Citation: Lynn Lucas and Lynn Copes provided access to these data, the collection of which was funded by NSF DDIG #0925793 and Wenner Gren Foundation #8102. The files were downloaded from www.MorphoSource.org, Duke University.
MCZ-31066	doi:10.17602/M2/M5215	1.31 GB	MCZ 31066 skull	Cebus apella	0.072065 mm	0.072065 mm	0.072065 mm	80 kv	115 A	9.2 W	1100	MCZ - CC BY-NC	Citation: Lynn Lucas and Lynn Copes provided access to these data, the collection of which was funded by NSF DDIG #0925793 and Wenner Gren Foundation #8102. The files were downloaded from www.MorphoSource.org, Duke University.
MCZ-31072	doi:10.17602/M2/M5216	1.71 GB	MCZ 31072 skull	Cebus apella	0.067041 mm	0.067041 mm	0.067041 mm	80 kv	115 A	9.2 W	1100	MCZ - CC BY-NC	Citation: Lynn Lucas and Lynn Copes provided access to these data, the collection of which was funded by NSF DDIG #0925793 and Wenner Gren Foundation #8102. The files were downloaded from www.MorphoSource.org, Duke University.
MCZ-31619	doi:10.17602/M2/M4892	3.01 GB	MCZ 31619 skull	Papio doguera	0.10745 mm	0.10745 mm	0.10745 mm	85 kv	125 A	10 W	1100	MCZ - CC BY-NC	Citation: Lynn Lucas and Lynn Copes provided access to these data, the collection of which was funded by NSF DDIG #0925793 and Wenner Gren Foundation #8102. The files were downloaded from www.MorphoSource.org, Duke University.
MCZ-31701	doi:10.17602/M2/M5145	3.91 GB	MCZ 31701 skull	Chiropotes albinasus	0.052569 mm	0.052569 mm	0.052569 mm	80 kv	115 A	9.2 W	1100	MCZ - CC BY-NC	Citation: Lynn Lucas and Lynn Copes provided access to these data, the collection of which was funded by NSF DDIG #0925793 and Wenner Gren Foundation #8102. The files were downloaded from www.MorphoSource.org, Duke University.
MCZ-31720	doi:10.17602/M2/M4716	3.62 GB	MCZ 31720 skull	Perodicticus potto	0.036722 mm	0.036722 mm	0.036722 mm	80 kv	125 A	10 W	1000	MCZ - CC BY-NC	Citation: Lynn Lucas and Lynn Copes provided access to these data, the collection of which was funded by NSF DDIG #0925793 and Wenner Gren Foundation #8102. The files were downloaded from www.MorphoSource.org, Duke University.
MCZ-31723	doi:10.17602/M2/M2686	3.84 GB	MCZ 31723 skull	Galago senegalensis	0.024143 mm	0.024143 mm	0.024143 mm	80 kv	125 A	10 W	1000	MCZ - CC BY-NC	Citation: Lynn Lucas and Lynn Copes provided access to these data, the collection of which was funded by NSF DDIG #0925793 and Wenner Gren Foundation #8102. The files were downloaded from www.MorphoSource.org, Duke University.
MCZ-31724	doi:10.17602/M2/M2687	3.27 GB	MCZ 31724 skull	Galago senegalensis	0.02847 mm	0.02847 mm	0.02847 mm	80 kv	125 A	10 W	1000	MCZ - CC BY-NC	Citation: Lynn Lucas and Lynn Copes provided access to these data, the collection of which was funded by NSF DDIG #0925793 and Wenner Gren Foundation #8102. The files were downloaded from www.MorphoSource.org, Duke University.
MCZ-31939	doi:10.17602/M2/M2901	1.83 GB	MCZ 31939 skull	Colobus badius	0.079977 mm	0.079977 mm	0.079977 mm	80 kv	125 A	10 W	1100	MCZ - CC BY-NC	Citation: Lynn Lucas and Lynn Copes provided access to these data, the collection of which was funded by NSF DDIG #0925793 and Wenner Gren Foundation #8102. The files were downloaded from www.MorphoSource.org, Duke University.
MCZ-31949	doi:10.17602/M2/M4727	3.15 GB	MCZ 31949 cranium	Papio doguera ibeanus	0.110151 mm	0.110151 mm	0.110151 mm	90 kv	125 A	11.3 W	1100	MCZ - CC BY-NC	Citation: Lynn Lucas and Lynn Copes provided access to these data, the collection of which was funded by NSF DDIG #0925793 and Wenner Gren Foundation #8102. The files were downloaded from www.MorphoSource.org, Duke University.
MCZ-31949	doi:10.17602/M2/M4729	4.56 MB	MCZ 31949 mandible	Papio doguera ibeanus	0.08453 mm	0.08453 mm	0.08453 mm	90 kv	125 A	11.3 W	1100	MCZ - CC BY-NC	Citation: Lynn Lucas and Lynn Copes provided access to these data, the collection of which was funded by NSF DDIG #0925793 and Wenner Gren Foundation #8102. The files were downloaded from www.MorphoSource.org, Duke University.
MCZ-31986	doi:10.17602/M2/M2931	1.14 GB	MCZ 31986 skull	Cercopithecus mitis	0.079988 mm	0.079988 mm	0.079988 mm	80 kv	125 A	10 W	1100	MCZ - CC BY-NC	Citation: Lynn Lucas and Lynn Copes provided access to these data, the collection of which was funded by NSF DDIG #0925793 and Wenner Gren Foundation #8102. The files were downloaded from www.MorphoSource.org, Duke University.
MCZ-32003	doi:10.17602/M2/M2932	2.31 GB	MCZ 32003 skull	Cercopithecus mitis	0.079988 mm	0.079988 mm	0.079988 mm	80 kv	125 A	10 W	1100	MCZ - CC BY-NC	Citation: Lynn Lucas and Lynn Copes provided access to these data, the collection of which was funded by NSF DDIG #0925793 and Wenner Gren Foundation #8102. The files were downloaded from www.MorphoSource.org, Duke University.
MCZ-32164	doi:10.17602/M2/M5196	1.02 GB	MCZ 32164 skull	Callithrix argentata	0.040724 mm	0.040724 mm	0.040724 mm	80 kv	125 A	10 W	1000	MCZ - CC BY-NC	Citation: Lynn Lucas and Lynn Copes provided access to these data, the collection of which was funded by NSF DDIG #0925793 and Wenner Gren Foundation #8102. The files were downloaded from www.MorphoSource.org, Duke University.
MCZ-32165	doi:10.17602/M2/M5197	1.16 GB	MCZ 32165 skull	Callithrix argentata	0.040724 mm	0.040724 mm	0.040724 mm	80 kv	125 A	10 W	1000	MCZ - CC BY-NC	Citation: Lynn Lucas and Lynn Copes provided access to these data, the collection of which was funded by NSF DDIG #0925793 and Wenner Gren Foundation #8102. The files were downloaded from www.MorphoSource.org, Duke University.
MCZ-32380	doi:10.17602/M2/M5169	1.83 GB	MCZ 32380 skull	Callicebus moloch	0.047097 mm	0.047097 mm	0.047097 mm	80 kv	120 A	9.6 W	1050	MCZ - CC BY-NC	Citation: Lynn Lucas and Lynn Copes provided access to these data, the collection of which was funded by NSF DDIG #0925793 and Wenner Gren Foundation #8102. The files were downloaded from www.MorphoSource.org, Duke University.
MCZ-32383	doi:10.17602/M2/M5170	1.61 GB	MCZ 32383 skull	Callicebus moloch	0.047097 mm	0.047097 mm	0.047097 mm	80 kv	120 A	9.6 W	1050	MCZ - CC BY-NC	Citation: Lynn Lucas and Lynn Copes provided access to these data, the collection of which was funded by NSF DDIG #0925793 and Wenner Gren Foundation #8102. The files were downloaded from www.MorphoSource.org, Duke University.
MCZ-32503	doi:10.17602/M2/M2730	5.03 GB	MCZ 32503 skull	Avahi laniger	0.030704 mm	0.030704 mm	0.030704 mm	80 kv	125 A	10 W	1000	MCZ - CC BY-NC	Citation: Lynn Lucas and Lynn Copes provided access to these data, the collection of which was funded by NSF DDIG #0925793 and Wenner Gren Foundation #8102. The files were downloaded from www.MorphoSource.org, Duke University.
MCZ-32624	doi:10.17602/M2/M2590	3.26 GB	MCZ 32624 skull	Cercocebus torquatus	0.092963 mm	0.092963 mm	0.092963 mm	85 kv	125 A	10.6 W	1100	MCZ - CC BY-NC	Citation: Lynn Lucas and Lynn Copes provided access to these data, the collection of which was funded by NSF DDIG #0925793 and Wenner Gren Foundation #8102. The files were downloaded from www.MorphoSource.org, Duke University.
MCZ-32625	doi:10.17602/M2/M2589	3.6 GB	MCZ 32625 skull	Cercocebus torquatus	0.090751 mm	0.090751 mm	0.090751 mm	85 kv	125 A	10.6 W	1100	MCZ - CC BY-NC	Citation: Lynn Lucas and Lynn Copes provided access to these data, the collection of which was funded by NSF DDIG #0925793 and Wenner Gren Foundation #8102. The files were downloaded from www.MorphoSource.org, Duke University.
MCZ-33908	doi:10.17602/M2/M2716	3.66 GB	MCZ 33908 skull	Galago senegalensis	0.025449 mm	0.025449 mm	0.025449 mm	80 kv	125 A	10 W	1000	MCZ - CC BY-NC	Citation: Lynn Lucas and Lynn Copes provided access to these data, the collection of which was funded by NSF DDIG #0925793 and Wenner Gren Foundation #8102. The files were downloaded from www.MorphoSource.org, Duke University.
MCZ-34089	doi:10.17602/M2/M5096	2.06 GB	MCZ 34089 cranium	Mandrillus sphinx	0.125974 mm	0.125974 mm	0.125974 mm	85 kv	125 A	10 W	1100	MCZ - CC BY-NC	Citation: Lynn Lucas and Lynn Copes provided access to these data, the collection of which was funded by NSF DDIG #0925793 and Wenner Gren Foundation #8102. The files were downloaded from www.MorphoSource.org, Duke University.
MCZ-34089	doi:10.17602/M2/M5097	2.89 GB	MCZ 34089 mandible	Mandrillus sphinx	0.090718 mm	0.090718 mm	0.090718 mm	85 kv	125 A	10 W	1000	MCZ - CC BY-NC	Citation: Lynn Lucas and Lynn Copes provided access to these data, the collection of which was funded by NSF DDIG #0925793 and Wenner Gren Foundation #8102. The files were downloaded from www.MorphoSource.org, Duke University.
MCZ-34177	doi:10.17602/M2/M5100	3.05 GB	MCZ 334177 skull	Mandrillus sphinx	0.090718 mm	0.090718 mm	0.090718 mm	85 kv	125 A	10 W	1100	MCZ - CC BY-NC	Citation: Lynn Lucas and Lynn Copes provided access to these data, the collection of which was funded by NSF DDIG #0925793 and Wenner Gren Foundation #8102. The files were downloaded from www.MorphoSource.org, Duke University.
MCZ-34264	doi:10.17602/M2/M5092	2.45 GB	MCZ 34264 skull	Miopithecus talapoin	0.050092 mm	0.050092 mm	0.050092 mm	80 kv	125 A	10 W	1000	MCZ - CC BY-NC	Citation: Lynn Lucas and Lynn Copes provided access to these data, the collection of which was funded by NSF DDIG #0925793 and Wenner Gren Foundation #8102. The files were downloaded from www.MorphoSource.org, Duke University.
MCZ-34272	doi:10.17602/M2/M5099	3.46 MB	MCZ 34272 skull	Mandrillus sphinx	0.090916 mm	0.090916 mm	0.090916 mm	85 kv	125 A	10 W	1100	MCZ - CC BY-NC	Citation: Lynn Lucas and Lynn Copes provided access to these data, the collection of which was funded by NSF DDIG #0925793 and Wenner Gren Foundation #8102. The files were downloaded from www.MorphoSource.org, Duke University.
MCZ-34273	doi:10.17602/M2/M5098	2.86 GB	MCZ 34273 skull	Mandrillus sphinx	0.07286 mm	0.07286 mm	0.07286 mm	85 kv	125 A	10 W	1100	MCZ - CC BY-NC	Citation: Lynn Lucas and Lynn Copes provided access to these data, the collection of which was funded by NSF DDIG #0925793 and Wenner Gren Foundation #8102. The files were downloaded from www.MorphoSource.org, Duke University.
MCZ-34322	doi:10.17602/M2/M2921	3.66 GB	MCZ 34322 skull	Ateles geoffroyi	0.063468 mm	0.063468 mm	0.063468 mm	90 kv	120 A	10.8 W	1100	MCZ - CC BY-NC	Citation: Lynn Lucas and Lynn Copes provided access to these data, the collection of which was funded by NSF DDIG #0925793 and Wenner Gren Foundation #8102. The files were downloaded from www.MorphoSource.org, Duke University.
MCZ-34323	doi:10.17602/M2/M5149	1.14 GB	MCZ 34323 skull	Cebus capucinus	0.079988 mm	0.079988 mm	0.079988 mm	80 kv	115 A	9.2 W	1100	MCZ - CC BY-NC	Citation: Lynn Lucas and Lynn Copes provided access to these data, the collection of which was funded by NSF DDIG #0925793 and Wenner Gren Foundation #8102. The files were downloaded from www.MorphoSource.org, Duke University.
MCZ-34326	doi:10.17602/M2/M5148	1.67 GB	MCZ 34326 skull	Cebus capucinus	0.079988 mm	0.079988 mm	0.079988 mm	80 kv	115 A	9.2 W	1100	MCZ - CC BY-NC	Citation: Lynn Lucas and Lynn Copes provided access to these data, the collection of which was funded by NSF DDIG #0925793 and Wenner Gren Foundation #8102. The files were downloaded from www.MorphoSource.org, Duke University.
MCZ-34353	doi:10.17602/M2/M5147	1.39 GB	MCZ 34353 skull	Cebus capucinus	0.079988 mm	0.079988 mm	0.079988 mm	80 kv	115 A	9.2 W	1100	MCZ - CC BY-NC	Citation: Lynn Lucas and Lynn Copes provided access to these data, the collection of which was funded by NSF DDIG #0925793 and Wenner Gren Foundation #8102. The files were downloaded from www.MorphoSource.org, Duke University.
MCZ-34381	doi:10.17602/M2/M2698	3.15 GB	MCZ 34381 skull	Galago senegalensis	0.024913 mm	0.024913 mm	0.024913 mm	80 kv	125 A	10 W	1000	MCZ - CC BY-NC	Citation: Lynn Lucas and Lynn Copes provided access to these data, the collection of which was funded by NSF DDIG #0925793 and Wenner Gren Foundation #8102. The files were downloaded from www.MorphoSource.org, Duke University.
MCZ-34573	doi:10.17602/M2/M5198	1.18 GB	MCZ 34573 skull	Callithrix argentata	0.040724 mm	0.040724 mm	0.040724 mm	80 kv	125 A	10 W	1000	MCZ - CC BY-NC	Citation: Lynn Lucas and Lynn Copes provided access to these data, the collection of which was funded by NSF DDIG #0925793 and Wenner Gren Foundation #8102. The files were downloaded from www.MorphoSource.org, Duke University.
MCZ-34917	doi:10.17602/M2/M2699	2.62 GB	MCZ 34917 skull	Galago senegalensis	0.02418 mm	0.02418 mm	0.02418 mm	80 kv	125 A	10 W	1000	MCZ - CC BY-NC	Citation: Lynn Lucas and Lynn Copes provided access to these data, the collection of which was funded by NSF DDIG #0925793 and Wenner Gren Foundation #8102. The files were downloaded from www.MorphoSource.org, Duke University.
MCZ-35058	doi:10.17602/M2/M3032	3.5 GB	MCZ 35058 skull	Macaca fascicularis	0.061559 mm	0.061559 mm	0.061559 mm	80 kv	125 A	10 W	1050	MCZ - CC BY-NC	Citation: Lynn Lucas and Lynn Copes provided access to these data, the collection of which was funded by NSF DDIG #0925793 and Wenner Gren Foundation #8102. The files were downloaded from www.MorphoSource.org, Duke University.
MCZ-35567	doi:10.17602/M2/M4439	5.72 GB	MCZ 35567 skull	Trachypithecus cristata	0.050091 mm	0.050091 mm	0.050091 mm	90 kv	120 A	10.8 W	1000	MCZ - CC BY-NC	Citation: Lynn Lucas and Lynn Copes provided access to these data, the collection of which was funded by NSF DDIG #0925793 and Wenner Gren Foundation #8102. The files were downloaded from www.MorphoSource.org, Duke University.
MCZ-35584	doi:10.17602/M2/M4437	978.23 MB	MCZ 35584 humerus and femur	Trachypithecus cristata	0.096918 mm	0.096918 mm	0.096918 mm	90 kv	125 A	11.3 W	800	MCZ - CC BY-NC	Citation: Lynn Lucas and Lynn Copes provided access to these data, the collection of which was funded by NSF DDIG #0925793 and Wenner Gren Foundation #8102. The files were downloaded from www.MorphoSource.org, Duke University.
MCZ-35584	doi:10.17602/M2/M4436	5.7 GB	MCZ 35584 skull	Trachypithecus cristata	0.050998 mm	0.050998 mm	0.050998 mm	90 kv	120 A	10.8 W	1000	MCZ - CC BY-NC	Citation: Lynn Lucas and Lynn Copes provided access to these data, the collection of which was funded by NSF DDIG #0925793 and Wenner Gren Foundation #8102. The files were downloaded from www.MorphoSource.org, Duke University.
MCZ-35586	doi:10.17602/M2/M4435	5.94 GB	MCZ 35586 skull	Trachypithecus cristata	0.050092 mm	0.050092 mm	0.050092 mm	90 kv	120 A	10.8 W	1000	MCZ - CC BY-NC	Citation: Lynn Lucas and Lynn Copes provided access to these data, the collection of which was funded by NSF DDIG #0925793 and Wenner Gren Foundation #8102. The files were downloaded from www.MorphoSource.org, Duke University.
MCZ-35597	doi:10.17602/M2/M4434	987.12 MB	MCZ 35597 humerus and femur	Trachypithecus cristata	0.09193 mm	0.09193 mm	0.09193 mm	90 kv	125 A	11.3 W	800	MCZ - CC BY-NC	Citation: Lynn Lucas and Lynn Copes provided access to these data, the collection of which was funded by NSF DDIG #0925793 and Wenner Gren Foundation #8102. The files were downloaded from www.MorphoSource.org, Duke University.
MCZ-35597	doi:10.17602/M2/M4433	5.06 GB	MCZ 35597 skull	Trachypithecus cristata	0.050091 mm	0.050091 mm	0.050091 mm	90 kv	120 A	10.8 W	1000	MCZ - CC BY-NC	Citation: Lynn Lucas and Lynn Copes provided access to these data, the collection of which was funded by NSF DDIG #0925793 and Wenner Gren Foundation #8102. The files were downloaded from www.MorphoSource.org, Duke University.
MCZ-35603	doi:10.17602/M2/M4432	1.06 GB	MCZ 35603 humerus and femur	Trachypithecus cristata	0.091226 mm	0.091226 mm	0.091226 mm	90 kv	125 A	11.3 W	800	MCZ - CC BY-NC	Citation: Lynn Lucas and Lynn Copes provided access to these data, the collection of which was funded by NSF DDIG #0925793 and Wenner Gren Foundation #8102. The files were downloaded from www.MorphoSource.org, Duke University.
MCZ-35603	doi:10.17602/M2/M4431	5.56 GB	MCZ 35603 skull	Trachypithecus cristata	0.050093 mm	0.050093 mm	0.050093 mm	90 kv	120 A	10.8 W	1000	MCZ - CC BY-NC	Citation: Lynn Lucas and Lynn Copes provided access to these data, the collection of which was funded by NSF DDIG #0925793 and Wenner Gren Foundation #8102. The files were downloaded from www.MorphoSource.org, Duke University.
MCZ-35604	doi:10.17602/M2/M4430	1.06 GB	MCZ 35604 humerus and femur	Trachypithecus cristata	0.092316 mm	0.092316 mm	0.092316 mm	90 kv	125 A	11.3 W	800	MCZ - CC BY-NC	Citation: Lynn Lucas and Lynn Copes provided access to these data, the collection of which was funded by NSF DDIG #0925793 and Wenner Gren Foundation #8102. The files were downloaded from www.MorphoSource.org, Duke University.
MCZ-35604	doi:10.17602/M2/M4429	5.84 GB	MCZ 35604 skull	Trachypithecus cristata	0.050092 mm	0.050092 mm	0.050092 mm	90 kv	120 A	10.8 W	1000	MCZ - CC BY-NC	Citation: Lynn Lucas and Lynn Copes provided access to these data, the collection of which was funded by NSF DDIG #0925793 and Wenner Gren Foundation #8102. The files were downloaded from www.MorphoSource.org, Duke University.
MCZ-35605	doi:10.17602/M2/M4428	857.16 MB	MCZ 35605 humerus and femur	Trachypithecus cristata	0.096791 mm	0.096791 mm	0.096791 mm	90 kv	125 A	11.3 W	800	MCZ - CC BY-NC	Citation: Lynn Lucas and Lynn Copes provided access to these data, the collection of which was funded by NSF DDIG #0925793 and Wenner Gren Foundation #8102. The files were downloaded from www.MorphoSource.org, Duke University.
MCZ-35605	doi:10.17602/M2/M4427	4.15 GB	MCZ 35605 skull	Trachypithecus cristata	0.050092 mm	0.050092 mm	0.050092 mm	90 kv	120 A	10.8 W	1000	MCZ - CC BY-NC	Citation: Lynn Lucas and Lynn Copes provided access to these data, the collection of which was funded by NSF DDIG #0925793 and Wenner Gren Foundation #8102. The files were downloaded from www.MorphoSource.org, Duke University.
MCZ-35610	doi:10.17602/M2/M4425	1.01 GB	MCZ 35610 humerus and femur	Trachypithecus cristata	0.089344 mm	0.089344 mm	0.089344 mm	90 kv	125 A	11.3 W	800	MCZ - CC BY-NC	Citation: Lynn Lucas and Lynn Copes provided access to these data, the collection of which was funded by NSF DDIG #0925793 and Wenner Gren Foundation #8102. The files were downloaded from www.MorphoSource.org, Duke University.
MCZ-35610	doi:10.17602/M2/M4424	3.54 GB	MCZ 35610 skull	Trachypithecus cristata	0.050092 mm	0.050092 mm	0.050092 mm	90 kv	120 A	10.8 W	1000	MCZ - CC BY-NC	Citation: Lynn Lucas and Lynn Copes provided access to these data, the collection of which was funded by NSF DDIG #0925793 and Wenner Gren Foundation #8102. The files were downloaded from www.MorphoSource.org, Duke University.
MCZ-35615	doi:10.17602/M2/M4426	4.15 GB	MCZ 35615 skull	Trachypithecus cristata	0.050092 mm	0.050092 mm	0.050092 mm	80 kv	110 A	8.8 W	1000	MCZ - CC BY-NC	Citation: Lynn Lucas and Lynn Copes provided access to these data, the collection of which was funded by NSF DDIG #0925793 and Wenner Gren Foundation #8102. The files were downloaded from www.MorphoSource.org, Duke University.
MCZ-35618	doi:10.17602/M2/M4423	1.08 GB	MCZ 35618 humerus and femur	Trachypithecus cristata	0.089344 mm	0.089344 mm	0.089344 mm	90 kv	125 A	11.3 W	800	MCZ - CC BY-NC	Citation: Lynn Lucas and Lynn Copes provided access to these data, the collection of which was funded by NSF DDIG #0925793 and Wenner Gren Foundation #8102. The files were downloaded from www.MorphoSource.org, Duke University.
MCZ-35618	doi:10.17602/M2/M4422	4.46 GB	MCZ 35618 skull	Trachypithecus cristata	0.058938 mm	0.058938 mm	0.058938 mm	80 kv	110 A	8.8 W	1000	MCZ - CC BY-NC	Citation: Lynn Lucas and Lynn Copes provided access to these data, the collection of which was funded by NSF DDIG #0925793 and Wenner Gren Foundation #8102. The files were downloaded from www.MorphoSource.org, Duke University.
MCZ-35621	doi:10.17602/M2/M4611	1.24 GB	MCZ 35621 skull	Presbytis hosei	0.079977 mm	0.079977 mm	0.079977 mm	80 kv	125 A	10 W	1100	MCZ - CC BY-NC	Citation: Lynn Lucas and Lynn Copes provided access to these data, the collection of which was funded by NSF DDIG #0925793 and Wenner Gren Foundation #8102. The files were downloaded from www.MorphoSource.org, Duke University.
MCZ-35636	doi:10.17602/M2/M4421	774.26 MB	MCZ 35636 humerus and femur	Trachypithecus cristata	0.089587 mm	0.089587 mm	0.089587 mm	90 kv	125 A	11.3 W	800	MCZ - CC BY-NC	Citation: Lynn Lucas and Lynn Copes provided access to these data, the collection of which was funded by NSF DDIG #0925793 and Wenner Gren Foundation #8102. The files were downloaded from www.MorphoSource.org, Duke University.
MCZ-35636	doi:10.17602/M2/M4420	2.26 GB	MCZ 35636 skull	Trachypithecus cristata	0.06458 mm	0.06458 mm	0.06458 mm	80 kv	110 A	8.8 W	1000	MCZ - CC BY-NC	Citation: Lynn Lucas and Lynn Copes provided access to these data, the collection of which was funded by NSF DDIG #0925793 and Wenner Gren Foundation #8102. The files were downloaded from www.MorphoSource.org, Duke University.
MCZ-35640	doi:10.17602/M2/M4418	776.19 MB	MCZ 35640 humerus and femur	Trachypithecus cristata	0.09226 mm	0.09226 mm	0.09226 mm	90 kv	125 A	11.3 W	800	MCZ - CC BY-NC	Citation: Lynn Lucas and Lynn Copes provided access to these data, the collection of which was funded by NSF DDIG #0925793 and Wenner Gren Foundation #8102. The files were downloaded from www.MorphoSource.org, Duke University.
MCZ-35640	doi:10.17602/M2/M4417	4.7 GB	MCZ 35640 skull	Trachypithecus cristata	0.055693 mm	0.055693 mm	0.055693 mm	80 kv	110 A	8.8 W	1000	MCZ - CC BY-NC	Citation: Lynn Lucas and Lynn Copes provided access to these data, the collection of which was funded by NSF DDIG #0925793 and Wenner Gren Foundation #8102. The files were downloaded from www.MorphoSource.org, Duke University.
MCZ-35645	doi:10.17602/M2/M4419	3.03 GB	MCZ 35645 skull	Trachypithecus cristata	0.050092 mm	0.050092 mm	0.050092 mm	80 kv	110 A	8.8 W	1000	MCZ - CC BY-NC	Citation: Lynn Lucas and Lynn Copes provided access to these data, the collection of which was funded by NSF DDIG #0925793 and Wenner Gren Foundation #8102. The files were downloaded from www.MorphoSource.org, Duke University.
MCZ-35663	doi:10.17602/M2/M4416	954.15 MB	MCZ 35663 humerus and femur	Trachypithecus cristata	0.08752 mm	0.08752 mm	0.08752 mm	90 kv	125 A	11.3 W	800	MCZ - CC BY-NC	Citation: Lynn Lucas and Lynn Copes provided access to these data, the collection of which was funded by NSF DDIG #0925793 and Wenner Gren Foundation #8102. The files were downloaded from www.MorphoSource.org, Duke University.
MCZ-35663	doi:10.17602/M2/M4415	4.42 GB	MCZ 35663 skull	Trachypithecus cristata	0.055693 mm	0.055693 mm	0.055693 mm	80 kv	110 A	8.8 W	1000	MCZ - CC BY-NC	Citation: Lynn Lucas and Lynn Copes provided access to these data, the collection of which was funded by NSF DDIG #0925793 and Wenner Gren Foundation #8102. The files were downloaded from www.MorphoSource.org, Duke University.
MCZ-35678	doi:10.17602/M2/M4414	828.58 MB	MCZ 35678 humerus and femur	Trachypithecus cristata	0.092769 mm	0.092769 mm	0.092769 mm	90 kv	125 A	11.3 W	800	MCZ - CC BY-NC	Citation: Lynn Lucas and Lynn Copes provided access to these data, the collection of which was funded by NSF DDIG #0925793 and Wenner Gren Foundation #8102. The files were downloaded from www.MorphoSource.org, Duke University.
MCZ-35678	doi:10.17602/M2/M4413	3.93 GB	MCZ 35678 skull	Trachypithecus cristata	0.058739 mm	0.058739 mm	0.058739 mm	85 kv	120 A	10.2 W	1050	MCZ - CC BY-NC	Citation: Lynn Lucas and Lynn Copes provided access to these data, the collection of which was funded by NSF DDIG #0925793 and Wenner Gren Foundation #8102. The files were downloaded from www.MorphoSource.org, Duke University.
MCZ-35682	doi:10.17602/M2/M4412	796.19 MB	MCZ 35682 humerus and femur	Trachypithecus cristata	0.093436 mm	0.093436 mm	0.093436 mm	90 kv	125 A	11.3 W	800	MCZ - CC BY-NC	Citation: Lynn Lucas and Lynn Copes provided access to these data, the collection of which was funded by NSF DDIG #0925793 and Wenner Gren Foundation #8102. The files were downloaded from www.MorphoSource.org, Duke University.
MCZ-35682	doi:10.17602/M2/M4411	2.34 GB	MCZ 35682 skull	Trachypithecus cristata	0.065843 mm	0.065843 mm	0.065843 mm	80 kv	110 A	8.8 W	1100	MCZ - CC BY-NC	Citation: Lynn Lucas and Lynn Copes provided access to these data, the collection of which was funded by NSF DDIG #0925793 and Wenner Gren Foundation #8102. The files were downloaded from www.MorphoSource.org, Duke University.
MCZ-35683	doi:10.17602/M2/M4410	826.17 MB	MCZ 35683 humerus and femur	Trachypithecus cristata	0.096922 mm	0.096922 mm	0.096922 mm	90 kv	125 A	11.3 W	800	MCZ - CC BY-NC	Citation: Lynn Lucas and Lynn Copes provided access to these data, the collection of which was funded by NSF DDIG #0925793 and Wenner Gren Foundation #8102. The files were downloaded from www.MorphoSource.org, Duke University.
MCZ-35683	doi:10.17602/M2/M4409	4.92 GB	MCZ 35683 skull	Trachypithecus cristata	0.055693 mm	0.055693 mm	0.055693 mm	80 kv	110 A	8.8 W	1000	MCZ - CC BY-NC	Citation: Lynn Lucas and Lynn Copes provided access to these data, the collection of which was funded by NSF DDIG #0925793 and Wenner Gren Foundation #8102. The files were downloaded from www.MorphoSource.org, Duke University.
MCZ-35696	doi:10.17602/M2/M4408	950.07 MB	MCZ 35696 humerus and femur	Trachypithecus cristata	0.095287 mm	0.095287 mm	0.095287 mm	90 kv	125 A	11.3 W	800	MCZ - CC BY-NC	Citation: Lynn Lucas and Lynn Copes provided access to these data, the collection of which was funded by NSF DDIG #0925793 and Wenner Gren Foundation #8102. The files were downloaded from www.MorphoSource.org, Duke University.
MCZ-35696	doi:10.17602/M2/M4407	3.69 GB	MCZ 35696 skull	Trachypithecus cristata	0.062643 mm	0.062643 mm	0.062643 mm	80 kv	110 A	8.8 W	1000	MCZ - CC BY-NC	Citation: Lynn Lucas and Lynn Copes provided access to these data, the collection of which was funded by NSF DDIG #0925793 and Wenner Gren Foundation #8102. The files were downloaded from www.MorphoSource.org, Duke University.
MCZ-35704	doi:10.17602/M2/M4553	681.33 MB	MCZ 35704 humerus and femur	Trachypithecus cristata	0.105008 mm	0.105008 mm	0.105008 mm	90 kv	125 A	11.3 W	800	MCZ - CC BY-NC	Citation: Lynn Lucas and Lynn Copes provided access to these data, the collection of which was funded by NSF DDIG #0925793 and Wenner Gren Foundation #8102. The files were downloaded from www.MorphoSource.org, Duke University.
MCZ-35704	doi:10.17602/M2/M4552	1.16 GB	MCZ 35704 skull	Trachypithecus cristata	0.079977 mm	0.079977 mm	0.079977 mm	80 kv	125 A	10 W	1100	MCZ - CC BY-NC	Citation: Lynn Lucas and Lynn Copes provided access to these data, the collection of which was funded by NSF DDIG #0925793 and Wenner Gren Foundation #8102. The files were downloaded from www.MorphoSource.org, Duke University.
MCZ-35705	doi:10.17602/M2/M4551	657.51 MB	MCZ 35705 humerus and femur	Presbytis rubicunda	0.105095 mm	0.105095 mm	0.105095 mm	90 kv	125 A	11.3 W	800	MCZ - CC BY-NC	Citation: Lynn Lucas and Lynn Copes provided access to these data, the collection of which was funded by NSF DDIG #0925793 and Wenner Gren Foundation #8102. The files were downloaded from www.MorphoSource.org, Duke University.
MCZ-35705	doi:10.17602/M2/M4550	1.73 GB	MCZ 35705 skull	Presbytis rubicunda	0.079977 mm	0.079977 mm	0.079977 mm	80 kv	125 A	10 W	1100	MCZ - CC BY-NC	Citation: Lynn Lucas and Lynn Copes provided access to these data, the collection of which was funded by NSF DDIG #0925793 and Wenner Gren Foundation #8102. The files were downloaded from www.MorphoSource.org, Duke University.
MCZ-35706	doi:10.17602/M2/M4556	1.44 GB	MCZ 35706 skull	Presbytis rubicunda	0.079977 mm	0.079977 mm	0.079977 mm	80 kv	125 A	10 W	1100	MCZ - CC BY-NC	Citation: Lynn Lucas and Lynn Copes provided access to these data, the collection of which was funded by NSF DDIG #0925793 and Wenner Gren Foundation #8102. The files were downloaded from www.MorphoSource.org, Duke University.
MCZ-35712	doi:10.17602/M2/M4555	1.3 GB	MCZ 35712 skull	Presbytis rubicunda	0.079977 mm	0.079977 mm	0.079977 mm	80 kv	125 A	10 W	1100	MCZ - CC BY-NC	Citation: Lynn Lucas and Lynn Copes provided access to these data, the collection of which was funded by NSF DDIG #0925793 and Wenner Gren Foundation #8102. The files were downloaded from www.MorphoSource.org, Duke University.
MCZ-35718	doi:10.17602/M2/M4406	998.88 MB	MCZ 35718 humerus and femur	Trachypithecus cristata	0.090914 mm	0.090914 mm	0.090914 mm	90 kv	125 A	11.3 W	800	MCZ - CC BY-NC	Citation: Lynn Lucas and Lynn Copes provided access to these data, the collection of which was funded by NSF DDIG #0925793 and Wenner Gren Foundation #8102. The files were downloaded from www.MorphoSource.org, Duke University.
MCZ-35718	doi:10.17602/M2/M4405	4.01 GB	MCZ 35718 skull	Trachypithecus cristata	0.055693 mm	0.055693 mm	0.055693 mm	80 kv	110 A	8.8 W	1000	MCZ - CC BY-NC	Citation: Lynn Lucas and Lynn Copes provided access to these data, the collection of which was funded by NSF DDIG #0925793 and Wenner Gren Foundation #8102. The files were downloaded from www.MorphoSource.org, Duke University.
MCZ-35719	doi:10.17602/M2/M4615	3.09 GB	MCZ 35719 cranium	Pongo pygmaeus	0.102556 mm	0.102556 mm	0.102556 mm	85 kv	90 A	7.7 W	1500	MCZ - CC BY-NC	Citation: Lynn Lucas and Lynn Copes provided access to these data, the collection of which was funded by NSF DDIG #0925793 and Wenner Gren Foundation #8102. The files were downloaded from www.MorphoSource.org, Duke University.
MCZ-35719	doi:10.17602/M2/M4616	3.9 GB	MCZ 35719 mandible	Pongo pygmaeus	0.083294 mm	0.083294 mm	0.083294 mm	85 kv	90 A	7.7 W	1000	MCZ - CC BY-NC	Citation: Lynn Lucas and Lynn Copes provided access to these data, the collection of which was funded by NSF DDIG #0925793 and Wenner Gren Foundation #8102. The files were downloaded from www.MorphoSource.org, Duke University.
MCZ-35765	doi:10.17602/M2/M3033	2.61 GB	MCZ 35765 skull	Macaca fascicularis	0.061559 mm	0.061559 mm	0.061559 mm	9- kv	125 A	10 W	1050	MCZ - CC BY-NC	Citation: Lynn Lucas and Lynn Copes provided access to these data, the collection of which was funded by NSF DDIG #0925793 and Wenner Gren Foundation #8102. The files were downloaded from www.MorphoSource.org, Duke University.
MCZ-35937	doi:10.17602/M2/M3034	3.69 GB	MCZ 35937 skull	Macaca fascicularis	0.061559 mm	0.061559 mm	0.061559 mm	80 kv	125 A	10 W	1050	MCZ - CC BY-NC	Citation: Lynn Lucas and Lynn Copes provided access to these data, the collection of which was funded by NSF DDIG #0925793 and Wenner Gren Foundation #8102. The files were downloaded from www.MorphoSource.org, Duke University.
MCZ-35938	doi:10.17602/M2/M3035	3.32 GB	MCZ 35938 skull	Macaca fascicularis	0.061559 mm	0.061559 mm	0.061559 mm	80 kv	125 A	10 W	1050	MCZ - CC BY-NC	Citation: Lynn Lucas and Lynn Copes provided access to these data, the collection of which was funded by NSF DDIG #0925793 and Wenner Gren Foundation #8102. The files were downloaded from www.MorphoSource.org, Duke University.
MCZ-36030	doi:10.17602/M2/M3036	3 GB	MCZ 36030 skull	Macaca fascicularis	0.061559 mm	0.061559 mm	0.061559 mm	80 kv	125 A	10 W	1050	MCZ - CC BY-NC	Citation: Lynn Lucas and Lynn Copes provided access to these data, the collection of which was funded by NSF DDIG #0925793 and Wenner Gren Foundation #8102. The files were downloaded from www.MorphoSource.org, Duke University.
MCZ-36031	doi:10.17602/M2/M4444	3.49 GB	MCZ 36031 skull	Symphalangus syndactylus	0.075369 mm	0.075369 mm	0.075369 mm	85 kv	125 A	10 W	1100	MCZ - CC BY-NC	Citation: Lynn Lucas and Lynn Copes provided access to these data, the collection of which was funded by NSF DDIG #0925793 and Wenner Gren Foundation #8102. The files were downloaded from www.MorphoSource.org, Duke University.
MCZ-36032	doi:10.17602/M2/M4443	2.92 GB	MCZ 36032 skull	Symphalangus syndactylus	0.074155 mm	0.074155 mm	0.074155 mm	85 kv	125 A	10 W	1100	MCZ - CC BY-NC	Citation: Lynn Lucas and Lynn Copes provided access to these data, the collection of which was funded by NSF DDIG #0925793 and Wenner Gren Foundation #8102. The files were downloaded from www.MorphoSource.org, Duke University.
MCZ-36035	doi:10.17602/M2/M2719	4.42 GB	MCZ 36035 skull	Nycticebus pygmaeus	0.030826 mm	0.030826 mm	0.030826 mm	80 kv	125 A	10 W	1000	MCZ - CC BY-NC	Citation: Lynn Lucas and Lynn Copes provided access to these data, the collection of which was funded by NSF DDIG #0925793 and Wenner Gren Foundation #8102. The files were downloaded from www.MorphoSource.org, Duke University.
MCZ-36040	doi:10.17602/M2/M2720	5.46 GB	MCZ 36040 skull	Nycticebus coucang	0.030931 mm	0.030931 mm	0.030931 mm	80 kv	125 A	10 W	1000	MCZ - CC BY-NC	Citation: Lynn Lucas and Lynn Copes provided access to these data, the collection of which was funded by NSF DDIG #0925793 and Wenner Gren Foundation #8102. The files were downloaded from www.MorphoSource.org, Duke University.
MCZ-36041	doi:10.17602/M2/M2733	5.92 GB	MCZ 36041 skull	Nycticebus coucang	0.02898 mm	0.02898 mm	0.02898 mm	80 kv	125 A	10 W	1000	MCZ - CC BY-NC	Citation: Lynn Lucas and Lynn Copes provided access to these data, the collection of which was funded by NSF DDIG #0925793 and Wenner Gren Foundation #8102. The files were downloaded from www.MorphoSource.org, Duke University.
MCZ-36116	doi:10.17602/M2/M2721	6.21 GB	MCZ 36116 skull	Nycticebus coucang	0.02898 mm	0.02898 mm	0.02898 mm	80 kv	125 A	10 W	1000	MCZ - CC BY-NC	Citation: Lynn Lucas and Lynn Copes provided access to these data, the collection of which was funded by NSF DDIG #0925793 and Wenner Gren Foundation #8102. The files were downloaded from www.MorphoSource.org, Duke University.
MCZ-36820	doi:10.17602/M2/M4554	1.52 GB	MCZ 36820 skull	Presbytis rubicunda	0.079988 mm	0.079988 mm	0.079988 mm	80 kv	120 A	9.6 W	1100	MCZ - CC BY-NC	Citation: Lynn Lucas and Lynn Copes provided access to these data, the collection of which was funded by NSF DDIG #0925793 and Wenner Gren Foundation #8102. The files were downloaded from www.MorphoSource.org, Duke University.
MCZ-37260	doi:10.17602/M2/M4387	4.08 GB	MCZ 37260 cranium	Pan troglodytes	0.099918 mm	0.099918 mm	0.099918 mm	80 kv	120 A	9.6 W	1500	MCZ - CC BY-NC	Citation: Lynn Lucas and Lynn Copes provided access to these data, the collection of which was funded by NSF DDIG #0925793 and Wenner Gren Foundation #8102. The files were downloaded from www.MorphoSource.org, Duke University.
MCZ-37264	doi:10.17602/M2/M2949	4.57 GB	MCZ 37264 skull	Gorilla gorilla gorilla	0.125529 mm	0.125529 mm	0.125529 mm	85 kv	90 A	7.7 W	1500	MCZ - CC BY-NC	Citation: Lynn Lucas and Lynn Copes provided access to these data, the collection of which was funded by NSF DDIG #0925793 and Wenner Gren Foundation #8102. The files were downloaded from www.MorphoSource.org, Duke University.
MCZ-37266	doi:10.17602/M2/M2952	3.4 GB	MCZ 37266 cranium	Gorilla gorilla gorilla	0.121116 mm	0.121116 mm	0.121116 mm	85 kv	90 A	7.7 W	1500	MCZ - CC BY-NC	Citation: Lynn Lucas and Lynn Copes provided access to these data, the collection of which was funded by NSF DDIG #0925793 and Wenner Gren Foundation #8102. The files were downloaded from www.MorphoSource.org, Duke University.
MCZ-37278	doi:10.17602/M2/M5086	3.02 GB	MCZ 37278 skull	Miopithecus talapoin	0.050092 mm	0.050092 mm	0.050092 mm	80 kv	125 A	10 W	1000	MCZ - CC BY-NC	Citation: Lynn Lucas and Lynn Copes provided access to these data, the collection of which was funded by NSF DDIG #0925793 and Wenner Gren Foundation #8102. The files were downloaded from www.MorphoSource.org, Duke University.
MCZ-37280	doi:10.17602/M2/M2922	3.2 GB	MCZ 37280 skull	Erythrocebus patas	0.082531 mm	0.082531 mm	0.082531 mm	80 kv	125 A	10 W	1100	MCZ - CC BY-NC	Citation: Lynn Lucas and Lynn Copes provided access to these data, the collection of which was funded by NSF DDIG #0925793 and Wenner Gren Foundation #8102. The files were downloaded from www.MorphoSource.org, Duke University.
MCZ-37339	doi:10.17602/M2/M5084	2.92 GB	MCZ 37339 skull	Nasalis larvatus	0.070962 mm	0.070962 mm	0.070962 mm	80 kv	110 A	8.8 W	1100	MCZ - CC BY-NC	Citation: Lynn Lucas and Lynn Copes provided access to these data, the collection of which was funded by NSF DDIG #0925793 and Wenner Gren Foundation #8102. The files were downloaded from www.MorphoSource.org, Duke University.
MCZ-37341	doi:10.17602/M2/M5082	2.32 GB	MCZ 37341 skull	Nasalis larvatus	0.070602 mm	0.070602 mm	0.070602 mm	85 kv	125 A	10 W	1000	MCZ - CC BY-NC	Citation: Lynn Lucas and Lynn Copes provided access to these data, the collection of which was funded by NSF DDIG #0925793 and Wenner Gren Foundation #8102. The files were downloaded from www.MorphoSource.org, Duke University.
MCZ-37342	doi:10.17602/M2/M5079	2.98 GB	MCZ 37342 skull	Nasalis larvatus	0.070602 mm	0.070602 mm	0.070602 mm	85 kv	125 A	10 W	1000	MCZ - CC BY-NC	Citation: Lynn Lucas and Lynn Copes provided access to these data, the collection of which was funded by NSF DDIG #0925793 and Wenner Gren Foundation #8102. The files were downloaded from www.MorphoSource.org, Duke University.
MCZ-37343	doi:10.17602/M2/M5074	2.84 GB	MCZ 37343 skull	Nasalis larvatus	0.070602 mm	0.070602 mm	0.070602 mm	85 kv	125 A	10 W	1000	MCZ - CC BY-NC	Citation: Lynn Lucas and Lynn Copes provided access to these data, the collection of which was funded by NSF DDIG #0925793 and Wenner Gren Foundation #8102. The files were downloaded from www.MorphoSource.org, Duke University.
MCZ-37370	doi:10.17602/M2/M4610	1.24 GB	MCZ 37370 skull	Presbytis hosei	0.079977 mm	0.079977 mm	0.079977 mm	80 kv	125 A	10 W	1100	MCZ - CC BY-NC	Citation: Lynn Lucas and Lynn Copes provided access to these data, the collection of which was funded by NSF DDIG #0925793 and Wenner Gren Foundation #8102. The files were downloaded from www.MorphoSource.org, Duke University.
MCZ-37371	doi:10.17602/M2/M4609	1.96 GB	MC 37371 skull	Presbytis hosei	0.079977 mm	0.079977 mm	0.079977 mm	80 kv	125 A	10 W	1100	MCZ - CC BY-NC	Citation: Lynn Lucas and Lynn Copes provided access to these data, the collection of which was funded by NSF DDIG #0925793 and Wenner Gren Foundation #8102. The files were downloaded from www.MorphoSource.org, Duke University.
MCZ-37372	doi:10.17602/M2/M4608	999.04 MB	MCZ 37372 skull	Presbytis hosei	0.079977 mm	0.079977 mm	0.079977 mm	80 kv	125 A	10 W	1100	MCZ - CC BY-NC	Citation: Lynn Lucas and Lynn Copes provided access to these data, the collection of which was funded by NSF DDIG #0925793 and Wenner Gren Foundation #8102. The files were downloaded from www.MorphoSource.org, Duke University.
MCZ-37387	doi:10.17602/M2/M4404	1.67 GB	MCZ 37387 humerus and femur	Trachypithecus cristata	0.096922 mm	0.096922 mm	0.096922 mm	90 kv	125 A	11.3 W	800	MCZ - CC BY-NC	Citation: Lynn Lucas and Lynn Copes provided access to these data, the collection of which was funded by NSF DDIG #0925793 and Wenner Gren Foundation #8102. The files were downloaded from www.MorphoSource.org, Duke University.
MCZ-37387	doi:10.17602/M2/M4403	4.25 GB	MCZ 37387 skull	Trachypithecus cristata	0.055693 mm	0.055693 mm	0.055693 mm	80 kv	110 A	8.8 W	1000	MCZ - CC BY-NC	Citation: Lynn Lucas and Lynn Copes provided access to these data, the collection of which was funded by NSF DDIG #0925793 and Wenner Gren Foundation #8102. The files were downloaded from www.MorphoSource.org, Duke University.
MCZ-37518	doi:10.17602/M2/M4613	2.72 GB	MCZ 37518 cranium	Pongo pygmaeus	0.125529 mm	0.125529 mm	0.125529 mm	85 kv	90 A	7.7 W	1500	MCZ - CC BY-NC	Citation: Lynn Lucas and Lynn Copes provided access to these data, the collection of which was funded by NSF DDIG #0925793 and Wenner Gren Foundation #8102. The files were downloaded from www.MorphoSource.org, Duke University.
MCZ-37518	doi:10.17602/M2/M4614	4.87 GB	MCZ 37518 mandible	Pongo pygmaeus	0.083294 mm	0.083294 mm	0.083294 mm	85 kv	90 A	7.7 W	1000	MCZ - CC BY-NC	Citation: Lynn Lucas and Lynn Copes provided access to these data, the collection of which was funded by NSF DDIG #0925793 and Wenner Gren Foundation #8102. The files were downloaded from www.MorphoSource.org, Duke University.
MCZ-37625	doi:10.17602/M2/M2950	3.66 GB	MCZ 37625 cranium	Gorilla gorilla gorilla	0.116426 mm	0.116426 mm	0.116426 mm	85 kv	90 A	7.7 W	1500	MCZ - CC BY-NC	Citation: Lynn Lucas and Lynn Copes provided access to these data, the collection of which was funded by NSF DDIG #0925793 and Wenner Gren Foundation #8102. The files were downloaded from www.MorphoSource.org, Duke University.
MCZ-37625	doi:10.17602/M2/M2951	4.63 GB	MCZ 37625 mandible	Gorilla gorilla gorilla	0.079977 mm	0.079977 mm	0.079977 mm	85 kv	90 A	7.7 W	1000	MCZ - CC BY-NC	Citation: Lynn Lucas and Lynn Copes provided access to these data, the collection of which was funded by NSF DDIG #0925793 and Wenner Gren Foundation #8102. The files were downloaded from www.MorphoSource.org, Duke University.
MCZ-37666	doi:10.17602/M2/M4549	430.76 MB	MCZ 37666 humerus and femur	Presbytis rubicunda	0.100178 mm	0.100178 mm	0.100178 mm	90 kv	125 A	11.3 W	800	MCZ - CC BY-NC	Citation: Lynn Lucas and Lynn Copes provided access to these data, the collection of which was funded by NSF DDIG #0925793 and Wenner Gren Foundation #8102. The files were downloaded from www.MorphoSource.org, Duke University.
MCZ-37666	doi:10.17602/M2/M4548	1.25 GB	MCZ 37666 skull	Presbytis rubicunda	0.079977 mm	0.079977 mm	0.079977 mm	80 kv	125 A	9.6 W	1100	MCZ - CC BY-NC	Citation: Lynn Lucas and Lynn Copes provided access to these data, the collection of which was funded by NSF DDIG #0925793 and Wenner Gren Foundation #8102. The files were downloaded from www.MorphoSource.org, Duke University.
MCZ-37709	doi:10.17602/M2/M3043	2.84 GB	MCZ 37709 skull	Macaca fuscata	0.090751 mm	0.090751 mm	0.090751 mm	80 kv	110 A	8.8 W	1050	MCZ - CC BY-NC	Citation: Lynn Lucas and Lynn Copes provided access to these data, the collection of which was funded by NSF DDIG #0925793 and Wenner Gren Foundation #8102. The files were downloaded from www.MorphoSource.org, Duke University.
MCZ-37772	doi:10.17602/M2/M4607	1.96 GB	MCZ 37772 skull	Presbytis hosei	0.079988 mm	0.079988 mm	0.079988 mm	80 kv	120 A	9.6 W	1100	MCZ - CC BY-NC	Citation: Lynn Lucas and Lynn Copes provided access to these data, the collection of which was funded by NSF DDIG #0925793 and Wenner Gren Foundation #8102. The files were downloaded from www.MorphoSource.org, Duke University.
MCZ-37773	doi:10.17602/M2/M4606	1.99 GB	MCZ 37773 skull	Presbytis hosei	0.079988 mm	0.079988 mm	0.079988 mm	80 kv	120 A	9.6 W	1100	MCZ - CC BY-NC	Citation: Lynn Lucas and Lynn Copes provided access to these data, the collection of which was funded by NSF DDIG #0925793 and Wenner Gren Foundation #8102. The files were downloaded from www.MorphoSource.org, Duke University.
MCZ-37776	doi:10.17602/M2/M4547	1.85 GB	MCZ 37776 skull	Presbytis rubicunda	0.079988 mm	0.079988 mm	0.079988 mm	80 kv	120 A	9.6 W	1100	MCZ - CC BY-NC	Citation: Lynn Lucas and Lynn Copes provided access to these data, the collection of which was funded by NSF DDIG #0925793 and Wenner Gren Foundation #8102. The files were downloaded from www.MorphoSource.org, Duke University.
MCZ-37777	doi:10.17602/M2/M4546	1.66 GB	MCZ 37777 skull	Presbytis rubicunda	0.079988 mm	0.079988 mm	0.079988 mm	80 kv	120 A	9.6 W	1100	MCZ - CC BY-NC	Citation: Lynn Lucas and Lynn Copes provided access to these data, the collection of which was funded by NSF DDIG #0925793 and Wenner Gren Foundation #8102. The files were downloaded from www.MorphoSource.org, Duke University.
MCZ-37781	doi:10.17602/M2/M3037	3.64 GB	MCZ 37781 skull	Macaca fascicularis	0.061559 mm	0.061559 mm	0.061559 mm	80 kv	125 A	10 W	1050	MCZ - CC BY-NC	Citation: Lynn Lucas and Lynn Copes provided access to these data, the collection of which was funded by NSF DDIG #0925793 and Wenner Gren Foundation #8102. The files were downloaded from www.MorphoSource.org, Duke University.
MCZ-37795	doi:10.17602/M2/M2722	6.04 GB	MCZ 37795 skull	Nycticebus coucang	0.03046 mm	0.03046 mm	0.03046 mm	85 kv	125 A	10 W	1000	MCZ - CC BY-NC	Citation: Lynn Lucas and Lynn Copes provided access to these data, the collection of which was funded by NSF DDIG #0925793 and Wenner Gren Foundation #8102. The files were downloaded from www.MorphoSource.org, Duke University.
MCZ-37808	doi:10.17602/M2/M2706	3.35 GB	MCZ 37808 skull	Hapalemur griseus	0.038685 mm	0.038685 mm	0.038685 mm	80 kv	125 A	10 W	1000	MCZ - CC BY-NC	Citation: Lynn Lucas and Lynn Copes provided access to these data, the collection of which was funded by NSF DDIG #0925793 and Wenner Gren Foundation #8102. The files were downloaded from www.MorphoSource.org, Duke University.
MCZ-37823	doi:10.17602/M2/M5203	1.27 GB	MCZ 37823 skull	Callithrix jacchus	0.040724 mm	0.040724 mm	0.040724 mm	80 kv	125 A	10 W	1000	MCZ - CC BY-NC	Citation: Lynn Lucas and Lynn Copes provided access to these data, the collection of which was funded by NSF DDIG #0925793 and Wenner Gren Foundation #8102. The files were downloaded from www.MorphoSource.org, Duke University.
MCZ-37826	doi:10.17602/M2/M5202	1.2 GB	MCZ 37826 skull	Callithrix humeralifera	0.040724 mm	0.040724 mm	0.040724 mm	80 kv	125 A	10 W	1000	MCZ - CC BY-NC	Citation: Lynn Lucas and Lynn Copes provided access to these data, the collection of which was funded by NSF DDIG #0925793 and Wenner Gren Foundation #8102. The files were downloaded from www.MorphoSource.org, Duke University.
MCZ-37828	doi:10.17602/M2/M5171	1.62 GB	MCZ 37828 skull	Callicebus moloch	0.047097 mm	0.047097 mm	0.047097 mm	80 kv	120 A	9.6 W	1050	MCZ - CC BY-NC	Citation: Lynn Lucas and Lynn Copes provided access to these data, the collection of which was funded by NSF DDIG #0925793 and Wenner Gren Foundation #8102. The files were downloaded from www.MorphoSource.org, Duke University.
MCZ-37831	doi:10.17602/M2/M5217	2.37 GB	MCZ 37831 skull	Cebus apella	0.072065 mm	0.072065 mm	0.072065 mm	80 kv	115 A	9.2 W	1100	MCZ - CC BY-NC	Citation: Lynn Lucas and Lynn Copes provided access to these data, the collection of which was funded by NSF DDIG #0925793 and Wenner Gren Foundation #8102. The files were downloaded from www.MorphoSource.org, Duke University.
MCZ-37833	doi:10.17602/M2/M5218	5.34 GB	MCZ 37833 skull	Cebus apella	0.059654 mm	0.059654 mm	0.059654 mm	90 kv	120 A	10.8 W	1100	MCZ - CC BY-NC	Citation: Lynn Lucas and Lynn Copes provided access to these data, the collection of which was funded by NSF DDIG #0925793 and Wenner Gren Foundation #8102. The files were downloaded from www.MorphoSource.org, Duke University.
MCZ-38018	doi:10.17602/M2/M4399	3.54 GB	MCZ 38018 skull	Pan paniscus	0.094108 mm	0.094108 mm	0.094108 mm	80 kv	120 A	9.6 W	1500	MCZ - CC BY-NC	Citation: Lynn Lucas and Lynn Copes provided access to these data, the collection of which was funded by NSF DDIG #0925793 and Wenner Gren Foundation #8102. The files were downloaded from www.MorphoSource.org, Duke University.
MCZ-38019	doi:10.17602/M2/M4398	4.7 GB	MCZ 38019 skull	Pan paniscus	0.091894 mm	0.091894 mm	0.091894 mm	80 kv	120 A	9.6 W	1500	MCZ - CC BY-NC	Citation: Lynn Lucas and Lynn Copes provided access to these data, the collection of which was funded by NSF DDIG #0925793 and Wenner Gren Foundation #8102. The files were downloaded from www.MorphoSource.org, Duke University.
MCZ-38020	doi:10.17602/M2/M4397	4.33 GB	MCZ 38020 skull	Pan paniscus	0.099081 mm	0.099081 mm	0.099081 mm	80 kv	120 A	9.6 W	1500	MCZ - CC BY-NC	Citation: Lynn Lucas and Lynn Copes provided access to these data, the collection of which was funded by NSF DDIG #0925793 and Wenner Gren Foundation #8102. The files were downloaded from www.MorphoSource.org, Duke University.
MCZ-38083	doi:10.17602/M2/M2902	1.62 GB	MCZ 38083 skull	Colobus badius	0.079977 mm	0.079977 mm	0.079977 mm	80 kv	125 A	10 W	1100	MCZ - CC BY-NC	Citation: Lynn Lucas and Lynn Copes provided access to these data, the collection of which was funded by NSF DDIG #0925793 and Wenner Gren Foundation #8102. The files were downloaded from www.MorphoSource.org, Duke University.
MCZ-38326	doi:10.17602/M2/M2953	3.63 GB	MCZ 38326 cranium	Gorilla gorilla gorilla	0.121115 mm	0.121115 mm	0.121115 mm	85 kv	90 A	7.7 W	1500	MCZ - CC BY-NC	Citation: Lynn Lucas and Lynn Copes provided access to these data, the collection of which was funded by NSF DDIG #0925793 and Wenner Gren Foundation #8102. The files were downloaded from www.MorphoSource.org, Duke University.
MCZ-38326	doi:10.17602/M2/M2954	5.58 GB	MCZ 38326 mandible	Gorilla gorilla gorilla	0.08136 mm	0.08136 mm	0.08136 mm	85 kv	90 A	7.7 W	1000	MCZ - CC BY-NC	Citation: Lynn Lucas and Lynn Copes provided access to these data, the collection of which was funded by NSF DDIG #0925793 and Wenner Gren Foundation #8102. The files were downloaded from www.MorphoSource.org, Duke University.
MCZ-38875	doi:10.17602/M2/M2700	3.39 GB	MCZ 38875 skull	Galago senegalensis	0.023968 mm	0.023968 mm	0.023968 mm	80 kv	125 A	10 W	1000	MCZ - CC BY-NC	Citation: Lynn Lucas and Lynn Copes provided access to these data, the collection of which was funded by NSF DDIG #0925793 and Wenner Gren Foundation #8102. The files were downloaded from www.MorphoSource.org, Duke University.
MCZ-38912	doi:10.17602/M2/M2701	2.33 GB	MCZ 38912 skull	Galago senegalensis	0.027497 mm	0.027497 mm	0.027497 mm	80 kv	125 A	10 W	1000	MCZ - CC BY-NC	Citation: Lynn Lucas and Lynn Copes provided access to these data, the collection of which was funded by NSF DDIG #0925793 and Wenner Gren Foundation #8102. The files were downloaded from www.MorphoSource.org, Duke University.
MCZ-38915	doi:10.17602/M2/M2702	3.38 GB	MCZ 38915 skull	Galago senegalensis	0.025974 mm	0.025974 mm	0.025974 mm	80 kv	125 A	10 W	1000	MCZ - CC BY-NC	Citation: Lynn Lucas and Lynn Copes provided access to these data, the collection of which was funded by NSF DDIG #0925793 and Wenner Gren Foundation #8102. The files were downloaded from www.MorphoSource.org, Duke University.
MCZ-39073	doi:10.17602/M2/M5172	1.83 GB	MCZ 39073 skull	Callicebus moloch	0.047097 mm	0.047097 mm	0.047097 mm	80 kv	120 A	9.6 W	1050	MCZ - CC BY-NC	Citation: Lynn Lucas and Lynn Copes provided access to these data, the collection of which was funded by NSF DDIG #0925793 and Wenner Gren Foundation #8102. The files were downloaded from www.MorphoSource.org, Duke University.
MCZ-39375	doi:10.17602/M2/M2942	1.38 GB	MCZ 39375 skull	Cercopithecus mitis	0.079988 mm	0.079988 mm	0.079988 mm	80 kv	125 A	10 W	1100	MCZ - CC BY-NC	Citation: Lynn Lucas and Lynn Copes provided access to these data, the collection of which was funded by NSF DDIG #0925793 and Wenner Gren Foundation #8102. The files were downloaded from www.MorphoSource.org, Duke University.
MCZ-39388	doi:10.17602/M2/M2612	1.24 GB	MCZ 39388 skull	Cercocebus albigena	0.08971 mm	0.08971 mm	0.08971 mm	80 kv	125 A	10 W	1100	MCZ - CC BY-NC	Citation: Lynn Lucas and Lynn Copes provided access to these data, the collection of which was funded by NSF DDIG #0925793 and Wenner Gren Foundation #8102. The files were downloaded from www.MorphoSource.org, Duke University.
MCZ-39389	doi:10.17602/M2/M2933	1.6 GB	MCZ 39389 skull	Cercopithecus mitis	0.079988 mm	0.079988 mm	0.079988 mm	80 kv	125 A	10 W	1100	MCZ - CC BY-NC	Citation: Lynn Lucas and Lynn Copes provided access to these data, the collection of which was funded by NSF DDIG #0925793 and Wenner Gren Foundation #8102. The files were downloaded from www.MorphoSource.org, Duke University.
MCZ-39390	doi:10.17602/M2/M2934	1.5 GB	MCZ 39390 skull	Cercopithecus mitis	0.079988 mm	0.079988 mm	0.079988 mm	80 kv	125 A	10 W	1100	MCZ - CC BY-NC	Citation: Lynn Lucas and Lynn Copes provided access to these data, the collection of which was funded by NSF DDIG #0925793 and Wenner Gren Foundation #8102. The files were downloaded from www.MorphoSource.org, Duke University.
MCZ-39395	doi:10.17602/M2/M2608	2.12 GB	MCZ 39395 skull	Cercocebus albigena	0.08971 mm	0.08971 mm	0.08971 mm	80 kv	125 A	10 W	1100	MCZ - CC BY-NC	Citation: Lynn Lucas and Lynn Copes provided access to these data, the collection of which was funded by NSF DDIG #0925793 and Wenner Gren Foundation #8102. The files were downloaded from www.MorphoSource.org, Duke University.
MCZ-39396	doi:10.17602/M2/M2606	1.82 GB	MCZ 39396 skull	Cercocebus albigena	0.08971 mm	0.08971 mm	0.08971 mm	80 kv	125 A	10 W	1100	MCZ - CC BY-NC	Citation: Lynn Lucas and Lynn Copes provided access to these data, the collection of which was funded by NSF DDIG #0925793 and Wenner Gren Foundation #8102. The files were downloaded from www.MorphoSource.org, Duke University.
MCZ-39402	doi:10.17602/M2/M2604	2.51 GB	MCZ 39402 skull	Cercocebus albigena	0.08971 mm	0.08971 mm	0.08971 mm	80 kv	125 A	10 W	1100	MCZ - CC BY-NC	Citation: Lynn Lucas and Lynn Copes provided access to these data, the collection of which was funded by NSF DDIG #0925793 and Wenner Gren Foundation #8102. The files were downloaded from www.MorphoSource.org, Duke University.
MCZ-39562	doi:10.17602/M2/M5173	1.64 GB	MCZ 39562 skull	Callicebus moloch	0.047097 mm	0.047097 mm	0.047097 mm	80 kv	120 A	9.6 W	1050	MCZ - CC BY-NC	Citation: Lynn Lucas and Lynn Copes provided access to these data, the collection of which was funded by NSF DDIG #0925793 and Wenner Gren Foundation #8102. The files were downloaded from www.MorphoSource.org, Duke University.
MCZ-39563	doi:10.17602/M2/M5174	2.36 GB	MCZ 39563 skull	Callicebus moloch	0.047097 mm	0.047097 mm	0.047097 mm	80 kv	120 A	9.6 W	1050	MCZ - CC BY-NC	Citation: Lynn Lucas and Lynn Copes provided access to these data, the collection of which was funded by NSF DDIG #0925793 and Wenner Gren Foundation #8102. The files were downloaded from www.MorphoSource.org, Duke University.
MCZ-39571	doi:10.17602/M2/M2876	3.56 GB	MCZ 39571 skull	Aotus trivirgatus	0.040724 mm	0.040724 mm	0.040724 mm	75 kv	115 A	8.6 W	1000	MCZ - CC BY-NC	Citation: Lynn Lucas and Lynn Copes provided access to these data, the collection of which was funded by NSF DDIG #0925793 and Wenner Gren Foundation #8102. The files were downloaded from www.MorphoSource.org, Duke University.
MCZ-41090	doi:10.17602/M2/M5219	5.48 GB	MCZ 41090 skull	Cebus apella	0.048611 mm	0.048611 mm	0.048611 mm	90 kv	120 A	10.8 W	1100	MCZ - CC BY-NC	Citation: Lynn Lucas and Lynn Copes provided access to these data, the collection of which was funded by NSF DDIG #0925793 and Wenner Gren Foundation #8102. The files were downloaded from www.MorphoSource.org, Duke University.
MCZ-41167	doi:10.17602/M2/M3038	1.94 GB	MCZ 41167 skull	Macaca fascicularis	0.090906 mm	0.090906 mm	0.090906 mm	75 kv	120 A	9 W	1050	MCZ - CC BY-NC	Citation: Lynn Lucas and Lynn Copes provided access to these data, the collection of which was funded by NSF DDIG #0925793 and Wenner Gren Foundation #8102. The files were downloaded from www.MorphoSource.org, Duke University.
MCZ-41411	doi:10.17602/M2/M2960	419.14 MB	MCZ 41411 humerus and femur	Hylobates lar	0.125785 mm	0.125785 mm	0.125785 mm	90 kv	120 A	10.8 W	800	MCZ - CC BY-NC	Citation: Lynn Lucas and Lynn Copes provided access to these data, the collection of which was funded by NSF DDIG #0925793 and Wenner Gren Foundation #8102. The files were downloaded from www.MorphoSource.org, Duke University.
MCZ-41411	doi:10.17602/M2/M2959	2.48 GB	MCZ 41411 skull	Hylobates lar	0.066689 mm	0.066689 mm	0.066689 mm	90 kv	120 A	10.8 W	1050	MCZ - CC BY-NC	Citation: Lynn Lucas and Lynn Copes provided access to these data, the collection of which was funded by NSF DDIG #0925793 and Wenner Gren Foundation #8102. The files were downloaded from www.MorphoSource.org, Duke University.
MCZ-41412	doi:10.17602/M2/M2962	426.79 MB	MCZ 41412 humerus and femur	Hylobates lar	0.125785 mm	0.125785 mm	0.125785 mm	90 kv	120 A	10.8 W	800	MCZ - CC BY-NC	Citation: Lynn Lucas and Lynn Copes provided access to these data, the collection of which was funded by NSF DDIG #0925793 and Wenner Gren Foundation #8102. The files were downloaded from www.MorphoSource.org, Duke University.
MCZ-41412	doi:10.17602/M2/M2961	2.76 GB	MCZ 41412 skull	Hylobates lar	0.066689 mm	0.066689 mm	0.066689 mm	90 kv	120 A	10.8 W	1050	MCZ - CC BY-NC	Citation: Lynn Lucas and Lynn Copes provided access to these data, the collection of which was funded by NSF DDIG #0925793 and Wenner Gren Foundation #8102. The files were downloaded from www.MorphoSource.org, Duke University.
MCZ-41414	doi:10.17602/M2/M2964	800.24 MB	MCZ 41414 humerus and femur	Hylobates lar	0.125785 mm	0.125785 mm	0.125785 mm	90 kv	120 A	10.8 W	800	MCZ - CC BY-NC	Citation: Lynn Lucas and Lynn Copes provided access to these data, the collection of which was funded by NSF DDIG #0925793 and Wenner Gren Foundation #8102. The files were downloaded from www.MorphoSource.org, Duke University.
MCZ-41414	doi:10.17602/M2/M2963	2.4 GB	MCZ 41414 skull	Hylobates lar	0.066689 mm	0.066689 mm	0.066689 mm	90 kv	120 A	10.8 W	1050	MCZ - CC BY-NC	Citation: Lynn Lucas and Lynn Copes provided access to these data, the collection of which was funded by NSF DDIG #0925793 and Wenner Gren Foundation #8102. The files were downloaded from www.MorphoSource.org, Duke University.
MCZ-41416	doi:10.17602/M2/M2966	572.17 MB	MCZ 41416 humerus and femur	Hylobates lar	0.125785 mm	0.125785 mm	0.125785 mm	90 kv	120 A	10.8 W	800	MCZ - CC BY-NC	Citation: Lynn Lucas and Lynn Copes provided access to these data, the collection of which was funded by NSF DDIG #0925793 and Wenner Gren Foundation #8102. The files were downloaded from www.MorphoSource.org, Duke University.
MCZ-41416	doi:10.17602/M2/M2965	3.26 GB	MCZ 41416 skull	Hylobates lar	0.066619 mm	0.066619 mm	0.066619 mm	90 kv	120 A	10.8 W	1050	MCZ - CC BY-NC	Citation: Lynn Lucas and Lynn Copes provided access to these data, the collection of which was funded by NSF DDIG #0925793 and Wenner Gren Foundation #8102. The files were downloaded from www.MorphoSource.org, Duke University.
MCZ-41418	doi:10.17602/M2/M2968	486.16 MB	MCZ 41418 humerus and femur	Hylobates lar	0.125785 mm	0.125785 mm	0.125785 mm	90 kv	120 A	10.8 W	800	MCZ - CC BY-NC	Citation: Lynn Lucas and Lynn Copes provided access to these data, the collection of which was funded by NSF DDIG #0925793 and Wenner Gren Foundation #8102. The files were downloaded from www.MorphoSource.org, Duke University.
MCZ-41418	doi:10.17602/M2/M2967	2.37 GB	MCZ 41418 skull	Hylobates lar	0.066689 mm	0.066689 mm	0.066689 mm	90 kv	120 A	10.8 W	1050	MCZ - CC BY-NC	Citation: Lynn Lucas and Lynn Copes provided access to these data, the collection of which was funded by NSF DDIG #0925793 and Wenner Gren Foundation #8102. The files were downloaded from www.MorphoSource.org, Duke University.
MCZ-41421	doi:10.17602/M2/M2970	354.66 MB	MCZ 41421 humerus and femur	Hylobates lar	0.125785 mm	0.125785 mm	0.125785 mm	90 kv	120 A	10.8 W	800	MCZ - CC BY-NC	Citation: Lynn Lucas and Lynn Copes provided access to these data, the collection of which was funded by NSF DDIG #0925793 and Wenner Gren Foundation #8102. The files were downloaded from www.MorphoSource.org, Duke University.
MCZ-41421	doi:10.17602/M2/M2969	2.23 GB	MCZ 41421 skull	Hylobates lar	0.066689 mm	0.066689 mm	0.066689 mm	90 kv	120 A	10.8 W	1050	MCZ - CC BY-NC	Citation: Lynn Lucas and Lynn Copes provided access to these data, the collection of which was funded by NSF DDIG #0925793 and Wenner Gren Foundation #8102. The files were downloaded from www.MorphoSource.org, Duke University.
MCZ-41424	doi:10.17602/M2/M3008	528.68 MB	MCZ 41424 humerus and femur	Hylobates lar	0.125785 mm	0.125785 mm	0.125785 mm	90 kv	120 A	10.8 W	800	MCZ - CC BY-NC	Citation: Lynn Lucas and Lynn Copes provided access to these data, the collection of which was funded by NSF DDIG #0925793 and Wenner Gren Foundation #8102. The files were downloaded from www.MorphoSource.org, Duke University.
MCZ-41424	doi:10.17602/M2/M3007	3.6 GB	MCZ 41424 skull	Hylobates lar	0.066689 mm	0.066689 mm	0.066689 mm	90 kv	120 A	10.8 W	1050	MCZ - CC BY-NC	Citation: Lynn Lucas and Lynn Copes provided access to these data, the collection of which was funded by NSF DDIG #0925793 and Wenner Gren Foundation #8102. The files were downloaded from www.MorphoSource.org, Duke University.
MCZ-41436	doi:10.17602/M2/M3010	427.38 MB	MCZ 41436 humerus and femur	Hylobates lar	0.125785 mm	0.125785 mm	0.125785 mm	90 kv	125 A	11.3 W	800	MCZ - CC BY-NC	Citation: Lynn Lucas and Lynn Copes provided access to these data, the collection of which was funded by NSF DDIG #0925793 and Wenner Gren Foundation #8102. The files were downloaded from www.MorphoSource.org, Duke University.
MCZ-41440	doi:10.17602/M2/M4401	338.58 MB	MCZ 41440 humerus and femur	Hylobates lar	0.125785 mm	0.125785 mm	0.125785 mm	90 kv	125 A	11.3 W	800	MCZ - CC BY-NC	Citation: Lynn Lucas and Lynn Copes provided access to these data, the collection of which was funded by NSF DDIG #0925793 and Wenner Gren Foundation #8102. The files were downloaded from www.MorphoSource.org, Duke University.
MCZ-41440	doi:10.17602/M2/M4400	1.91 GB	MCZ 41440 skull	Hylobates lar	0.074155 mm	0.074155 mm	0.074155 mm	85 kv	125 A	10 W	1050	MCZ - CC BY-NC	Citation: Lynn Lucas and Lynn Copes provided access to these data, the collection of which was funded by NSF DDIG #0925793 and Wenner Gren Foundation #8102. The files were downloaded from www.MorphoSource.org, Duke University.
MCZ-41449	doi:10.17602/M2/M3009	436.21 MB	MCZ 41449 humerus and femur	Hylobates lar	0.125785 mm	0.125785 mm	0.125785 mm	90 kv	125 A	11.3 W	800	MCZ - CC BY-NC	Citation: Lynn Lucas and Lynn Copes provided access to these data, the collection of which was funded by NSF DDIG #0925793 and Wenner Gren Foundation #8102. The files were downloaded from www.MorphoSource.org, Duke University.
MCZ-41452	doi:10.17602/M2/M3011	1.6 GB	MCZ 41452 skull	Hylobates lar	0.07016 mm	0.07016 mm	0.07016 mm	85 kv	125 A	10 W	1050	MCZ - CC BY-NC	Citation: Lynn Lucas and Lynn Copes provided access to these data, the collection of which was funded by NSF DDIG #0925793 and Wenner Gren Foundation #8102. The files were downloaded from www.MorphoSource.org, Duke University.
MCZ-41454	doi:10.17602/M2/M3013	438.34 MB	MCZ 41454 humerus and femur	Hylobates lar	0.125785 mm	0.125785 mm	0.125785 mm	85 kv	125 A	10 W	800	MCZ - CC BY-NC	Citation: Lynn Lucas and Lynn Copes provided access to these data, the collection of which was funded by NSF DDIG #0925793 and Wenner Gren Foundation #8102. The files were downloaded from www.MorphoSource.org, Duke University.
MCZ-41454	doi:10.17602/M2/M3012	2.7 GB	MCZ 41454 skull	Hylobates lar	0.074155 mm	0.074155 mm	0.074155 mm	85 kv	125 A	10 W	1050	MCZ - CC BY-NC	Citation: Lynn Lucas and Lynn Copes provided access to these data, the collection of which was funded by NSF DDIG #0925793 and Wenner Gren Foundation #8102. The files were downloaded from www.MorphoSource.org, Duke University.
MCZ-41455	doi:10.17602/M2/M3015	407.11 MB	MCZ 41455 humerus and femur	Hylobates lar	0.125785 mm	0.125785 mm	0.125785 mm	90 kv	125 A	11.3 W	800	MCZ - CC BY-NC	Citation: Lynn Lucas and Lynn Copes provided access to these data, the collection of which was funded by NSF DDIG #0925793 and Wenner Gren Foundation #8102. The files were downloaded from www.MorphoSource.org, Duke University.
MCZ-41455	doi:10.17602/M2/M3014	2.74 GB	MCZ 41455 skull	Hylobates lar	0.074155 mm	0.074155 mm	0.074155 mm	85 kv	125 A	10 W	1050	MCZ - CC BY-NC	Citation: Lynn Lucas and Lynn Copes provided access to these data, the collection of which was funded by NSF DDIG #0925793 and Wenner Gren Foundation #8102. The files were downloaded from www.MorphoSource.org, Duke University.
MCZ-41457	doi:10.17602/M2/M3016	734.82 MB	MCZ 41457 humerus and femur	Hylobates lar	0.125785 mm	0.125785 mm	0.125785 mm	90 kv	125 A	11.3 W	800	MCZ - CC BY-NC	Citation: Lynn Lucas and Lynn Copes provided access to these data, the collection of which was funded by NSF DDIG #0925793 and Wenner Gren Foundation #8102. The files were downloaded from www.MorphoSource.org, Duke University.
MCZ-41458	doi:10.17602/M2/M3018	357.83 MB	MCZ 41458 humerus and femur	Hylobates lar	0.117471 mm	0.117471 mm	0.117471 mm	90 kv	125 A	11.3 W	800	MCZ - CC BY-NC	Citation: Lynn Lucas and Lynn Copes provided access to these data, the collection of which was funded by NSF DDIG #0925793 and Wenner Gren Foundation #8102. The files were downloaded from www.MorphoSource.org, Duke University.
MCZ-41458	doi:10.17602/M2/M3017	2.73 GB	MCZ 41458 skull	Hylobates lar	0.074155 mm	0.074155 mm	0.074155 mm	85 kv	125 A	10 W	1050	MCZ - CC BY-NC	Citation: Lynn Lucas and Lynn Copes provided access to these data, the collection of which was funded by NSF DDIG #0925793 and Wenner Gren Foundation #8102. The files were downloaded from www.MorphoSource.org, Duke University.
MCZ-41460	doi:10.17602/M2/M3020	687.51 MB	MCZ 41460 humerus and femur	Hylobates lar	0.125785 mm	0.125785 mm	0.125785 mm	90 kv	125 A	11.3 W	800	MCZ - CC BY-NC	Citation: Lynn Lucas and Lynn Copes provided access to these data, the collection of which was funded by NSF DDIG #0925793 and Wenner Gren Foundation #8102. The files were downloaded from www.MorphoSource.org, Duke University.
MCZ-41460	doi:10.17602/M2/M3019	3.2 GB	MCZ 41460 skull	Hylobates lar	0.074155 mm	0.074155 mm	0.074155 mm	85 kv	125 A	10 W	1050	MCZ - CC BY-NC	Citation: Lynn Lucas and Lynn Copes provided access to these data, the collection of which was funded by NSF DDIG #0925793 and Wenner Gren Foundation #8102. The files were downloaded from www.MorphoSource.org, Duke University.
MCZ-41463	doi:10.17602/M2/M3022	371.74 MB	MCZ 41463 humerus and femur	Hylobates lar	0.125785 mm	0.125785 mm	0.125785 mm	90 kv	125 A	11.3 W	800	MCZ - CC BY-NC	Citation: Lynn Lucas and Lynn Copes provided access to these data, the collection of which was funded by NSF DDIG #0925793 and Wenner Gren Foundation #8102. The files were downloaded from www.MorphoSource.org, Duke University.
MCZ-41463	doi:10.17602/M2/M3021	2.71 GB	MCZ 41463 skull	Hylobates lar	0.074155 mm	0.074155 mm	0.074155 mm	85 kv	125 A	10 W	1050	MCZ - CC BY-NC	Citation: Lynn Lucas and Lynn Copes provided access to these data, the collection of which was funded by NSF DDIG #0925793 and Wenner Gren Foundation #8102. The files were downloaded from www.MorphoSource.org, Duke University.
MCZ-41469	doi:10.17602/M2/M3024	529.07 MB	MCZ 41469 humerus and femur	Hylobates lar	0.125785 mm	0.125785 mm	0.125785 mm	90 kv	125 A	11.3 W	800	MCZ - CC BY-NC	Citation: Lynn Lucas and Lynn Copes provided access to these data, the collection of which was funded by NSF DDIG #0925793 and Wenner Gren Foundation #8102. The files were downloaded from www.MorphoSource.org, Duke University.
MCZ-41469	doi:10.17602/M2/M3023	2.92 GB	MCZ 41469 skull	Hylobates lar	0.074155 mm	0.074155 mm	0.074155 mm	85 kv	125 A	10 W	1050	MCZ - CC BY-NC	Citation: Lynn Lucas and Lynn Copes provided access to these data, the collection of which was funded by NSF DDIG #0925793 and Wenner Gren Foundation #8102. The files were downloaded from www.MorphoSource.org, Duke University.
MCZ-41493	doi:10.17602/M2/M3026	430.28 MB	MCZ 41493 humerus and femur	Hylobates lar	0.125785 mm	0.125785 mm	0.125785 mm	85 kv	125 A	10 W	800	MCZ - CC BY-NC	Citation: Lynn Lucas and Lynn Copes provided access to these data, the collection of which was funded by NSF DDIG #0925793 and Wenner Gren Foundation #8102. The files were downloaded from www.MorphoSource.org, Duke University.
MCZ-41493	doi:10.17602/M2/M3025	2.8 GB	MCZ 41493 skull	Hylobates lar	0.074155 mm	0.074155 mm	0.074155 mm	85 kv	125 A	10 W	1050	MCZ - CC BY-NC	Citation: Lynn Lucas and Lynn Copes provided access to these data, the collection of which was funded by NSF DDIG #0925793 and Wenner Gren Foundation #8102. The files were downloaded from www.MorphoSource.org, Duke University.
MCZ-41554	doi:10.17602/M2/M5060	2.26 GB	MCZ 41554 skull	Nasalis larvatus	0.070602 mm	0.070602 mm	0.070602 mm	85 kv	125 A	10 W	1000	MCZ - CC BY-NC	Citation: Lynn Lucas and Lynn Copes provided access to these data, the collection of which was funded by NSF DDIG #0925793 and Wenner Gren Foundation #8102. The files were downloaded from www.MorphoSource.org, Duke University.
MCZ-41555	doi:10.17602/M2/M5059	2.51 GB	MCZ 41555 skull	Nasalis larvatus	0.070602 mm	0.070602 mm	0.070602 mm	85 kv	125 A	10 W	1000	MCZ - CC BY-NC	Citation: Lynn Lucas and Lynn Copes provided access to these data, the collection of which was funded by NSF DDIG #0925793 and Wenner Gren Foundation #8102. The files were downloaded from www.MorphoSource.org, Duke University.
MCZ-41556	doi:10.17602/M2/M5058	2.65 GB	MCZ 41556 skull	Nasalis larvatus	0.070602 mm	0.070602 mm	0.070602 mm	85 kv	125 A	10 W	1000	MCZ - CC BY-NC	Citation: Lynn Lucas and Lynn Copes provided access to these data, the collection of which was funded by NSF DDIG #0925793 and Wenner Gren Foundation #8102. The files were downloaded from www.MorphoSource.org, Duke University.
MCZ-41559	doi:10.17602/M2/M5057	2.25 GB	MCZ 41559 skull	Nasalis larvatus	0.070602 mm	0.070602 mm	0.070602 mm	85 kv	125 A	10 W	1000	MCZ - CC BY-NC	Citation: Lynn Lucas and Lynn Copes provided access to these data, the collection of which was funded by NSF DDIG #0925793 and Wenner Gren Foundation #8102. The files were downloaded from www.MorphoSource.org, Duke University.
MCZ-41560	doi:10.17602/M2/M5056	2.49 GB	MCZ 41560 skull	Nasalis larvatus	0.070962 mm	0.070962 mm	0.070962 mm	80 kv	110 A	8.8 W	1100	MCZ - CC BY-NC	Citation: Lynn Lucas and Lynn Copes provided access to these data, the collection of which was funded by NSF DDIG #0925793 and Wenner Gren Foundation #8102. The files were downloaded from www.MorphoSource.org, Duke University.
MCZ-41562	doi:10.17602/M2/M5055	2.52 GB	MCZ 41562 skull	Nasalis larvatus	0.070602 mm	0.070602 mm	0.070602 mm	85 kv	125 A	10 W	1000	MCZ - CC BY-NC	Citation: Lynn Lucas and Lynn Copes provided access to these data, the collection of which was funded by NSF DDIG #0925793 and Wenner Gren Foundation #8102. The files were downloaded from www.MorphoSource.org, Duke University.
MCZ-41567	doi:10.17602/M2/M4496	1.31 GB	MCZ 41567 skull	Saguinus sp.	0.040724 mm	0.040724 mm	0.040724 mm	80 kv	125 A	10 W	1000	MCZ - CC BY-NC	Citation: Lynn Lucas and Lynn Copes provided access to these data, the collection of which was funded by NSF DDIG #0925793 and Wenner Gren Foundation #8102. The files were downloaded from www.MorphoSource.org, Duke University.
MCZ-41568	doi:10.17602/M2/M4495	1.53 GB	MCZ 41568 skull	Saguinus sp.	0.040724 mm	0.040724 mm	0.040724 mm	80 kv	125 A	10 W	1000	MCZ - CC BY-NC	Citation: Lynn Lucas and Lynn Copes provided access to these data, the collection of which was funded by NSF DDIG #0925793 and Wenner Gren Foundation #8102. The files were downloaded from www.MorphoSource.org, Duke University.
MCZ-42620	doi:10.17602/M2/M4715	5.2 GB	MCZ 42620 skull	Perodicticus potto	0.032828 mm	0.032828 mm	0.032828 mm	80 kv	125 A	10 W	1000	MCZ - CC BY-NC	Citation: Lynn Lucas and Lynn Copes provided access to these data, the collection of which was funded by NSF DDIG #0925793 and Wenner Gren Foundation #8102. The files were downloaded from www.MorphoSource.org, Duke University.
MCZ-42621	doi:10.17602/M2/M4714	4.03 GB	MCZ 42621 skull	Perodicticus potto	0.039517 mm	0.039517 mm	0.039517 mm	80 kv	125 A	10 W	1000	MCZ - CC BY-NC	Citation: Lynn Lucas and Lynn Copes provided access to these data, the collection of which was funded by NSF DDIG #0925793 and Wenner Gren Foundation #8102. The files were downloaded from www.MorphoSource.org, Duke University.
MCZ-42622	doi:10.17602/M2/M4712	4.79 GB	MCZ 42622 skull	Perodicticus potto	0.037048 mm	0.037048 mm	0.037048 mm	80 kv	125 A	10 W	1000	MCZ - CC BY-NC	Citation: Lynn Lucas and Lynn Copes provided access to these data, the collection of which was funded by NSF DDIG #0925793 and Wenner Gren Foundation #8102. The files were downloaded from www.MorphoSource.org, Duke University.
MCZ-42623	doi:10.17602/M2/M4713	3.79 GB	MCZ 42623 skull	Perodicticus potto	0.039517 mm	0.039517 mm	0.039517 mm	80 kv	125 A	10 W	1000	MCZ - CC BY-NC	Citation: Lynn Lucas and Lynn Copes provided access to these data, the collection of which was funded by NSF DDIG #0925793 and Wenner Gren Foundation #8102. The files were downloaded from www.MorphoSource.org, Duke University.
MCZ-43484	doi:10.17602/M2/M4445	1.72 GB	MCZ 43484 skull	Saimiri sciureus	0.047018 mm	0.047018 mm	0.047018 mm	80 kv	120 A	9.6 W	1050	MCZ - CC BY-NC	Citation: Lynn Lucas and Lynn Copes provided access to these data, the collection of which was funded by NSF DDIG #0925793 and Wenner Gren Foundation #8102. The files were downloaded from www.MorphoSource.org, Duke University.
MCZ-44132	doi:10.17602/M2/M2703	2.48 GB	MCZ 44132 skull	Galago senegalensis	0.027497 mm	0.027497 mm	0.027497 mm	80 kv	125 A	10 W	1000	MCZ - CC BY-NC	Citation: Lynn Lucas and Lynn Copes provided access to these data, the collection of which was funded by NSF DDIG #0925793 and Wenner Gren Foundation #8102. The files were downloaded from www.MorphoSource.org, Duke University.
MCZ-44134	doi:10.17602/M2/M2704	3.15 GB	MCZ 44134 skull	Galago senegalensis	0.027497 mm	0.027497 mm	0.027497 mm	80 kv	125 A	10 W	1000	MCZ - CC BY-NC	Citation: Lynn Lucas and Lynn Copes provided access to these data, the collection of which was funded by NSF DDIG #0925793 and Wenner Gren Foundation #8102. The files were downloaded from www.MorphoSource.org, Duke University.
MCZ-44264	doi:10.17602/M2/M2935	1.75 GB	MCZ 44264 skull	Cercopithecus mitis	0.079988 mm	0.079988 mm	0.079988 mm	80 kv	125 A	10 W	1100	MCZ - CC BY-NC	Citation: Lynn Lucas and Lynn Copes provided access to these data, the collection of which was funded by NSF DDIG #0925793 and Wenner Gren Foundation #8102. The files were downloaded from www.MorphoSource.org, Duke University.
MCZ-44268	doi:10.17602/M2/M2936	2.07 GB	MCZ 44268 skull	Cercopithecus mitis	0.079988 mm	0.079988 mm	0.079988 mm	80 kv	125 A	10 W	1100	MCZ - CC BY-NC	Citation: Lynn Lucas and Lynn Copes provided access to these data, the collection of which was funded by NSF DDIG #0925793 and Wenner Gren Foundation #8102. The files were downloaded from www.MorphoSource.org, Duke University.
MCZ-44274	doi:10.17602/M2/M2937	1.55 GB	MCZ 44274 skull	Cercopithecus mitis	0.079988 mm	0.079988 mm	0.079988 mm	80 kv	125 A	10 W	1100	MCZ - CC BY-NC	Citation: Lynn Lucas and Lynn Copes provided access to these data, the collection of which was funded by NSF DDIG #0925793 and Wenner Gren Foundation #8102. The files were downloaded from www.MorphoSource.org, Duke University.
MCZ-44839	doi:10.17602/M2/M2844	4.19 GB	MCZ 44839 skull	Microcebus murinus	0.018255 mm	0.018255 mm	0.018255 mm	80 kv	120 A	9.6 W	800	MCZ - CC BY-NC	Citation: Lynn Lucas and Lynn Copes provided access to these data, the collection of which was funded by NSF DDIG #0925793 and Wenner Gren Foundation #8102. The files were downloaded from www.MorphoSource.org, Duke University.
MCZ-44840	doi:10.17602/M2/M2845	5.8 GB	MCZ 44840 skull	Microcebus murinus	0.029811 mm	0.029811 mm	0.029811 mm	75 kv	115 A	8.1 W	1000	MCZ - CC BY-NC	Citation: Lynn Lucas and Lynn Copes provided access to these data, the collection of which was funded by NSF DDIG #0925793 and Wenner Gren Foundation #8102. The files were downloaded from www.MorphoSource.org, Duke University.
MCZ-44842	doi:10.17602/M2/M2846	5.63 GB	MCZ 44842 skull	Microcebus murinus	0.016473 mm	0.016473 mm	0.016473 mm	75 kv	115 A	8.1 W	1000	MCZ - CC BY-NC	Citation: Lynn Lucas and Lynn Copes provided access to these data, the collection of which was funded by NSF DDIG #0925793 and Wenner Gren Foundation #8102. The files were downloaded from www.MorphoSource.org, Duke University.
MCZ-44843	doi:10.17602/M2/M2847	3.68 GB	MCZ 44843 skull	Microcebus murinus	0.018205 mm	0.018205 mm	0.018205 mm	75 kv	115 A	8.1 W	1000	MCZ - CC BY-NC	Citation: Lynn Lucas and Lynn Copes provided access to these data, the collection of which was funded by NSF DDIG #0925793 and Wenner Gren Foundation #8102. The files were downloaded from www.MorphoSource.org, Duke University.
MCZ-44847	doi:10.17602/M2/M2848	3.99 GB	MCZ 44847 skull	Microcebus murinus	0.017617 mm	0.017617 mm	0.017617 mm	80 kv	120 A	9.6 W	800	MCZ - CC BY-NC	Citation: Lynn Lucas and Lynn Copes provided access to these data, the collection of which was funded by NSF DDIG #0925793 and Wenner Gren Foundation #8102. The files were downloaded from www.MorphoSource.org, Duke University.
MCZ-44854	doi:10.17602/M2/M2726	4.32 GB	MCZ 44854 skull	Propithecus verreauxi	0.048998 mm	0.048998 mm	0.048998 mm	80 kv	125 A	10 W	1000	MCZ - CC BY-NC	Citation: Lynn Lucas and Lynn Copes provided access to these data, the collection of which was funded by NSF DDIG #0925793 and Wenner Gren Foundation #8102. The files were downloaded from www.MorphoSource.org, Duke University.
MCZ-44855	doi:10.17602/M2/M4502	4.77 GB	MCZ 44855 skull	Propithecus diadema	0.048998 mm	0.048998 mm	0.048998 mm	80 kv	125 A	10 W	1000	MCZ - CC BY-NC	Citation: Lynn Lucas and Lynn Copes provided access to these data, the collection of which was funded by NSF DDIG #0925793 and Wenner Gren Foundation #8102. The files were downloaded from www.MorphoSource.org, Duke University.
MCZ-44856	doi:10.17602/M2/M2727	3.31 GB	MCZ 44856 skull	Propithecus verreauxi	0.048998 mm	0.048998 mm	0.048998 mm	80 kv	125 A	10 W	1000	MCZ - CC BY-NC	Citation: Lynn Lucas and Lynn Copes provided access to these data, the collection of which was funded by NSF DDIG #0925793 and Wenner Gren Foundation #8102. The files were downloaded from www.MorphoSource.org, Duke University.
MCZ-44877	doi:10.17602/M2/M2731	4 GB	MCZ 44877 skull	Avahi laniger	0.028312 mm	0.028312 mm	0.028312 mm	80 kv	125 A	10 W	1000	MCZ - CC BY-NC	Citation: Lynn Lucas and Lynn Copes provided access to these data, the collection of which was funded by NSF DDIG #0925793 and Wenner Gren Foundation #8102. The files were downloaded from www.MorphoSource.org, Duke University.
MCZ-44878	doi:10.17602/M2/M2714	5.81 GB	MCZ 44878 skull	Avahi laniger	0.030704 mm	0.030704 mm	0.030704 mm	80 kv	125 A	10 W	1000	MCZ - CC BY-NC	Citation: Lynn Lucas and Lynn Copes provided access to these data, the collection of which was funded by NSF DDIG #0925793 and Wenner Gren Foundation #8102. The files were downloaded from www.MorphoSource.org, Duke University.
MCZ-44879	doi:10.17602/M2/M2715	3.76 GB	MCZ 44879 skull	Avahi laniger	0.030705 mm	0.030705 mm	0.030705 mm	80 kv	125 A	10 W	1000	MCZ - CC BY-NC	Citation: Lynn Lucas and Lynn Copes provided access to these data, the collection of which was funded by NSF DDIG #0925793 and Wenner Gren Foundation #8102. The files were downloaded from www.MorphoSource.org, Duke University.
MCZ-44886	doi:10.17602/M2/M2663	4.42 GB	MCZ 44886 skull	Eulemur fulvus rufus	0.047639 mm	0.047639 mm	0.047639 mm	80 kv	125 A	10 W	1000	MCZ - CC BY-NC	Citation: Lynn Lucas and Lynn Copes provided access to these data, the collection of which was funded by NSF DDIG #0925793 and Wenner Gren Foundation #8102. The files were downloaded from www.MorphoSource.org, Duke University.
MCZ-44887	doi:10.17602/M2/M2670	4.52 GB	MCZ 44887 skull	Eulemur fulvus collaris	0.047648 mm	0.047648 mm	0.047648 mm	80 kv	125 A	10 W	1000	MCZ - CC BY-NC	Citation: Lynn Lucas and Lynn Copes provided access to these data, the collection of which was funded by NSF DDIG #0925793 and Wenner Gren Foundation #8102. The files were downloaded from www.MorphoSource.org, Duke University.
MCZ-44889	doi:10.17602/M2/M2675	4.02 GB	MCZ 44889 skull	Eulemur fulvus collaris	0.047648 mm	0.047648 mm	0.047648 mm	80 kv	125 A	10 W	1000	MCZ - CC BY-NC	Citation: Lynn Lucas and Lynn Copes provided access to these data, the collection of which was funded by NSF DDIG #0925793 and Wenner Gren Foundation #8102. The files were downloaded from www.MorphoSource.org, Duke University.
MCZ-44892	doi:10.17602/M2/M2679	4.1 GB	MCZ 44892 skull	Eulemur fulvus collaris	0.044297 mm	0.044297 mm	0.044297 mm	80 kv	125 A	10 W	1000	MCZ - CC BY-NC	Citation: Lynn Lucas and Lynn Copes provided access to these data, the collection of which was funded by NSF DDIG #0925793 and Wenner Gren Foundation #8102. The files were downloaded from www.MorphoSource.org, Duke University.
MCZ-44893	doi:10.17602/M2/M2682	3.96 GB	MCZ 44893 skull	Eulemur fulvus collaris	0.045991 mm	0.045991 mm	0.045991 mm	80 kv	125 A	10 W	1000	MCZ - CC BY-NC	Citation: Lynn Lucas and Lynn Copes provided access to these data, the collection of which was funded by NSF DDIG #0925793 and Wenner Gren Foundation #8102. The files were downloaded from www.MorphoSource.org, Duke University.
MCZ-44895	doi:10.17602/M2/M2683	4.71 GB	MCZ 44895 skull	Eulemur fulvus collaris	0.047648 mm	0.047648 mm	0.047648 mm	80 kv	125 A	10 W	1000	MCZ - CC BY-NC	Citation: Lynn Lucas and Lynn Copes provided access to these data, the collection of which was funded by NSF DDIG #0925793 and Wenner Gren Foundation #8102. The files were downloaded from www.MorphoSource.org, Duke University.
MCZ-44896	doi:10.17602/M2/M2684	2.95 GB	MCZ 44896 skull	Eulemur fulvus collaris	0.047648 mm	0.047648 mm	0.047648 mm	80 kv	125 A	10 W	1000	MCZ - CC BY-NC	Citation: Lynn Lucas and Lynn Copes provided access to these data, the collection of which was funded by NSF DDIG #0925793 and Wenner Gren Foundation #8102. The files were downloaded from www.MorphoSource.org, Duke University.
MCZ-44903	doi:10.17602/M2/M2841	2.36 GB	MCZ 44903 skull	Lemur catta	0.044601 mm	0.044601 mm	0.044601 mm	80 kv	125 A	10 W	1000	MCZ - CC BY-NC	Citation: Lynn Lucas and Lynn Copes provided access to these data, the collection of which was funded by NSF DDIG #0925793 and Wenner Gren Foundation #8102. The files were downloaded from www.MorphoSource.org, Duke University.
MCZ-44904	doi:10.17602/M2/M2941	2.62 GB	MCZ 44904 skull	Lemur catta	0.044601 mm	0.044601 mm	0.044601 mm	80 kv	125 A	10 W	1000	MCZ - CC BY-NC	Citation: Lynn Lucas and Lynn Copes provided access to these data, the collection of which was funded by NSF DDIG #0925793 and Wenner Gren Foundation #8102. The files were downloaded from www.MorphoSource.org, Duke University.
MCZ-44905	doi:10.17602/M2/M2729	4.14 GB	MCZ 44905 skull	Varecia variegata variegata	0.054763 mm	0.054763 mm	0.054763 mm	80 kv	125 A	10 W	1000	MCZ - CC BY-NC	Citation: Lynn Lucas and Lynn Copes provided access to these data, the collection of which was funded by NSF DDIG #0925793 and Wenner Gren Foundation #8102. The files were downloaded from www.MorphoSource.org, Duke University.
MCZ-44906	doi:10.17602/M2/M2734	4 GB	MCZ 44906 skull	Varecia variegata variegata	0.054763 mm	0.054763 mm	0.054763 mm	80 kv	125 A	10 W	1000	MCZ - CC BY-NC	Citation: Lynn Lucas and Lynn Copes provided access to these data, the collection of which was funded by NSF DDIG #0925793 and Wenner Gren Foundation #8102. The files were downloaded from www.MorphoSource.org, Duke University.
MCZ-44907	doi:10.17602/M2/M2735	2.5 GB	MCZ 44907 skull	Varecia variegata	0.054763 mm	0.054763 mm	0.054763 mm	80 kv	125 A	10 W	1000	MCZ - CC BY-NC	Citation: Lynn Lucas and Lynn Copes provided access to these data, the collection of which was funded by NSF DDIG #0925793 and Wenner Gren Foundation #8102. The files were downloaded from www.MorphoSource.org, Duke University.
MCZ-44909	doi:10.17602/M2/M2736	2.54 GB	MCZ 44909 skull	Varecia variegata	0.054763 mm	0.054763 mm	0.054763 mm	80 kv	125 A	10 W	1000	MCZ - CC BY-NC	Citation: Lynn Lucas and Lynn Copes provided access to these data, the collection of which was funded by NSF DDIG #0925793 and Wenner Gren Foundation #8102. The files were downloaded from www.MorphoSource.org, Duke University.
MCZ-44911	doi:10.17602/M2/M2707	3.95 GB	MCZ 44911 skull	Hapalemur griseus	0.038685 mm	0.038685 mm	0.038685 mm	80 kv	125 A	10 W	1000	MCZ - CC BY-NC	Citation: Lynn Lucas and Lynn Copes provided access to these data, the collection of which was funded by NSF DDIG #0925793 and Wenner Gren Foundation #8102. The files were downloaded from www.MorphoSource.org, Duke University.
MCZ-44913	doi:10.17602/M2/M2708	2.9 GB	MCZ 44913 skull	Hapalemur griseus	0.038685 mm	0.038685 mm	0.038685 mm	80 kv	125 A	10 W	1000	MCZ - CC BY-NC	Citation: Lynn Lucas and Lynn Copes provided access to these data, the collection of which was funded by NSF DDIG #0925793 and Wenner Gren Foundation #8102. The files were downloaded from www.MorphoSource.org, Duke University.
MCZ-44918	doi:10.17602/M2/M2709	3.98 GB	MCZ 44918 skull	Hapalemur griseus	0.038685 mm	0.038685 mm	0.038685 mm	80 kv	125 A	10 W	1000	MCZ - CC BY-NC	Citation: Lynn Lucas and Lynn Copes provided access to these data, the collection of which was funded by NSF DDIG #0925793 and Wenner Gren Foundation #8102. The files were downloaded from www.MorphoSource.org, Duke University.
MCZ-44921	doi:10.17602/M2/M2710	3.36 GB	MCZ 44921 skull	Hapalemur griseus	0.038685 mm	0.038685 mm	0.038685 mm	80 kv	125 A	10 W	1000	MCZ - CC BY-NC	Citation: Lynn Lucas and Lynn Copes provided access to these data, the collection of which was funded by NSF DDIG #0925793 and Wenner Gren Foundation #8102. The files were downloaded from www.MorphoSource.org, Duke University.
MCZ-44922	doi:10.17602/M2/M2711	4.06 GB	MCZ 44922 skull	Hapalemur griseus	0.03872 mm	0.03872 mm	0.03872 mm	80 kv	125 A	10 W	1000	MCZ - CC BY-NC	Citation: Lynn Lucas and Lynn Copes provided access to these data, the collection of which was funded by NSF DDIG #0925793 and Wenner Gren Foundation #8102. The files were downloaded from www.MorphoSource.org, Duke University.
MCZ-44923	doi:10.17602/M2/M2712	4.16 GB	MCZ 44923 skull	Hapalemur griseus	0.038685 mm	0.038685 mm	0.038685 mm	80 kv	125 A	10 W	1000	MCZ - CC BY-NC	Citation: Lynn Lucas and Lynn Copes provided access to these data, the collection of which was funded by NSF DDIG #0925793 and Wenner Gren Foundation #8102. The files were downloaded from www.MorphoSource.org, Duke University.
MCZ-45125	doi:10.17602/M2/M2849	3.31 GB	MCZ 45125 skull	Microcebus murinus	0.01853 mm	0.01853 mm	0.01853 mm	80 kv	120 A	9.6 W	800	MCZ - CC BY-NC	Citation: Lynn Lucas and Lynn Copes provided access to these data, the collection of which was funded by NSF DDIG #0925793 and Wenner Gren Foundation #8102. The files were downloaded from www.MorphoSource.org, Duke University.
MCZ-46143	doi:10.17602/M2/M2830	4.38 GB	MCZ 46143 skull	Euoticus elegantulus	0.026906 mm	0.026906 mm	0.026906 mm	80 kv	125 A	10 W	1000	MCZ - CC BY-NC	Citation: Lynn Lucas and Lynn Copes provided access to these data, the collection of which was funded by NSF DDIG #0925793 and Wenner Gren Foundation #8102. The files were downloaded from www.MorphoSource.org, Duke University.
MCZ-46325	doi:10.17602/M2/M2955	3.76 GB	MCZ 46325 cranium	Gorilla gorilla gorilla	0.125974 mm	0.125974 mm	0.125974 mm	85 kv	90 A	7.7 W	1500	MCZ - CC BY-NC	Citation: Lynn Lucas and Lynn Copes provided access to these data, the collection of which was funded by NSF DDIG #0925793 and Wenner Gren Foundation #8102. The files were downloaded from www.MorphoSource.org, Duke University.
MCZ-46325	doi:10.17602/M2/M2956	6.57 GB	MCZ 46325 mandible	Gorilla gorilla gorilla	0.08136 mm	0.08136 mm	0.08136 mm	85 kv	90 A	77 W	1000	MCZ - CC BY-NC	Citation: Lynn Lucas and Lynn Copes provided access to these data, the collection of which was funded by NSF DDIG #0925793 and Wenner Gren Foundation #8102. The files were downloaded from www.MorphoSource.org, Duke University.
MCZ-46368	doi:10.17602/M2/M2913	3.59 GB	MCZ 46368 skull	Colobus polykomos	0.07602 mm	0.07602 mm	0.07602 mm	70 kv	110 A	7.7 W	1050	MCZ - CC BY-NC	Citation: Lynn Lucas and Lynn Copes provided access to these data, the collection of which was funded by NSF DDIG #0925793 and Wenner Gren Foundation #8102. The files were downloaded from www.MorphoSource.org, Duke University.
MCZ-46414	doi:10.17602/M2/M4386	3.8 GB	MCZ 46414 cranium	Pan troglodytes	0.08222 mm	0.08222 mm	0.08222 mm	80 kv	120 A	9.6 W	1300	MCZ - CC BY-NC	Citation: Lynn Lucas and Lynn Copes provided access to these data, the collection of which was funded by NSF DDIG #0925793 and Wenner Gren Foundation #8102. The files were downloaded from www.MorphoSource.org, Duke University.
MCZ-46415	doi:10.17602/M2/M4385	3.38 GB	MCZ 46415 cranium	Pan troglodytes	0.086138 mm	0.086138 mm	0.086138 mm	80 kv	120 A	9.6 W	1300	MCZ - CC BY-NC	Citation: Lynn Lucas and Lynn Copes provided access to these data, the collection of which was funded by NSF DDIG #0925793 and Wenner Gren Foundation #8102. The files were downloaded from www.MorphoSource.org, Duke University.
MCZ-46416	doi:10.17602/M2/M4384	3.68 GB	MCZ 46416 skull	Pan troglodytes	0.099918 mm	0.099918 mm	0.099918 mm	80 kv	120 A	9.6 W	1500	MCZ - CC BY-NC	Citation: Lynn Lucas and Lynn Copes provided access to these data, the collection of which was funded by NSF DDIG #0925793 and Wenner Gren Foundation #8102. The files were downloaded from www.MorphoSource.org, Duke University.
MCZ-47007	doi:10.17602/M2/M2914	2.75 GB	MCZ 47007 skull	Colobus polykomos	0.07602 mm	0.07602 mm	0.07602 mm	70 kv	110 A	7.7 W	1050	MCZ - CC BY-NC	Citation: Lynn Lucas and Lynn Copes provided access to these data, the collection of which was funded by NSF DDIG #0925793 and Wenner Gren Foundation #8102. The files were downloaded from www.MorphoSource.org, Duke University.
MCZ-47015	doi:10.17602/M2/M2923	4.39 GB	MCZ 47015 skull	Erythrocebus patas pyrrhonotus	0.082531 mm	0.082531 mm	0.082531 mm	80 kv	125 A	10 W	1100	MCZ - CC BY-NC	Citation: Lynn Lucas and Lynn Copes provided access to these data, the collection of which was funded by NSF DDIG #0925793 and Wenner Gren Foundation #8102. The files were downloaded from www.MorphoSource.org, Duke University.
MCZ-47016	doi:10.17602/M2/M2924	4.21 GB	MCZ 47016 skull	Erythrocebus patas pyrrhonotus	0.082531 mm	0.082531 mm	0.082531 mm	80 kv	125 A	10 W	1100	MCZ - CC BY-NC	Citation: Lynn Lucas and Lynn Copes provided access to these data, the collection of which was funded by NSF DDIG #0925793 and Wenner Gren Foundation #8102. The files were downloaded from www.MorphoSource.org, Duke University.
MCZ-47017	doi:10.17602/M2/M2925	8.5 GB	MCZ 47017 skull	Erythrocebus patas pyrrhonotus	0.061559 mm	0.061559 mm	0.061559 mm	80 kv	125 A	10 W	1100	MCZ - CC BY-NC	Citation: Lynn Lucas and Lynn Copes provided access to these data, the collection of which was funded by NSF DDIG #0925793 and Wenner Gren Foundation #8102. The files were downloaded from www.MorphoSource.org, Duke University.
MCZ-47018	doi:10.17602/M2/M2926	2.48 GB	MCZ 47018 skull	Erythrocebus patas	0.082531 mm	0.082531 mm	0.082531 mm	80 kv	125 A	10 W	1100	MCZ - CC BY-NC	Citation: Lynn Lucas and Lynn Copes provided access to these data, the collection of which was funded by NSF DDIG #0925793 and Wenner Gren Foundation #8102. The files were downloaded from www.MorphoSource.org, Duke University.
MCZ-49006	doi:10.17602/M2/M2957	2.91 GB	MCZ 49006 cranium	Gorilla gorilla gorilla	0.125529 mm	0.125529 mm	0.125529 mm	85 kv	90 A	7.7 W	1500	MCZ - CC BY-NC	Citation: Lynn Lucas and Lynn Copes provided access to these data, the collection of which was funded by NSF DDIG #0925793 and Wenner Gren Foundation #8102. The files were downloaded from www.MorphoSource.org, Duke University.
MCZ-49006	doi:10.17602/M2/M2958	10.32 GB	MCZ 49006 mandible	Gorilla gorilla gorilla	0.07734 mm	0.07734 mm	0.07734 mm	85 kv	90 A	7.7 W	1000	MCZ - CC BY-NC	Citation: Lynn Lucas and Lynn Copes provided access to these data, the collection of which was funded by NSF DDIG #0925793 and Wenner Gren Foundation #8102. The files were downloaded from www.MorphoSource.org, Duke University.
MCZ-49635	doi:10.17602/M2/M5220	2.88 GB	MCZ 49635 skull	Cebus apella	0.059654 mm	0.059654 mm	0.059654 mm	90 kv	120 A	10.8 W	1100	MCZ - CC BY-NC	Citation: Lynn Lucas and Lynn Copes provided access to these data, the collection of which was funded by NSF DDIG #0925793 and Wenner Gren Foundation #8102. The files were downloaded from www.MorphoSource.org, Duke University.
MCZ-5016	doi:10.17602/M2/M4505	4.08 GB	MCZ 5016 skull	Propithecus diadema	0.048998 mm	0.048998 mm	0.048998 mm	80 kv	125 A	10 W	1000	MCZ - CC BY-NC	Citation: Lynn Lucas and Lynn Copes provided access to these data, the collection of which was funded by NSF DDIG #0925793 and Wenner Gren Foundation #8102. The files were downloaded from www.MorphoSource.org, Duke University.
MCZ-5057	doi:10.17602/M2/M4711	5.61 GB	MCZ 5057 skull	Pithecia monachus	0.050092 mm	0.050092 mm	0.050092 mm	80 kv	115 A	9.2 W	1050	MCZ - CC BY-NC	Citation: Lynn Lucas and Lynn Copes provided access to these data, the collection of which was funded by NSF DDIG #0925793 and Wenner Gren Foundation #8102. The files were downloaded from www.MorphoSource.org, Duke University.
MCZ-5071	doi:10.17602/M2/M2868	5.15 GB	MCZ 5071 skull	Aotus trivirgatus	0.040724 mm	0.040724 mm	0.040724 mm	80 kv	125 A	10 W	1000	MCZ - CC BY-NC	Citation: Lynn Lucas and Lynn Copes provided access to these data, the collection of which was funded by NSF DDIG #0925793 and Wenner Gren Foundation #8102. The files were downloaded from www.MorphoSource.org, Duke University.
MCZ-50958	doi:10.17602/M2/M4612	3.61 GB	MCZ 50958 mandible	Pongo pygmaeus	0.105227 mm	0.105227 mm	0.105227 mm	85 kv	90 A	7.7 W	1500	MCZ - CC BY-NC	Citation: Lynn Lucas and Lynn Copes provided access to these data, the collection of which was funded by NSF DDIG #0925793 and Wenner Gren Foundation #8102. The files were downloaded from www.MorphoSource.org, Duke University.
MCZ-5118	doi:10.17602/M2/M2717	11.96 GB	MCZ 5118 skull	Nycticebus coucang	0.029712 mm	0.029712 mm	0.029712 mm	80 kv	125 A	10 W	1000	MCZ - CC BY-NC	Citation: Lynn Lucas and Lynn Copes provided access to these data, the collection of which was funded by NSF DDIG #0925793 and Wenner Gren Foundation #8102. The files were downloaded from www.MorphoSource.org, Duke University.
MCZ-52557	doi:10.17602/M2/M4494	1.04 GB	MCZ 52557 skull	Saguinus sp.	0.040724 mm	0.040724 mm	0.040724 mm	80 kv	125 A	10 W	1000	MCZ - CC BY-NC	Citation: Lynn Lucas and Lynn Copes provided access to these data, the collection of which was funded by NSF DDIG #0925793 and Wenner Gren Foundation #8102. The files were downloaded from www.MorphoSource.org, Duke University.
MCZ-52558	doi:10.17602/M2/M4491	1.43 GB	MCZ 52558 skull	Saguinus sp.	0.040724 mm	0.040724 mm	0.040724 mm	80 kv	125 A	10 W	1000	MCZ - CC BY-NC	Citation: Lynn Lucas and Lynn Copes provided access to these data, the collection of which was funded by NSF DDIG #0925793 and Wenner Gren Foundation #8102. The files were downloaded from www.MorphoSource.org, Duke University.
MCZ-52608	doi:10.17602/M2/M2877	2.69 GB	MCZ 52608 skull	Aotus trivirgatus	0.040724 mm	0.040724 mm	0.040724 mm	80 kv	125 A	10 W	1000	MCZ - CC BY-NC	Citation: Lynn Lucas and Lynn Copes provided access to these data, the collection of which was funded by NSF DDIG #0925793 and Wenner Gren Foundation #8102. The files were downloaded from www.MorphoSource.org, Duke University.
MCZ-52614	doi:10.17602/M2/M4492	1.46 GB	MCZ 52614 skull	Saguinus sp.	0.040724 mm	0.040724 mm	0.040724 mm	80 kv	125 A	10 W	1000	MCZ - CC BY-NC	Citation: Lynn Lucas and Lynn Copes provided access to these data, the collection of which was funded by NSF DDIG #0925793 and Wenner Gren Foundation #8102. The files were downloaded from www.MorphoSource.org, Duke University.
MCZ-52615	doi:10.17602/M2/M4493	979.67 MB	MCZ 52615 skull	Saguinus sp.	0.040724 mm	0.040724 mm	0.040724 mm	80 kv	125 A	10 W	1000	MCZ - CC BY-NC	Citation: Lynn Lucas and Lynn Copes provided access to these data, the collection of which was funded by NSF DDIG #0925793 and Wenner Gren Foundation #8102. The files were downloaded from www.MorphoSource.org, Duke University.
MCZ-5323	doi:10.17602/M2/M2856	3.42 GB	MCZ 5323 skull	Alouatta palliata	0.079988 mm	0.079988 mm	0.079988 mm	85 kv	125 A	10.6 W	1100	MCZ - CC BY-NC	Citation: Lynn Lucas and Lynn Copes provided access to these data, the collection of which was funded by NSF DDIG #0925793 and Wenner Gren Foundation #8102. The files were downloaded from www.MorphoSource.org, Duke University.
MCZ-5324	doi:10.17602/M2/M2857	2.48 GB	MCZ 5324 skull	Alouatta palliata	0.079988 mm	0.079988 mm	0.079988 mm	85 kv	125 A	10.6 W	1100	MCZ - CC BY-NC	Citation: Lynn Lucas and Lynn Copes provided access to these data, the collection of which was funded by NSF DDIG #0925793 and Wenner Gren Foundation #8102. The files were downloaded from www.MorphoSource.org, Duke University.
MCZ-5325	doi:10.17602/M2/M2858	2.23 GB	MCZ 5325 skull	Alouatta palliata	0.079988 mm	0.079988 mm	0.079988 mm	85 kv	125 A	10.6 W	1100	MCZ - CC BY-NC	Citation: Lynn Lucas and Lynn Copes provided access to these data, the collection of which was funded by NSF DDIG #0925793 and Wenner Gren Foundation #8102. The files were downloaded from www.MorphoSource.org, Duke University.
MCZ-5327	doi:10.17602/M2/M2859	1.82 GB	MCZ 5327 skull	Alouatta palliata	0.079988 mm	0.079988 mm	0.079988 mm	85 kv	125 A	10.6 W	1100	MCZ - CC BY-NC	Citation: Lynn Lucas and Lynn Copes provided access to these data, the collection of which was funded by NSF DDIG #0925793 and Wenner Gren Foundation #8102. The files were downloaded from www.MorphoSource.org, Duke University.
MCZ-5328	doi:10.17602/M2/M2860	2.58 GB	MCZ 5328 skull	Alouatta palliata	0.079988 mm	0.079988 mm	0.079988 mm	85 kv	125 A	10 W	1100	MCZ - CC BY-NC	Citation: Lynn Lucas and Lynn Copes provided access to these data, the collection of which was funded by NSF DDIG #0925793 and Wenner Gren Foundation #8102. The files were downloaded from www.MorphoSource.org, Duke University.
MCZ-5329	doi:10.17602/M2/M2861	2.61 GB	MCZ 5329 skull	Alouatta palliata	0.079988 mm	0.079988 mm	0.079988 mm	85 kv	125 A	10.6 W	1100	MCZ - CC BY-NC	Citation: Lynn Lucas and Lynn Copes provided access to these data, the collection of which was funded by NSF DDIG #0925793 and Wenner Gren Foundation #8102. The files were downloaded from www.MorphoSource.org, Duke University.
MCZ-5331	doi:10.17602/M2/M2862	4.64 GB	MCZ 5331 skull	Alouatta palliata	0.079988 mm	0.079988 mm	0.079988 mm	85 kv	125 A	10.6 W	1100	MCZ - CC BY-NC	Citation: Lynn Lucas and Lynn Copes provided access to these data, the collection of which was funded by NSF DDIG #0925793 and Wenner Gren Foundation #8102. The files were downloaded from www.MorphoSource.org, Duke University.
MCZ-5332	doi:10.17602/M2/M5155	1.87 GB	MCZ 5332 skull	Cebus capucinus	0.079988 mm	0.079988 mm	0.079988 mm	80 kv	115 A	9.2 W	1100	MCZ - CC BY-NC	Citation: Lynn Lucas and Lynn Copes provided access to these data, the collection of which was funded by NSF DDIG #0925793 and Wenner Gren Foundation #8102. The files were downloaded from www.MorphoSource.org, Duke University.
MCZ-5336	doi:10.17602/M2/M2880	2.97 GB	MCZ 5336 skull	Ateles geoffroyi	0.079988 mm	0.079988 mm	0.079988 mm	80 kv	110 A	8.8 W	1100	MCZ - CC BY-NC	Citation: Lynn Lucas and Lynn Copes provided access to these data, the collection of which was funded by NSF DDIG #0925793 and Wenner Gren Foundation #8102. The files were downloaded from www.MorphoSource.org, Duke University.
MCZ-5338	doi:10.17602/M2/M2881	4.33 GB	MCZ 5338 skull	Ateles geoffroyi	0.063468 mm	0.063468 mm	0.063468 mm	90 kv	120 A	10.8 W	1100	MCZ - CC BY-NC	Citation: Lynn Lucas and Lynn Copes provided access to these data, the collection of which was funded by NSF DDIG #0925793 and Wenner Gren Foundation #8102. The files were downloaded from www.MorphoSource.org, Duke University.
MCZ-5340	doi:10.17602/M2/M2882	3.6 GB	MCZ 5340 skull	Ateles geoffroyi	0.063468 mm	0.063468 mm	0.063468 mm	90 kv	120 A	10.8 W	1100	MCZ - CC BY-NC	Citation: Lynn Lucas and Lynn Copes provided access to these data, the collection of which was funded by NSF DDIG #0925793 and Wenner Gren Foundation #8102. The files were downloaded from www.MorphoSource.org, Duke University.
MCZ-5344	doi:10.17602/M2/M2883	1.86 GB	MCZ 5344 skull	Ateles geoffroyi	0.079988 mm	0.079988 mm	0.079988 mm	80 kv	110 A	8.8 W	1100	MCZ - CC BY-NC	Citation: Lynn Lucas and Lynn Copes provided access to these data, the collection of which was funded by NSF DDIG #0925793 and Wenner Gren Foundation #8102. The files were downloaded from www.MorphoSource.org, Duke University.
MCZ-5345	doi:10.17602/M2/M2884	2.27 GB	MCZ 5345 skull	Ateles geoffroyi	0.079988 mm	0.079988 mm	0.079988 mm	80 kv	110 A	8.8 W	1100	MCZ - CC BY-NC	Citation: Lynn Lucas and Lynn Copes provided access to these data, the collection of which was funded by NSF DDIG #0925793 and Wenner Gren Foundation #8102. The files were downloaded from www.MorphoSource.org, Duke University.
MCZ-5346	doi:10.17602/M2/M2885	2.44 GB	MCZ 5346 skull	Ateles geoffroyi	0.079988 mm	0.079988 mm	0.079988 mm	80 kv	110 A	8.8 W	1100	MCZ - CC BY-NC	Citation: Lynn Lucas and Lynn Copes provided access to these data, the collection of which was funded by NSF DDIG #0925793 and Wenner Gren Foundation #8102. The files were downloaded from www.MorphoSource.org, Duke University.
MCZ-5348	doi:10.17602/M2/M2886	2.79 GB	MCZ 5348 skull	Ateles geoffroyi	0.079988 mm	0.079988 mm	0.079988 mm	80 kv	110 A	8.8 W	1100	MCZ - CC BY-NC	Citation: Lynn Lucas and Lynn Copes provided access to these data, the collection of which was funded by NSF DDIG #0925793 and Wenner Gren Foundation #8102. The files were downloaded from www.MorphoSource.org, Duke University.
MCZ-5349	doi:10.17602/M2/M2887	4.16 GB	MCZ 5349 skull	Ateles geoffroyi	0.063468 mm	0.063468 mm	0.063468 mm	90 kv	120 A	10.8 W	1100	MCZ - CC BY-NC	Citation: Lynn Lucas and Lynn Copes provided access to these data, the collection of which was funded by NSF DDIG #0925793 and Wenner Gren Foundation #8102. The files were downloaded from www.MorphoSource.org, Duke University.
MCZ-5350	doi:10.17602/M2/M2888	2.36 GB	MCZ 5350 skull	Ateles geoffroyi	0.079988 mm	0.079988 mm	0.079988 mm	80 kv	110 A	8.8 W	1100	MCZ - CC BY-NC	Citation: Lynn Lucas and Lynn Copes provided access to these data, the collection of which was funded by NSF DDIG #0925793 and Wenner Gren Foundation #8102. The files were downloaded from www.MorphoSource.org, Duke University.
MCZ-5351	doi:10.17602/M2/M2889	4.16 GB	MCZ 5351 skull	Ateles geoffroyi	0.063468 mm	0.063468 mm	0.063468 mm	90 kv	120 A	10.8 W	1100	MCZ - CC BY-NC	Citation: Lynn Lucas and Lynn Copes provided access to these data, the collection of which was funded by NSF DDIG #0925793 and Wenner Gren Foundation #8102. The files were downloaded from www.MorphoSource.org, Duke University.
MCZ-5352	doi:10.17602/M2/M2890	4.31 GB	MCZ 5352 skull	Ateles geoffroyi	0.079988 mm	0.079988 mm	0.079988 mm	80 kv	110 A	8.8 W	1100	MCZ - CC BY-NC	Citation: Lynn Lucas and Lynn Copes provided access to these data, the collection of which was funded by NSF DDIG #0925793 and Wenner Gren Foundation #8102. The files were downloaded from www.MorphoSource.org, Duke University.
MCZ-5353	doi:10.17602/M2/M2891	3.35 GB	MCZ 5353 skull	Ateles geoffroyi	0.079988 mm	0.079988 mm	0.079988 mm	80 kv	110 A	8.8 W	1100	MCZ - CC BY-NC	Citation: Lynn Lucas and Lynn Copes provided access to these data, the collection of which was funded by NSF DDIG #0925793 and Wenner Gren Foundation #8102. The files were downloaded from www.MorphoSource.org, Duke University.
MCZ-5354	doi:10.17602/M2/M2915	3.15 GB	MCZ 5354 skull	Ateles geoffroyi	0.079988 mm	0.079988 mm	0.079988 mm	80 kv	110 A	8.8 W	1100	MCZ - CC BY-NC	Citation: Lynn Lucas and Lynn Copes provided access to these data, the collection of which was funded by NSF DDIG #0925793 and Wenner Gren Foundation #8102. The files were downloaded from www.MorphoSource.org, Duke University.
MCZ-5355	doi:10.17602/M2/M2916	5.28 GB	MCZ 5355 skull	Ateles geoffroyi	0.079988 mm	0.079988 mm	0.079988 mm	80 kv	110 A	8.8 W	1100	MCZ - CC BY-NC	Citation: Lynn Lucas and Lynn Copes provided access to these data, the collection of which was funded by NSF DDIG #0925793 and Wenner Gren Foundation #8102. The files were downloaded from www.MorphoSource.org, Duke University.
MCZ-5547	doi:10.17602/M2/M5087	5.45 GB	MCZ 5547 skull	Miopithecus talapoin	0.050092 mm	0.050092 mm	0.050092 mm	80 kv	125 A	10 W	1000	MCZ - CC BY-NC	Citation: Lynn Lucas and Lynn Copes provided access to these data, the collection of which was funded by NSF DDIG #0925793 and Wenner Gren Foundation #8102. The files were downloaded from www.MorphoSource.org, Duke University.
MCZ-58139	doi:10.17602/M2/M2737	4.3 GB	MCZ 58139 skull	Varecia variegata rubra	0.043498 mm	0.043498 mm	0.043498 mm	80 kv	125 A	10 W	1000	MCZ - CC BY-NC	Citation: Lynn Lucas and Lynn Copes provided access to these data, the collection of which was funded by NSF DDIG #0925793 and Wenner Gren Foundation #8102. The files were downloaded from www.MorphoSource.org, Duke University.
MCZ-59274	doi:10.17602/M2/M2738	4.19 GB	MCZ 59274 skull	Varecia variegata variegata	0.054763 mm	0.054763 mm	0.054763 mm	80 kv	125 A	10 W	1000	MCZ - CC BY-NC	Citation: Lynn Lucas and Lynn Copes provided access to these data, the collection of which was funded by NSF DDIG #0925793 and Wenner Gren Foundation #8102. The files were downloaded from www.MorphoSource.org, Duke University.
MCZ-59277	doi:10.17602/M2/M2739	3.07 GB	MCZ 59277 skull	Varecia variegata rubra	0.043498 mm	0.043498 mm	0.043498 mm	80 kv	125 A	10 W	1000	MCZ - CC BY-NC	Citation: Lynn Lucas and Lynn Copes provided access to these data, the collection of which was funded by NSF DDIG #0925793 and Wenner Gren Foundation #8102. The files were downloaded from www.MorphoSource.org, Duke University.
MCZ-6001	doi:10.17602/M2/M2863	1.6 GB	MCZ 6001 skull	Alouatta palliata	0.079988 mm	0.079988 mm	0.079988 mm	85 kv	125 A	10.6 W	1100	MCZ - CC BY-NC	Citation: Lynn Lucas and Lynn Copes provided access to these data, the collection of which was funded by NSF DDIG #0925793 and Wenner Gren Foundation #8102. The files were downloaded from www.MorphoSource.org, Duke University.
MCZ-6028	doi:10.17602/M2/M5144	4.39 GB	MCZ 6028 skull	Chiropotes satanas	0.052569 mm	0.052569 mm	0.052569 mm	80 kv	115 A	9.2 W	1100	MCZ - CC BY-NC	Citation: Lynn Lucas and Lynn Copes provided access to these data, the collection of which was funded by NSF DDIG #0925793 and Wenner Gren Foundation #8102. The files were downloaded from www.MorphoSource.org, Duke University.
MCZ-61273	doi:10.17602/M2/M3045	4.89 GB	MCZ 61273 skull	Macaca fuscata	0.090751 mm	0.090751 mm	0.090751 mm	80 kv	110 A	8.8 W	1050	MCZ - CC BY-NC	Citation: Lynn Lucas and Lynn Copes provided access to these data, the collection of which was funded by NSF DDIG #0925793 and Wenner Gren Foundation #8102. The files were downloaded from www.MorphoSource.org, Duke University.
MCZ-61414	doi:10.17602/M2/M3053	2.88 GB	MCZ 61414 skull	Macaca mulatta	0.090751 mm	0.090751 mm	0.090751 mm	80 kv	110 A	8.8 W	1050	MCZ - CC BY-NC	Citation: Lynn Lucas and Lynn Copes provided access to these data, the collection of which was funded by NSF DDIG #0925793 and Wenner Gren Foundation #8102. The files were downloaded from www.MorphoSource.org, Duke University.
MCZ-6209	doi:10.17602/M2/M2623	2.16 GB	MCZ 6209 skull	Cercocebus albigena	0.08971 mm	0.08971 mm	0.08971 mm	80 kv	125 A	10 W	1100	MCZ - CC BY-NC	Citation: Lynn Lucas and Lynn Copes provided access to these data, the collection of which was funded by NSF DDIG #0925793 and Wenner Gren Foundation #8102. The files were downloaded from www.MorphoSource.org, Duke University.
MCZ-6244	doi:10.17602/M2/M4393	3.21 GB	MCZ 6244 cranium	Pan troglodytes	0.106542 mm	0.106542 mm	0.106542 mm	80 kv	120 A	9.6 W	1500	MCZ - CC BY-NC	Citation: Lynn Lucas and Lynn Copes provided access to these data, the collection of which was funded by NSF DDIG #0925793 and Wenner Gren Foundation #8102. The files were downloaded from www.MorphoSource.org, Duke University.
MCZ-6244	doi:10.17602/M2/M4394	2.91 GB	MCZ 6244 mandible	Pan troglodytes	0.081907 mm	0.081907 mm	0.081907 mm	80 kv	120 A	9.6 W	1000	MCZ - CC BY-NC	Citation: Lynn Lucas and Lynn Copes provided access to these data, the collection of which was funded by NSF DDIG #0925793 and Wenner Gren Foundation #8102. The files were downloaded from www.MorphoSource.org, Duke University.
MCZ-62639	doi:10.17602/M2/M2588	2.47 GB	MCZ 62639 skull	Cercocebus torquatus	0.090751 mm	0.090751 mm	0.090751 mm	85 kv	125 A	10.6 W	1100	MCZ - CC BY-NC	Citation: Lynn Lucas and Lynn Copes provided access to these data, the collection of which was funded by NSF DDIG #0925793 and Wenner Gren Foundation #8102. The files were downloaded from www.MorphoSource.org, Duke University.
MCZ-6291	doi:10.17602/M2/M2705	3.44 GB	MCZ 6291 skull	Hapalemur griseus	0.038685 mm	0.038685 mm	0.038685 mm	80 kv	125 A	10 W	1000	MCZ - CC BY-NC	Citation: Lynn Lucas and Lynn Copes provided access to these data, the collection of which was funded by NSF DDIG #0925793 and Wenner Gren Foundation #8102. The files were downloaded from www.MorphoSource.org, Duke University.
MCZ-6367	doi:10.17602/M2/M3047	2.28 GB	MCZ 6367 skull	Macaca mulatta	0.090751 mm	0.090751 mm	0.090751 mm	80 kv	110 A	8.8 W	1050	MCZ - CC BY-NC	Citation: Lynn Lucas and Lynn Copes provided access to these data, the collection of which was funded by NSF DDIG #0925793 and Wenner Gren Foundation #8102. The files were downloaded from www.MorphoSource.org, Duke University.
MCZ-6377	doi:10.17602/M2/M4442	3.53 GB	MCZ 6377 skull	Therapithecus gelada	0.057738 mm	0.057738 mm	0.057738 mm	85 kv	125 A	10 W	1100	MCZ - CC BY-NC	Citation: Lynn Lucas and Lynn Copes provided access to these data, the collection of which was funded by NSF DDIG #0925793 and Wenner Gren Foundation #8102. The files were downloaded from www.MorphoSource.org, Duke University.
MCZ-64170	doi:10.17602/M2/M2834	3.94 GB	MCZ 64170 skull	Galago alleni	0.027498 mm	0.027498 mm	0.027498 mm	80 kv	125 A	10 W	1000	MCZ - CC BY-NC	Citation: Lynn Lucas and Lynn Copes provided access to these data, the collection of which was funded by NSF DDIG #0925793 and Wenner Gren Foundation #8102. The files were downloaded from www.MorphoSource.org, Duke University.
MCZ-6923	doi:10.17602/M2/M4721	7.35 GB	MCZ 6923 skull	Perodicticus potto	0.039517 mm	0.039517 mm	0.039517 mm	80 kv	125 A	10 W	1000	MCZ - CC BY-NC	Citation: Lynn Lucas and Lynn Copes provided access to these data, the collection of which was funded by NSF DDIG #0925793 and Wenner Gren Foundation #8102. The files were downloaded from www.MorphoSource.org, Duke University.
MCZ-7072	doi:10.17602/M2/M3055	3.11 GB	MCZ 7072 skull	Macaca sylvanus	0.087712 mm	0.087712 mm	0.087712 mm	80 kv	110 A	8.8 W	1100	MCZ - CC BY-NC	Citation: Lynn Lucas and Lynn Copes provided access to these data, the collection of which was funded by NSF DDIG #0925793 and Wenner Gren Foundation #8102. The files were downloaded from www.MorphoSource.org, Duke University.
MCZ-7088	doi:10.17602/M2/M2927	4.02 GB	MCZ 7088 skull	Cercopithecus mitis	0.079988 mm	0.079988 mm	0.079988 mm	80 kv	125 A	10 W	1100	MCZ - CC BY-NC	Citation: Lynn Lucas and Lynn Copes provided access to these data, the collection of which was funded by NSF DDIG #0925793 and Wenner Gren Foundation #8102. The files were downloaded from www.MorphoSource.org, Duke University.
MCZ-7098	doi:10.17602/M2/M3057	2.12 GB	MCZ 7098 skull	Macaca sylvanus	0.090751 mm	0.090751 mm	0.090751 mm	80 kv	110 A	8.8 W	1050	MCZ - CC BY-NC	Citation: Lynn Lucas and Lynn Copes provided access to these data, the collection of which was funded by NSF DDIG #0925793 and Wenner Gren Foundation #8102. The files were downloaded from www.MorphoSource.org, Duke University.
MCZ-7165	doi:10.17602/M2/M5204	2.48 GB	MCZ BOM-7165 skull	Callithrix sp.	0.040724 mm	0.040724 mm	0.040724 mm	80 kv	125 A	10 W	1000	MCZ - CC BY-NC	Citation: Lynn Lucas and Lynn Copes provided access to these data, the collection of which was funded by NSF DDIG #0925793 and Wenner Gren Foundation #8102. The files were downloaded from www.MorphoSource.org, Duke University.
MCZ-7317	doi:10.17602/M2/M5154	3.39 GB	MCZ 7317 skull	Cebus capucinus	0.079988 mm	0.079988 mm	0.079988 mm	80 kv	115 A	9.2 W	1100	MCZ - CC BY-NC	Citation: Lynn Lucas and Lynn Copes provided access to these data, the collection of which was funded by NSF DDIG #0925793 and Wenner Gren Foundation #8102. The files were downloaded from www.MorphoSource.org, Duke University.
MCZ-7321	doi:10.17602/M2/M5146	2.9 GB	MCZ 7321 skull	Cebus capucinus	0.079988 mm	0.079988 mm	0.079988 mm	80 kv	115 A	9.2 W	1100	MCZ - CC BY-NC	Citation: Lynn Lucas and Lynn Copes provided access to these data, the collection of which was funded by NSF DDIG #0925793 and Wenner Gren Foundation #8102. The files were downloaded from www.MorphoSource.org, Duke University.
MCZ-7322	doi:10.17602/M2/M5153	2.59 GB	MCZ 7322 skull	Cebus capucinus	0.079988 mm	0.079988 mm	0.079988 mm	80 kv	115 A	9.2 W	1100	MCZ - CC BY-NC	Citation: Lynn Lucas and Lynn Copes provided access to these data, the collection of which was funded by NSF DDIG #0925793 and Wenner Gren Foundation #8102. The files were downloaded from www.MorphoSource.org, Duke University.
MCZ-7323	doi:10.17602/M2/M5152	2.81 GB	MCZ 7323 skull	Cebus capucinus	0.079988 mm	0.079988 mm	0.079988 mm	80 kv	115 A	9.2 W	1100	MCZ - CC BY-NC	Citation: Lynn Lucas and Lynn Copes provided access to these data, the collection of which was funded by NSF DDIG #0925793 and Wenner Gren Foundation #8102. The files were downloaded from www.MorphoSource.org, Duke University.
MCZ-8039	doi:10.17602/M2/M2713	7.71 GB	MCZ 8039 skull	Avahi laniger	0.03124 mm	0.03124 mm	0.03124 mm	80 kv	125 A	10 W	1000	MCZ - CC BY-NC	Citation: Lynn Lucas and Lynn Copes provided access to these data, the collection of which was funded by NSF DDIG #0925793 and Wenner Gren Foundation #8102. The files were downloaded from www.MorphoSource.org, Duke University.
MCZ-8041	doi:10.17602/M2/M2723	6.13 GB	MCZ 8041 skull	Propithecus verreauxi coquereli	0.049058 mm	0.049058 mm	0.049058 mm	80 kv	125 A	10 W	1000	MCZ - CC BY-NC	Citation: Lynn Lucas and Lynn Copes provided access to these data, the collection of which was funded by NSF DDIG #0925793 and Wenner Gren Foundation #8102. The files were downloaded from www.MorphoSource.org, Duke University.
MCZ-8042	doi:10.17602/M2/M4504	3.56 GB	MCZ 8042 skull	Propithecus diadema edwardsi	0.048998 mm	0.048998 mm	0.048998 mm	80 kv	125 A	10 W	1000	MCZ - CC BY-NC	Citation: Lynn Lucas and Lynn Copes provided access to these data, the collection of which was funded by NSF DDIG #0925793 and Wenner Gren Foundation #8102. The files were downloaded from www.MorphoSource.org, Duke University.
MCZ-8043	doi:10.17602/M2/M2836	4.37 GB	MCZ 8043 skull	Lemur catta	0.044601 mm	0.044601 mm	0.044601 mm	80 kv	125 A	10 W	1000	MCZ - CC BY-NC	Citation: Lynn Lucas and Lynn Copes provided access to these data, the collection of which was funded by NSF DDIG #0925793 and Wenner Gren Foundation #8102. The files were downloaded from www.MorphoSource.org, Duke University.
MCZ-8044	doi:10.17602/M2/M2822	3.03 GB	MCZ 8044 skull	Eulemur fulvus fulvus	0.045991 mm	0.045991 mm	0.045991 mm	80 kv	125 A	10 W	1000	MCZ - CC BY-NC	Citation: Lynn Lucas and Lynn Copes provided access to these data, the collection of which was funded by NSF DDIG #0925793 and Wenner Gren Foundation #8102. The files were downloaded from www.MorphoSource.org, Duke University.
MCZ-8304	doi:10.17602/M2/M4887	2.65 GB	MCZ 8304 cranium	Papio doguera	0.117823 mm	0.117823 mm	0.117823 mm	85 kv	125 A	10 W	1100	MCZ - CC BY-NC	Citation: Lynn Lucas and Lynn Copes provided access to these data, the collection of which was funded by NSF DDIG #0925793 and Wenner Gren Foundation #8102. The files were downloaded from www.MorphoSource.org, Duke University.
MCZ-8304	doi:10.17602/M2/M4888	4.77 GB	MCZ 8304 mandible	Papio doguera	0.085431 mm	0.085431 mm	0.085431 mm	85 kv	125 A	10 W	1100	MCZ - CC BY-NC	Citation: Lynn Lucas and Lynn Copes provided access to these data, the collection of which was funded by NSF DDIG #0925793 and Wenner Gren Foundation #8102. The files were downloaded from www.MorphoSource.org, Duke University.
MCZ-8461	doi:10.17602/M2/M3027	3.75 GB	MCZ 8461 skull	Macaca fascicularis	0.061559 mm	0.061559 mm	0.061559 mm	80 kv	125 A	10 W	1050	MCZ - CC BY-NC	Citation: Lynn Lucas and Lynn Copes provided access to these data, the collection of which was funded by NSF DDIG #0925793 and Wenner Gren Foundation #8102. The files were downloaded from www.MorphoSource.org, Duke University.
MCZ-8466	doi:10.17602/M2/M4889	1.03 GB	MCZ 8466 skull	Papio doguera	0.07994 mm	0.07994 mm	0.07994 mm	85 kv	125 A	10 W	1100	MCZ - CC BY-NC	Citation: Lynn Lucas and Lynn Copes provided access to these data, the collection of which was funded by NSF DDIG #0925793 and Wenner Gren Foundation #8102. The files were downloaded from www.MorphoSource.org, Duke University.
MCZ-8471	doi:10.17602/M2/M2864	1.44 GB	MCZ 8471 skull	Alouatta palliata	0.079988 mm	0.079988 mm	0.079988 mm	85 kv	125 A	10.6 W	1100	MCZ - CC BY-NC	Citation: Lynn Lucas and Lynn Copes provided access to these data, the collection of which was funded by NSF DDIG #0925793 and Wenner Gren Foundation #8102. The files were downloaded from www.MorphoSource.org, Duke University.
MCZ-8472	doi:10.17602/M2/M2869	6.15 GB	MCZ 8472 skull	Aotus trivirgatus	0.040724 mm	0.040724 mm	0.040724 mm	80 kv	125 A	10 W	1000	MCZ - CC BY-NC	Citation: Lynn Lucas and Lynn Copes provided access to these data, the collection of which was funded by NSF DDIG #0925793 and Wenner Gren Foundation #8102. The files were downloaded from www.MorphoSource.org, Duke University.
MCZ-8473	doi:10.17602/M2/M2838	7.25 GB	MCZ 8473 skull	Lemur catta	0.044601 mm	0.044601 mm	0.044601 mm	80 kv	125 A	10 W	1000	MCZ - CC BY-NC	Citation: Lynn Lucas and Lynn Copes provided access to these data, the collection of which was funded by NSF DDIG #0925793 and Wenner Gren Foundation #8102. The files were downloaded from www.MorphoSource.org, Duke University.
MCZ-9493	doi:10.17602/M2/M4392	3.69 GB	MCZ 9493 skull	Pan troglodytes	0.109857 mm	0.109857 mm	0.109857 mm	80 kv	120 A	9.6 W	1500	MCZ - CC BY-NC	Citation: Lynn Lucas and Lynn Copes provided access to these data, the collection of which was funded by NSF DDIG #0925793 and Wenner Gren Foundation #8102. The files were downloaded from www.MorphoSource.org, Duke University.
MCZ-B-8042	doi:10.17602/M2/M2878	2.29 GB	MCZ B-8042 skull	Aotus trivirgatus	0.040724 mm	0.040724 mm	0.040724 mm	80 kv	125 A	10 W	1000	MCZ - CC BY-NC	Citation: Lynn Lucas and Lynn Copes provided access to these data, the collection of which was funded by NSF DDIG #0925793 and Wenner Gren Foundation #8102. The files were downloaded from www.MorphoSource.org, Duke University.
MCZ-B-8043	doi:10.17602/M2/M2879	2.06 GB	MCZ B-8043 skull	Aotus trivirgatus	0.040724 mm	0.040724 mm	0.040724 mm	80 kv	125 A	10 W	1000	MCZ - CC BY-NC	Citation: Lynn Lucas and Lynn Copes provided access to these data, the collection of which was funded by NSF DDIG #0925793 and Wenner Gren Foundation #8102. The files were downloaded from www.MorphoSource.org, Duke University.
Note that each dataset is tagged with a doi that serves as an instant and permanent link to each dataset and improves the ease of accessing the sample.													

**Table 2:** Voxel size and relative error for Nikon, X-Tek XHT 225 ST scanner at Duke University (results comparable for Harvard’s X-Tek HMX 225 ST μCT scanner).

**Voxel Size μm**	**Relative Error (%)**
5.00274654	0.143102011
6.002784351	0.121112981
7.004121857	0.141531687
8.00419928	0.110114834
9.004866272	0.124254864
10.0052979	0.122683947
15.00729757	−0.009449712
20.00988969	0.099114265
30.01900974	−0.147461222
40.02104118	−0.157728707
50.04978064	−0.236779795
60.04728152	−0.157728707
70.04123235	−0.157728707

## References

[d1] MorphoSource Copes L. E. Lucas L. M. Thostenson J. O. Hoekstra H. E. Boyer D. M. 2015 http://dx.doi.org/10.17602/M2/M2938

[d2] MorphoSource Copes L. E. Lucas L. M. Thostenson J. O. Hoekstra H. E. Boyer D. M. 2015 http://dx.doi.org/10.17602/M2/M2894

[d3] MorphoSource Copes L. E. Lucas L. M. Thostenson J. O. Hoekstra H. E. Boyer D. M. 2015 http://dx.doi.org/10.17602/M2/M4705

[d4] MorphoSource Copes L. E. Lucas L. M. Thostenson J. O. Hoekstra H. E. Boyer D. M. 2015 http://dx.doi.org/10.17602/M2/M2844

[d5] MorphoSource Copes L. E. Lucas L. M. Thostenson J. O. Hoekstra H. E. Boyer D. M. 2015 http://dx.doi.org/10.17602/M2/M2714

[d6] Dryad Digital Repository Copes L. E. Lucas L. M. Boyer D. M. 2015 http://dx.doi.org/10.5061/dryad.dm57j

